# Synthesis, Characterisation and Reactivity of Copper(I) Amide Complexes and Studies on Their Role in the Modified Ullmann Amination Reaction

**DOI:** 10.1002/chem.201405699

**Published:** 2015-03-18

**Authors:** Simon Sung, D Christopher Braddock, Alan Armstrong, Colin Brennan, David Sale, Andrew J P White, Robert P Davies

**Affiliations:** [a] Department of Chemistry, Imperial College LondonSouth Kensington, London, SW7 2AZ (UK); [b] Process Studies Group, Syngenta, Jealott*s Hill Research CentreBracknell, Berkshire RG42 6EY (UK)

**Keywords:** amination, copper, homogeneous catalysis, structure elucidation, Ullmann reaction

## Abstract

A series of copper(I) alkylamide complexes have been synthesised; copper(I) dicyclohexylamide (**1**), copper(I) 2,2,6,6-tetramethylpiperidide (**2**), copper(I) pyrrolidide (**3**), copper(I) piperidide (**4**), and copper(I) benzylamide (**5**). Their solid-state structures and structures in [D_6_]benzene solution are characterised, with the aggregation state in solution determined by a combination of DOSY NMR spectroscopy and DFT calculations. Complexes **1**, **2** and **4** are shown to exist as tetramers in the solid state by X-ray crystallography. In [D_6_]benzene solution, complexes **1**, **2** and **5** were found by using ^1^H DOSY NMR to exist in rapid equilibrium between aggregates with average aggregation numbers of 2.5, 2.4 and 3.3, respectively, at 0.05 m concentration. Conversely, distinct trimeric, tetrameric and pentameric forms of **3** and **4** were distinguishable by one-dimensional ^1^H and ^1^H DOSY NMR spectroscopy. Complexes **3**–**5** are found to react stoichiometrically with iodobenzene, in the presence or absence of 1,10-phenanthroline as an ancillary ligand, to give arylamine products indicative of their role as potential intermediates in the modified Ullmann reaction. The role of phenanthroline has also been explored both in the stoichiometric reaction and in the catalytic Ullmann protocol.

## Introduction

**Scheme 1:**
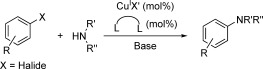
General reaction scheme of the modified Ullmann reaction

Copper mediated cross-coupling reactions between aryl halides and amines to form carbon-nitrogen bonds were first reported over a century ago by Ullmann when he demonstrated the preparation of 2-(*N*-phenylamino)benzoic acid from *o*-chlorobenzoic acid and aniline in the presence of stoichiometric copper metal.[1] However, this classical method for cross-coupling aryl halides and amines has many drawbacks including high (stoichiometric) copper metal loadings, high reaction temperatures and also long reaction times.[2, 3] More recently new protocols that incorporate the use of bidentate ligands such as 1,10-phenanthroline (phen)[4–9] have enabled these reactions to be carried out at lower reaction temperatures (≤110 °C) with lower copper loadings (≤10 %).[10–13] This improved copper-catalysed reaction is commonly referred to as the modified Ullmann amination reaction (Scheme 1).

The relatively low cost of copper, use of cheap and low molecular weight nitrogen- and oxygen-based ligand systems, and good functional group tolerance have made the modified Ullmann reaction an attractive and complementary method to palladium assisted Buchwald–Hartwig amination.[14, 15] The modified Ullmann reaction has been employed in a range of synthetic endeavours, including the total synthesis of the natural product martinellic acid,[16, 17] and other compounds with biological activity, such as SB-214857[18] and Benzolactam-V8.[19] Nevertheless, one of the present disadvantages of the modified Ullmann reaction remains its poor reaction scope. Thus reactions involving aryl bromides typically require reaction temperatures greater than 90 °C to achieve cross coupling, and aryl chlorides additionally require strong electron-withdrawing groups at the *ortho* and/or *para* positions for good reactivity.[20–25] Another significant shortcoming of the modified Ullmann reaction is that the intermediates and steps involved in the reaction mechanism are still not fully understood, despite some recent progress in this area.[3, 5, 6, 26–38] Ideally, a rational approach based on a fundamental understanding of the mechanism is crucial for the development of new catalysts and improvement of existing systems.

**Scheme 2:**
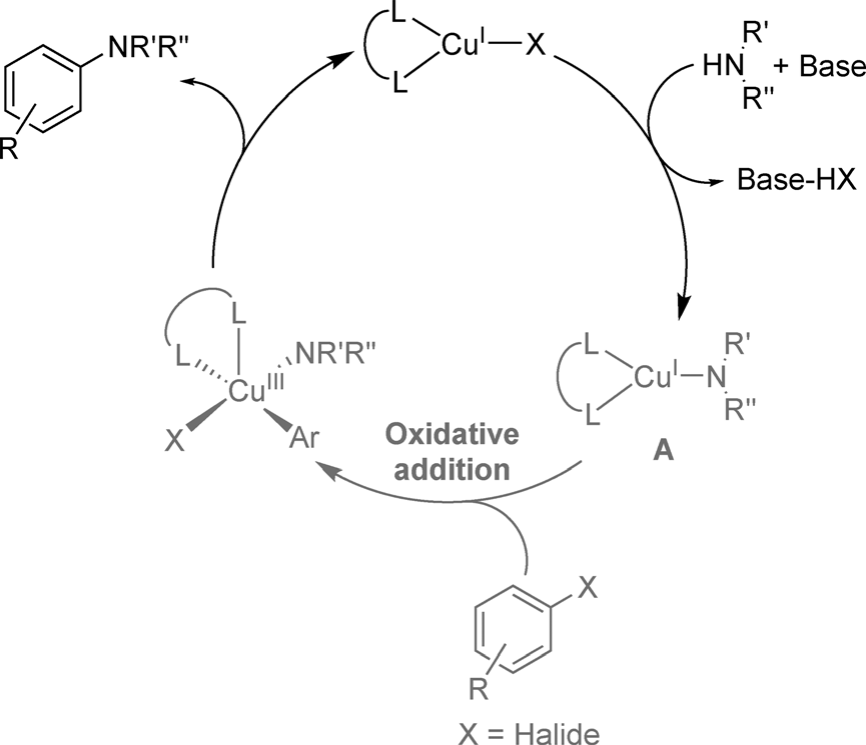
General catalytic cycle for the modified Ullmann amination reaction highlighting the oxidative addition aryl halide activation step.

The rate determining step in the Ullmann reaction is commonly proposed to be aryl halide activation (see Scheme 2 for a proposed catalytic cycle).[3] Identification and study of the catalyst resting state involved in this activation is therefore crucial to building a more thorough understanding of the mechanism. In the absence of ancillary ligands, Paine and co-workers first showed that a copper(I) amide species, CuNR_2_, is initially formed and this then undergoes aryl halide activation.[39] More recently the groups of Jutand[32–34] and Hartwig[30] determined that when an ancillary ligand is present the catalyst resting state contains the ligand, copper(I) and deprotonated amine in a 1:1:1 ratio (Complex **A** in Scheme 2). Moreover, additional experimental results imply that aryl halide activation proceeds via direct oxidative addition with the copper(I) amide resting state.[30, 32–34]

However, despite the purported importance of copper(I) amides in Ullmann reactions (complex **A** in Scheme 2 and CuNR_2_ in non-ligated systems), there remain relatively few detailed studies on the synthesis and structures of these species.[40–47] In addition, the reactivity of isolated copper(I) amides in aryl halide amination remains little explored, and a successful reaction between a copper(I) amide and an aryl halide in the absence of additional ligand has yet to be reported. Previous studies have been somewhat limited in scope and all show no reactivity for non-ligated copper(I) amide aggregates with aryl halides.[27, 30]

Herein we report the synthesis and detailed characterisation of five copper(I) alkylamide complexes, with particular emphasis on their solid-state structures and solution equilibrium. The competency of these complexes as ‘ligand-free’ intermediates within the modified Ullmann amination catalytic cycle has also been investigated both in benzene and DMSO solutions. In addition, the interaction of copper(I) amides with the phen ligand has been studied both for its influence on solution structure and effect on catalytic performance. Together these studies seek to improve current understanding of the mechanism of the Ullmann amination reaction and in particular how the identity of the amide nucleophile and ancillary ligand can affect the structure and reactivity of the catalyst resting state.

## Results and Discussion

### Synthesis of copper(I) amide complexes

Although copper(I) amide complexes ([Cu(NRR′)]_*n*_) were first reported almost a century ago,[48] there are relatively few literature reports detailing their synthesis, structural characterisation or reactivity.[40–47] This can perhaps be attributed, at least in part, to the often high air and moisture sensitivity of these species.

**Scheme 3:**
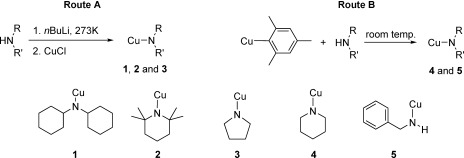
Synthesis of copper(I) amide complexes 1–5.

The amines studied herein (dicyclohexylamine, tetramethylpiperidine (TMP), pyrrolidine, piperidine and benzylamine) were chosen to provide a range of steric bulk and also display varied performance in the catalytic coupling reaction, with primary amines known to provide better yields and reaction rates than acyclic secondary amines.[14] The copper amide complexes were prepared from the parent amines either via the lithium amide complex (Route A, Scheme 3) or by direct reaction of the amine with copper(I) mesityl (CuMes, Route B, Scheme 3).

Copper(I) dicyclohexylamide ([Cu(NCy_2_)]_*n*_; **1**), copper(I) 2,2,6,6-tetramethylpiperidide ([Cu(TMP)]_*n*_; **2**), and copper(I) pyrrolidide ([Cu(NC_4_H_8_)]_*n*_; **3**) were synthesised by a metathesis reaction between copper(I) chloride and the corresponding lithium amide (Route A, Scheme 3). Complex **3** has previously been prepared using an analogous synthetic route,[46] although copper(I) amides **1** and **2** have not been reported before in the literature. An alternative preparative route employing CuMes as a copper metalation reagent (Route B, Scheme 3) was used to prepare copper(I) piperidide ([Cu(NC_5_H_10_)]_*n*_; **4**), and copper(I) benzylamide ([Cu(N(H)CH_2_Ph)]_*n*_; **5**).[41, 42, 49] This synthetic route avoided the formation of halide salt byproducts and allowed preparation of the copper(I) amides in high purity. It is interesting to note that the success of Route B was very dependent upon the parent amine used, and despite several attempts it was not possible to prepare complexes **1** and **2** via this route. An insight into why this might be was obtained by studying the reaction of piperidine with CuMes (see below).

### Solid-state structures of copper(I) amide complexes

Complexes **1**–**4** were obtained as single crystals directly from their respective reaction mixtures, with complexes **1**, **2** and **4** being structurally characterised by single-crystal X-ray diffraction. Floriani and co-workers previously reported the solid-state structure of complex **3**, which is a tetramer, [Cu(NC_4_H_8_)]_4_.[46] Complex **5** is unstable in solution, rapidly decomposing to give an orange solid, and thus it was not possible to obtain structural data for this complex. All new copper(I) amides were found to exist as tetramers in the solid state, containing amido-nitrogen atoms bridging pairs of copper(I) ions to form eight-membered Cu_4_N_4_ rings. However, the planarity of the Cu_4_N_4_ unit and linearity of the N-Cu-N bond angle varies significantly between the solid-state structures.

**Figure 1:**
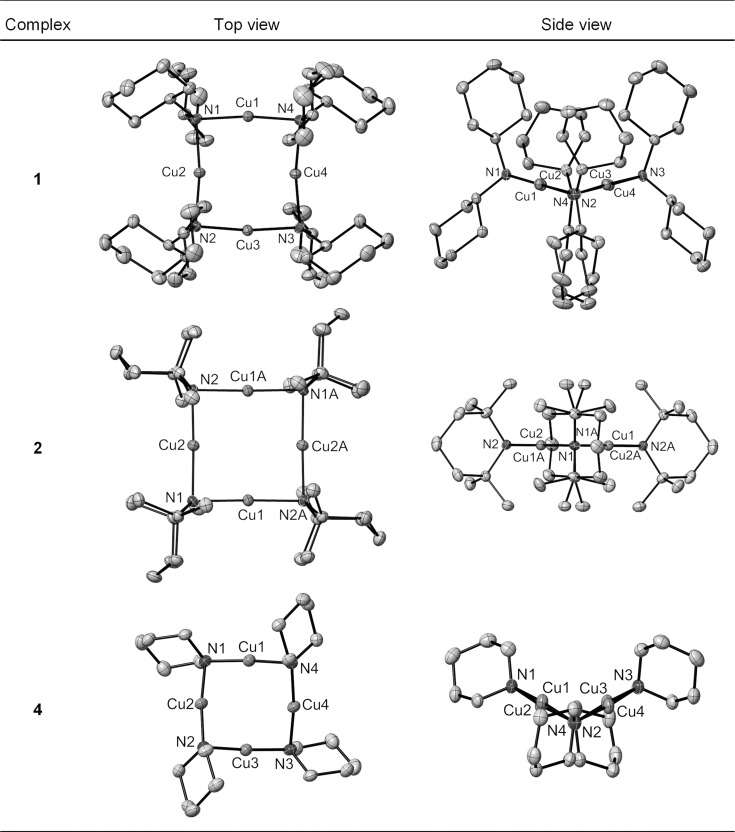
Views of solid-state structures of complexes 1, 2 and 4 from orthogonal directions, determined by X-ray crystallography. Thermal ellipsoids are set at 50 % probability. Hydrogen atoms are not shown for clarity.

The structures of all new complexes are shown in Figure 1 with selected bond lengths and bond angles listed in Table 1. In **1** and **4**, the eight-membered Cu_4_N_4_ ring adopts a butterfly conformation (**1**, N1-N2-N4-N3 torsion angle=31.20(8)°; **4**, N1-N11-N31-N21 torsion angle=58.9(2)°). Despite the differing steric properties of the amido groups in **1** and **4**, the N-Cu-N bond angles are also similar (**1** 170.31(8)–173.09(8)°; **4** 171.68(14)–173.20(14)°). The Cu—N bond lengths in **4** lie in the range 1.890(4)–1.907(5) Å (mean 1.897 Å), whereas in **1** they are slightly elongated in comparison, range 1.902(2)–1.940(2) Å (mean 1.921 Å). In contrast to **1** and **4**, the central eight-membered Cu_4_N_4_ ring in **2** is planar, giving a tetramer of *C*_4*h*_ symmetry. The coordination of the copper(I) ions is close to linear in complex **2** with N-Cu-N bond angles of 178.46(5)° (N1-Cu1-N2A) and 178.23(5)° (N2-Cu2-N1). Cu-N-Cu bond angles are all 88.37(4)° and the Cu—N bond lengths are between 1.9326(11) Å and 1.9447(11) Å. The structure of **2** is therefore closely related to that previously reported for **3**,[46] which also adopts a close to planar Cu_4_N_4_ conformation with Cu-N-Cu bond angles of 91.0(2)° and 93.4(2)°, N-Cu-N bond angles of 175.2(2)° and 178.9(2)° and Cu—N distances of 1.9326(11)–1.9447(11) Å (mean 1.9389 Å).

**Table 1:** Selected bond lengths [Å] and angles [°] in complexes 1, 2 and 4.

**Complex 1**				
Cu1—N1	1.902(2)		N1-Cu1-N4	173.09(8)
Cu1—N4	1.902(2)		N2-Cu2-N1	171.85(9)
Cu2—N2	1.9347(19)		N2-Cu3-N3	172.62(8)
Cu2—N1	1.940(2)		N3-Cu4-N4	170.31(8)
Cu3—N2	1.9020(19)		Cu1-N1-Cu2	86.90(8)
Cu3—N3	1.9108(19)		Cu3-N2-Cu2	87.81(8)
Cu4—N3	1.9355(19)		Cu3-N3-Cu4	86.49(8)
Cu4—N4	1.939(2)		Cu1-N4-Cu4	86.83(8)
				
**Complex 2**				
Cu1—N1	1.9366(11)		N1-Cu1-N2A	178.46(5)
Cu1—N2A	1.9402(11)		N2-Cu2-N1	178.23(5)
Cu2—N2	1.9326(11)		Cu1-N1-Cu2	88.37(4)
Cu2—N1	1.9447(11)		Cu1A-N2-Cu2	88.38(5)
N2—Cu1A	1.9402(11)			
				
**Complex 4**				
Cu1—N1	1.894(4)		N1-Cu1-N4	172.87(18)
Cu1—N4	1.904(5)		N1-Cu2-N2	171.66(19)
Cu2—N2	1.891(5)		N2-Cu3-N3	173.18(19)
Cu2—N1	1.898(4)		N3-Cu4-N4	171.8(2)
Cu3—N3	1.893(4)		Cu1-N1-Cu2	87.8(1)
Cu3—N2	1.907(5)		Cu2-N2-Cu3	87.2(2)
Cu4—N4	1.890(4)		Cu3-N3-Cu4	87.5 (1)
Cu4—N3	1.902(4)		Cu4-N4-Cu1	87.3(2)

It is interesting to note that the Cu—N bond lengths within these copper(I) amide structures show a positive correlation with the steric size of the amide ligand, with C—N bond length increasing in the order **3**<**4**<**1**<**2** (Table 2). No similar relationship between bond angles and ligand steric size is observed.

**Table 2:** Comparison of mean Cu—N bond lengths, N-Cu-N and Cu-N-Cu bond angles in the solid-state tetrameric copper(I) amide structures.

Complex	Cu—N [Å]	N-Cu-N [°]	Cu-N-Cu [°]
**1**	1.921	171.97	87.01
**2**	1.939	178.35	88.38
**3[a]**	1.885	177.05	92.22
**4**	1.897	172.38	87.50

[a] Taken from ref. [46].

### Reaction of CuMes with piperidine

**Figure 2:**
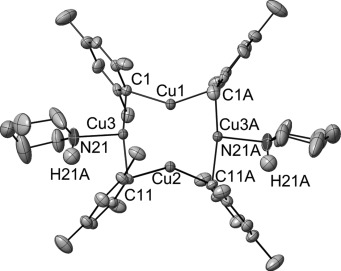
Structure of one of the two independent [Cu_4_(Mes)_4_(HNC_5_H_11_)_2_] molecules present in crystals of 6, determined by X-ray crystallography. Thermal ellipsoids are set at 50 % probability. Only hydrogen atoms present on the amine nitrogen are shown.

The reaction of the amines with CuMes (Route B, Scheme 3) was further studied in order to investigate why this direct route was not applicable to the bulkier TMP and dicyclohexylamine substrates. Thus treatment of piperidine with CuMes in THF gave initially a yellow crystalline precipitate, which subsequently dissolved on stirring to give a clear solution from which the desired product copper(I) piperidide (**4**) was then obtained. Isolation of the initial precipitate and analysis using ^1^H NMR spectroscopy revealed a 2:1 CuMes/piperidine ratio with the piperidine N—H proton still detected at *δ*=0.47 ppm. Furthermore, the ^1^H NMR piperidine resonances were shifted upfield compared to free piperidine suggesting coordination of the amine to a copper(I) centre (see Experimental Section for details). Yellow crystals of this intermediate that were suitable for study by X-ray crystallography were grown from the initial reaction mixture by allowing the solution to stand at room temperature for 12 h with no stirring. The crystals obtained were shown by single crystal X-ray diffraction to be the organocopper species [Cu_4_(Mes)_4_(HNC_5_H_11_)_2_] (**6**; Figure 2). The spectroscopic data of **6** are identical to those observed for the reaction precipitate, indicating that they are the same species. Two independent molecules of *C*_2_ symmetry are present in the crystal structure of **6**. These can be differentiated by the orientation of their *C*_2_ axes: In one (shown in Figure 2), the *C*_2_ axis passes through the short diagonal of the Cu_4_ rhombus, whereas in the other the *C*_2_ axis passes through the long diagonal of the Cu_4_ rhombus. The bond lengths and angles are approximately equivalent in both structures and hence only one of the independent molecules is presented below (Figure 2, Table 3).

**Table 3:** Selected bond lengths [Å] and angles [°] in complex 6.

Complex6				
Cu1—C1	2.029(6)		Cu1-C1-Cu3	73.64(19)
Cu1—C1A	2.029(6)		Cu2-C11-Cu3	73.74(18)
Cu2—C11	2.010(6)		C1-Cu1-C1A	143.3(3)
Cu2—C11A	2.010(6)		C1-Cu3-C11	170.2(2)
Cu3—C1	2.050(6)		C11A-Cu2-C11	142.0(3)
Cu3—C11	2.079(6)			
Cu3—N21	2.133(5)			

Structural characterisations of the organocopper species CuMes have revealed it to exist as either a tetramer or pentamer in the solid state, containing eight-membered Cu_4_C_4_ or ten membered Cu_5_C_5_ rings, respectively.[50] Moreover, the addition of sulfur-based ligands to CuMes has been shown to give complexes of general formula [Cu_4_Mes_4_L_2_] (L=tetrahydrothiophene,[51] allyl methyl sulfide or 2,5-dithiahexane[52]), based on eight-membered Cu_4_C_4_ rings with alternate copper centres coordinated by the neutral sulfur donor centres. The structure of **6** therefore conforms to a similar structural design [Cu_4_Mes_4_L_2_] where L now is HNC_5_H_11_. To our knowledge, this represents the first structurally characterised example of secondary amine coordination to an organocopper(I) centre. The nitrogen donors adopt tetrahedral conformations with a mean Cu—N distance of 2.140 Å (range 2.133(5)–2.155(5) Å), which is significantly longer than reported Cu—N distances in copper(I) amides such as the copper(I) piperidide complex **4** (1.890(4)–07(5) Å).

Similar to sulfur-ligated CuMes aggregates, slight puckering of the Cu_4_C_4_ ring is observed resulting in a central Cu_4_ rhomboidal arrangement. This contrasts to the planar square Cu_4_ arrangement observed in [Cu_4_Mes_4_].[50] The mean bond length in **6** for Cu—C bonds incorporating three coordinate copper(I) centres is 2.062 Å (range 2.050(6)–79(6) Å) and is therefore longer than the Cu—C bonds incorporating two coordinate copper(I) centres (mean 2.011 Å; range 1.997(7)–29(6) Å). In addition, these Cu—C distances in **6** are significantly longer than the corresponding Cu—C distances in the non-ligated tetramer [Cu_4_(Mes)_4_] (mean Cu—C distance 1.993 Å; range 1.986(10)–1.999(9) Å).[50]

The structure of **6** is consistent with it being an intermediate in the formation of **4**, in which the Cu_4_Mes_4_ aggregate is first coordinated by piperidine, before subsequent deprotonation of the amine by the mesityl group to give **4** and mesitylene. The larger steric bulk of dicyclohexylamine and 2,2,6,6-tetramethylpiperidine would likely prevent the formation of similar [Cu_4_Mes_4_L_2_] complexes due to steric crowding around the copper(I) centres. Indeed, attempts to prepare [Cu_4_Mes_4_L_2_] for L=TMP or HN(C_6_H_11_)_2_, from analogous reactions between CuMes and LH, were all unsuccessful, yielding just the original CuMes starting material in both cases. This reluctance to form [Cu_4_Mes_4_L_2_]-type aggregates with bulkier amines could account for the failure of Route B for copper(I) amides **1** and **2**.

### Study of solution behaviour of copper(I) amide complexes by ^1^H DOSY NMR

In addition to the solid-state structures, elucidating the solution structures and solution behaviour of the copper(I) amide complexes is crucial to building a better understanding of the role these species may play in the modified Ullmann reaction. To this end, the aggregate species that were present in solution were determined with the aid of ^1^H diffusion-ordered NMR spectroscopy (DOSY). This technique has recently been advanced for the characterisation of other organometallic aggregates.[53–58] The most commonly reported method involves obtaining empirical formula weights (FW) of unknown aggregates from their experimentally determined diffusion coefficients (*D*) and then comparing with FW of predicted species. However, although this method works well for organometallic species involving lighter metals (such as lithium[55]) which tend to possess consistent densities (ca. 1 g cm^−3^), the incorporation of the heavier copper element leads to a larger variation in density (X-ray crystallographically determined densities for **1**–**4** lie the range 1.324–1.659 g cm^−3^) and using this method we were unable to obtain internally self-consistent results for these compounds.

Instead, identification of structures in solution was determined by comparing the experimentally determined radii (*r*_obs_, obtained by correlating with observed *D* values[59]) with computed radii of DFT-optimised copper(I) amide structures (*r*_calc_; see the Supporting Information for full details). As *D* is also dependent on other factors, such as viscosity and temperature, internal standards (1,2,3,4-tetraphenylnaphthalene, 1-phenylnaphthalene and tetramethylsilane) were used to correct for these effects.

**Figure 3:**
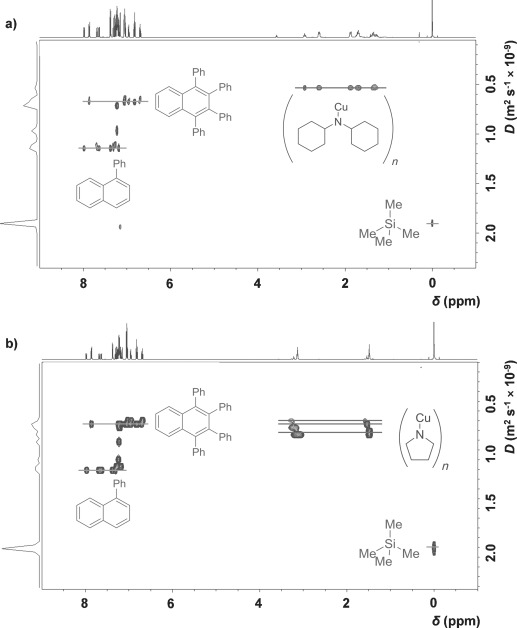
^1^H DOSY NMR spectra of a) complex 1 (0.05 m) and b) complex 3 (0.05 m) in [D_6_]benzene in the presence of 1,2,3,4-tetraphenylnaphthlene, 1-phenylnaphthalene, and tetramethylsilane as internal standards.

The proton resonances for the amide groups in complexes **1**, **2** and **5** each display only one diffusion coefficient (*D*=5.36×10^−10^, 5.17×10^−10^ and 5.17×10^−10^ m^2^ s^−1^ for **1**, **2** and **5**, respectively, at 0.05 m monomer concentration) in their corresponding ^1^H DOSY NMR spectra (for example, see Figure 3 a for complex **1**). This implies that either the equilibrium between aggregates is faster than the NMR time-scale at room temperature or that there is only one aggregation state present in solution. Increasing the concentration of **1**, **2** and **5** led to a decrease in the diffusion coefficients (increases in *r*_obs_), suggesting the former of these assumptions (fast solution equilibrium between aggregates) to be the case. In contrast, the ^1^H DOSY NMR spectra of complexes **3** and **4** both show three distinct sets of resonances for the amido group protons, each with differing *D* values corresponding to three different *r*_obs_ values (for example, see Figure 3 b for complex **3**). This is indicative of three different distinct aggregation states being present in solution with any equilibrium between aggregates occurring on a much slower timescale than that observed for **1**, **2** and **5**.

The ^1^H DOSY NMR results for complexes **1**, **2** and **5**, which all show rapid solution equilibria, are presented in Table 4. The *r*_obs_ values of 6.28 and 5.77 Å for complexes **1** and **2**, respectively, at 0.05 m concentration indicate that these species likely exist in equilibrium predominantly between dimeric (*n*=2) and trimeric (*n*=3) aggregation states, with predicted average aggregation numbers of 2.5 for **1** and 2.4 for **2** (Table 4). A *r*_obs_ value of 5.88 Å for complex **5** at 0.05 m concentration corresponds to *n*=3.5, suggestive of an equilibrium predominantly between trimeric and tetrameric states. Varying the concentration of the copper(I) amide led to increasing *r*_obs_ values with increasing concentration, although the range of these experiments were somewhat limited by the generally poor solubility of the copper(I) amides in benzene at the higher end and the resolution of the NMR spectrometer at the lower end.

**Table 4:** Comparison of calculated and experimentally determined radii of copper(I) amide aggregates in [D_6_]benzene solutions determined using ^1^H DOSY NMR.

Complex	Conc. [m]	*r*_obs_ [Å]	Aggregation number *n*	Predominant equilibria inferred from *r*_obs_
	0.05	6.28	2.6	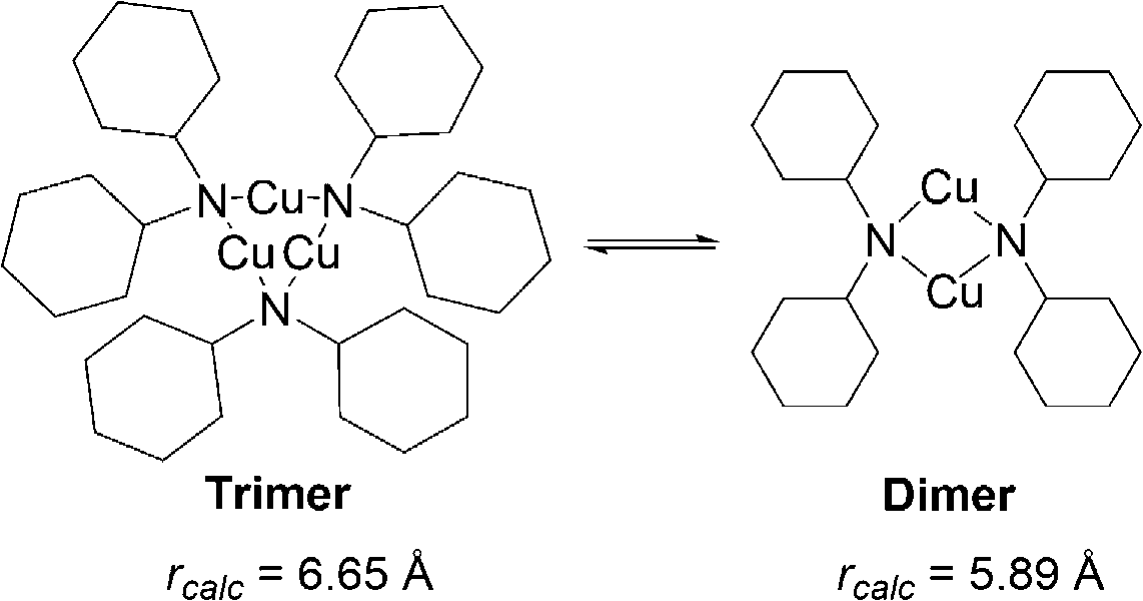
**1**				
			
	0.15	6.31	2.6	
				
				
				
	0.05	5.57	2.4	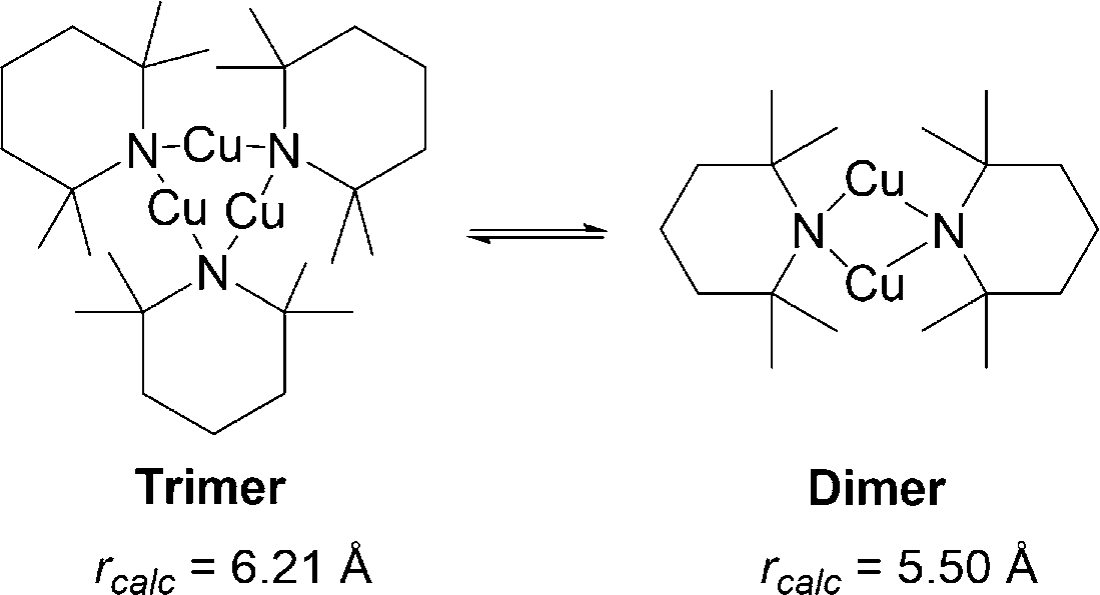
**2**				
			
	0.10	5.59	2.4	
				
				
				
	0.01	5.78	3.3	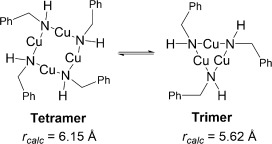
**5**				
			
	0.05	5.88	3.5	
				

The DOSY NMR results for complexes **3** and **4**, which both underwent slower solution equilibrium, are shown in Table 5. The *r*_obs_ values were all consistent with the *r*_calc_ values within ±6 % (this is well within the error margins previously reported for DOSY aggregation experiments[53]).

**Table 5:** Comparison of calculated and observed radii of copper(I) amide aggregates in [D_6_]benzene solutions determined using ^1^H DOSY NMR. Percentage differences from expected *r*_calc_ values were calculated for *r*_obs_ values. Three *r*_obs_ values are given as there were three distinct sets of resonances with different diffusion coefficients for complexes 3 and 4.

Complex	*r*_calc_ [Å]		*r*_obs_ at 0.05 m [Å]	*r*_obs_ at 0.20 m [Å]	
	Pentamer	6.04		5.88 (−2.7 %)	5.80 (−4.0 %)	
**3**	Tetramer	5.65		5.65 (+0.1 %)	5.68 (+0.7 %)
	Trimer	5.15		5.45 (+6.0 %)	5.38 (+4.4 %)
						
	Pentamer	6.37		6.16 (−3.3 %)	6.16 (−3.4 %)	
**4**	Tetramer	5.89		5.89 (+0.0 %)	5.89 (+0.0 %)
	Trimer	5.38		5.55 (+3.2 %)	5.65 (+5.2 %)

The relative abundance of the different aggregate species in the solutions of **3** and **4** could be determined from the integration of the peaks in the ^1^H NMR spectrum, and from this it was possible to obtain an estimated mean aggregation state. At a concentration of 0.05 m, the mean aggregations states for **3** and **4** were 3.2 and 3.1 respectively. When the concentration was increased to 0.20 m, the same three aggregates (same *r*_obs_) were still detected for each complex. However, the integrations of the tetramer and pentamer NMR resonances increased relative to that of the trimer resonances. Thus, for example, the ratio of trimer/tetramer/pentamer integrations in **3** changed from 1.00:0.25:0.07 at 0.05 m to 1.00:0.31:0.11 at 0.20 m.[60] As a result, the average aggregation number of **3** increased from 3.2 to 3.3 and that of **4** from 3.1 to 3.3.

**Figure 4:**
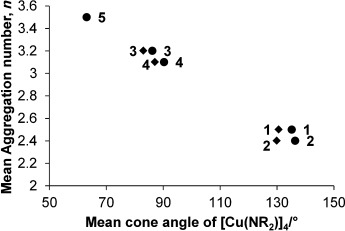
Plot of aggregation numbers in [D_6_]benzene solution at 0.05 m concentration against measured cone angles of the solid-state and DFT optimised tetrameric copper(I) amide structures. Circles: solid state; diamonds: DFT.

A possible reason for the copper amide complexes **3** and **4** being observable as distinct aggregates in [D_6_]benzene solution, whereas **1**, **2** and **5** show more rapid equilibrium rates, could be the shorter, stronger Cu—N bonds in **3** and **4** (see Table 2) leading to slower exchange between aggregates. In addition, the aggregation numbers of the complexes can also be related to the mean cone angles of the ligands, where the cone angle is defined similarly to the method developed by Tolman,[61] with the copper(I) ion at the vertex and the perimeter of the cone passing through the centre of the outermost hydrogen atoms. Based upon the tetrameric solid-state structures obtained through X-ray crystallography (see above) and also the DFT-optimised structures (see Experimental Section for details), the cone angles were measured and are reported in Table 6. There is an inverse relationship between increasing cone angle size and solution aggregation number (Figure 4). Thus the more sterically bulky amides TMP and NCy_2_ possess larger cone angles and adopt lower average aggregation states, whereas the least sterically bulky N(H)CH_2_Ph amide has the smallest cone angle and the largest mean aggregation state.

**Table 6:** Comparison of cone angles of the ligands in the tetrameric copper(I) amide structures as determined from solid-state structures and also measured from DFT optimised structures with the aggregation numbers determined in [D_6_]benzene solution at 0.05 m concentration.

Complex	Mean cone angle of [Cu(NR_2_)]_4_ from solid state [°]	Mean cone angle of DFT optimised [Cu(NR_2_)]_4_ [°]	Mean Aggrega- tion number *n*
**1**	130.6	135.2	2.5
**2**	129.9	136.4	2.4
**3**	83.0[a]	86.2	3.2
**4**	87.1	90.3	3.1
**5**	N/A	63.1	3.5

[a] Taken from ref. [46].

These studies therefore show that although all of the copper(I) amide complexes studied are present as tetramers in the solid state, in solution they adopt a number of aggregation states with average aggregation numbers of 3.0±0.5 in [D_6_]benzene. The steric bulk of the amide group was shown to be inversely related to the average aggregation number. The different aggregates exist in equilibrium in solution, with the rate of equilibrium thought to be dependent upon the Cu—N bond strength with shorter, stronger bonds (as observed in the solid-state structures) leading to lower rates. Although analogous studies were also attempted in [D_6_]DMSO, all copper(I) amides studied were found to be of too low solubility for similar NMR studies to be undertaken.

### Reactivity of copper(I) amides in aryl amination

Copper(I) amide complexes **1**–**5** were treated with iodobenzene at 80 °C in both [D_6_]benzene and [D_6_]DMSO to investigate their reactivity in the Ullmann aryl amination reaction (Table 7) and to ascertain their competence as intermediaries on the catalytic cycle (Scheme 2).

**Table 7:** Yields obtained in reactions between the copper(I) amide complexes and iodobenzene in [D_6_]benzene and [D_6_]DMSO solvent. Yields were determined by NMR using mesitylene as an internal standard and are reported as a mean of at least two independent runs.

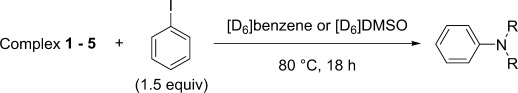		
Complex	Yield [%]	
	[D_6_]benzene solvent	[D_6_]DMSO solvent
**1**	0	0
**2**	0	0
**3**	48	75
**4**	96	87
**5**	7	77

Reactions involving the bulky copper(I) amides **1** and **2** failed to produce any product in either solvent. This lack of reactivity is most likely due to steric hindrance. Copper(I) amide **3** is significantly less bulky than **1** and **2** and gave higher yields in the coupling reaction in both solvent systems. However, **3** was also sensitive to thermal decomposition at room temperature or above, to give the β-hydride elimination product, 1-pyrroline, as a side-product in 10 % yield in [D_6_]benzene and 6 % yield in [D_6_]DMSO. Copper(I) amide **4** displayed better thermal stability in solution and as a result underwent almost quantitative conversion (96 % yield) to the arylamine product in [D_6_]benzene with no observable side-products. In [D_6_]DMSO, the reaction of copper(I) amide **4** with iodobenzene gave the arylamine product in 87 % yield with some piperidine side-product also formed (8 % yield). Despite being the least sterically hindered of the studied complexes, the reaction with copper(I) amide **5** in [D_6_]benzene gave only 7 % yield of the desired product. Further experiments show that [D_6_]benzene solutions of **5** rapidly undergo decomposition to give benzylamine and an unknown insoluble precipitate; after standing at room temperature for one hour, only 68 % of **5** remained with 5 % benzylamine also present, and after one hour at 80 °C only 12 % of **5** remained with 24 % benzylamine present. However, on changing the solvent to [D_6_]DMSO, the coupling yield using **5** was significantly improved to 77 %, with only small amounts of benzylamine detected (6 %).

In both solvents, the mass balance between the product yield and iodobenzene conversion was generally quite good, differing by no more than 7 %. The solubility of the copper(I) amide complexes in [D_6_]DMSO is very poor (see above) and therefore it is difficult to ascertain whether the reactions using this solvent occurred heterogeneously or homogeneously. The formation of amine byproducts in the reactions involving **3**–**5** could have resulted from abstraction of a proton from adventitious water or a solvent molecule, as previously reported by Hartwig[30] and Ribas[62].

Catalytic reactions between the corresponding parent amines of complexes **1**–**5** and iodobenzene were also performed under the same conditions but with 10 mol % copper(I) iodide and two equivalents of K_2_CO_3_ base. No catalytic activity was observed when [D_6_]benzene was used as the solvent in all cases. This is perhaps not surprising, given the insolubility of both the base and the copper(I) iodide in this solvent. Literature protocols typically employed DMSO[20, 24, 63–66] or DMF[23, 25, 67–69] as the solvent to aid with mass transfer of the base and catalyst. In our hands, [D_6_]DMSO proved a suitable solvent for the coupling reactions, allowing complete dissolution of the copper(I) iodide (Table 8).

**Table 8:** Yields obtained in the modified Ullmann amination reactions with iodobenzene. Yields were determined by NMR using mesitylene as an internal standard. Yields are reported as a mean of at least two independent runs.

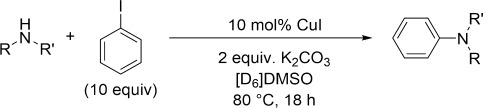		
Entry	HNRR′	Yield [%]
1	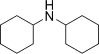	0
2	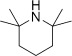	0
3	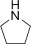	68
4	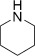	51
5	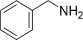	89

When the sterically hindered TMP and dicyclohexylamine substrates were employed, no coupling product was formed (Table 8, entries 1 and 2). This is analogous to the observations from the stoichiometric reactions using **1** and **2**. The other three amines did demonstrate some reactivity with the best conversion (89 %) found using the least-hindered primary benzylamine (Table 8, entry 5). The cyclic secondary amines, pyrrolidine and piperidine, gave moderate yields of 68 % and 51 % respectively (Table 8, entries 3 and 4). Hence, similar or marginally higher reaction yields were obtained using the isolated copper(I) amide complexes as stoichiometric reagents when compared to their catalytic counterparts. This is consistent with the copper(I) amide complexes being competent reaction intermediates in the modified Ullmann amination reaction and also represents the first experimental evidence of copper(I) amide aggregates reacting with aryl halides in the absence of any ancillary ligands. The results also shed light on the role of steric factors in the reaction. Although the steric bulk of the amine is a known issue in the catalytic modified Ullmann amination reaction,[20, 70] the lack of reactivity for **1** and **2** provides the first experimental evidence supporting that this is likely to be associated with inhibition of aryl halide oxidative addition rather than impeded copper(I) amide formation.

### Identification of solution structures of complexes 3 and 4 in the presence of 1,10-phenanthroline

A number of different ligand systems have been reported for accelerating the modified Ullmann reaction, with perhaps one of the most studied being 1,10-phenanthroline (phen). Phen has been shown to significantly improve the yields and reaction rates in the arylation of arylamines,[4–9] hydrazines[71] and aliphatic alcohols.[72, 73] However, studies employing this ligand in alkylamine-aryl coupling reactions are less well documented, with diketone and amino acid based ligands more often employed.[20, 63, 68, 74] To understand the potential influence and role of the phen ligand in catalytic coupling reactions of alkylamines with aryl halides, the interactions of phen with the copper(I) amides prepared in this work were investigated.

Given that copper(I) amides **3** and **4** demonstrated the best reactivity with iodobenzene (Table 7) and are also relatively stable in solution, the interaction of these complexes with the phen ligand were first studied. In both cases, it was not possible to isolate any phen-ligated species on addition of phen to a solution of the copper(I) amide, and studies therefore focused upon elucidating the solution behaviour of the copper(I) amides upon addition of phen.

**Scheme 4:**
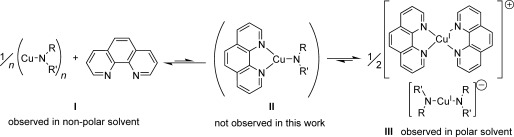
General reaction scheme showing the equilibrium between the neutral catalyst resting state and an ionic species when phen is used as the ancillary ligand with 3 or 4.

**Scheme 5:**
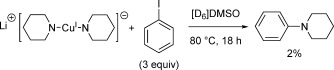
Reaction between lithium bis(piperidido)cuprate(I) and iodobenzene. Yields were determined by NMR spectroscopy using mesitylene as an internal standard. Yields are reported as a mean of at least two independent runs.

When one equivalent of phen was added to one equivalent of **3** or **4** in [D_6_]benzene the initial colourless solution of copper(I) amide changed to a deep blue colour. This is suggestive of phen bound to a copper(I) centre.[34] However, despite this in the ^1^H NMR spectra the proton resonances for the aggregates of the copper(I) amides remained unchanged and the phen resonances were shifted downfield by less than 0.05 ppm when compared to free phen. In addition, ^1^H DOSY NMR analyses gave observed radii that were consistent with the expected aggregates of ligand-free copper(I) amide and of uncoordinated phen (*r*_obs_=4.51 Å cf. *r*_calc_=4.40 Å). This indicates that the equilibrium for the binding of phen lies almost exclusively on the non-ligated side comprising the copper(I) amide aggregate and free phen (**I**, Scheme 4) with no NMR spectroscopic evidence for any ligated species in solution. Ionic species of general formula [CuL_2_]^+^[Cu(NR_2_)_2_]^−^ (**III**, L=phen, Scheme 4) have also been identified in similar systems where they have been proposed to exist in equilibrium with mono-ligated neutral [LCu(NR_2_)] complexes (**II**, L=phen, Scheme 4),[5, 6, 29, 30] however such ionic species are also not observed in the ^1^H NMR spectrum for either of these mixtures in benzene solution. Furthermore, the solutions formed were unstable over time (more so than their parent copper(I) amide solutions in the absence of phen). Monitoring by ^1^H NMR spectroscopy at room temperature shows that after two hours of addition of phen only 81 % of the original copper(I) amide remained and after 18 h, just 46 % copper(I) amide remained with Cu^0^ also now present. In comparison, the amounts of **3** and **4** remain virtually unchanged after similar storage for 19 h.

**Figure 5:**
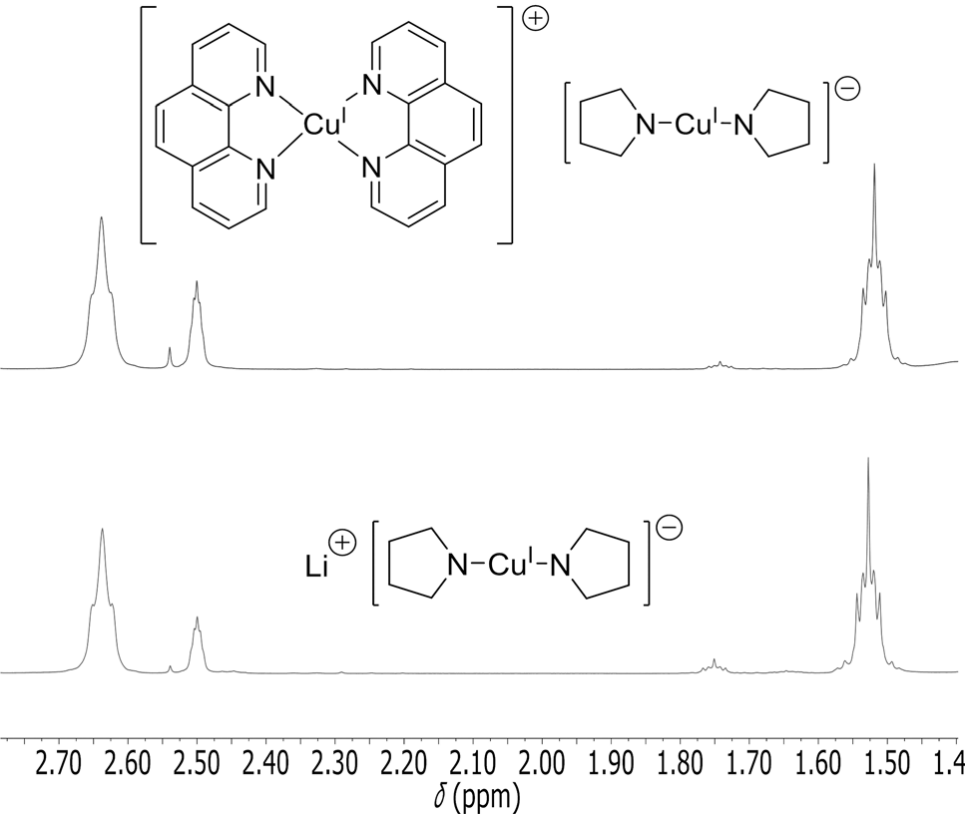
Cropped ^1^H NMR spectrum of 3 with phen (1:1 molar ratio; top) in [D_6_]DMSO demonstrating the similar chemical shifts of the pyrrolidide ligand resonances with Li[Cu(pyrrolidide)_2_] (bottom) at *δ*=1.52 ppm and 2.64 ppm for the β and α protons, respectively. Residual [D_5_]DMSO at *δ*=2.50 ppm.

Although copper(I) amides **3** and **4** are themselves virtually insoluble in [D_6_]DMSO solvent, on addition of one equivalent of phen complete dissolution of the complexes occurred to give a deep-red coloured solution. In the ^1^H NMR spectra, one set of phen resonances were observed which were significantly shifted from those of free phen, suggestive of coordination of the phen to a copper(I) centre either in a neutral [LCu(NR_2_)] or a ionic [CuL_2_]^+^[Cu(NR_2_)_2_]^−^ species (Scheme 4). In order to ascertain which of these was most likely the lithium bisamidocuprate Li[Cu(NC_4_H_8_)_2_] was prepared from the reaction of two equivalents of lithium pyrrolidide with CuI. Direct comparison of the ^1^H NMR between Li[Cu(NC_4_H_8_)_2_] and the solution of **3**+phen revealed almost identical shifts for the pyrrolidide protons, suggesting the presence of the same bisamidocuprate [Cu(NC_4_H_8_)_2_]^−^ anion in both cases (Figure 5). In addition, the ^1^H–^1^H ROESY NMR spectrum for **3**+phen showed no positive-phase cross peaks between the amide and phen ligands’ resonances (see the Supporting Information), which also supports the major species in solution being the ionic form with the phen and amido groups each attached to different metal centres.[75]

ESI-MS spectra of complex **4** with phen in both dimethyl sulfoxide and benzene produced similar spectra in positive and negative mode with the cationic [Cu(phen)_2_]^+^ and anionic [Cu(piperidide)_2_]^−^ species detected in the positive and negative spectra at 423.0 and 230.8 *m*/*z*, respectively.[76] This suggested that the ionic form was present and is in agreement with the NMR data in [D_6_]DMSO. In addition, the amidocuprate corresponding to the higher aggregate [Cu_4_(piperidide)_5_]^−^ (FW=674.89) could also be present in the solution as there was a set of ions around 674.5 *m*/*z*. Similar anionic cuprate clusters including [Cu_4_(μ-SCH_2_Ph)_6_]^2−^ and [Cu_5_Ph_6_]^−^ have been reported as intermediaries in closely related copper-catalysed C—S bond forming reactions and also in studies relating to Gilman cuprate reagents.[77, 78]

In summary, for copper(I) amide/phen mixtures in DMSO, the solution equilibrium is shown to lie almost exclusively on the side of the ion pair species. This is congruent with previous studies, in particular Hartwig’s work on copper(I) diarylamides in this solvent.[30] However in benzene the coordination of phen with the copper(I) amide aggregates is disfavoured with the equilibrium lying predominantly on the side of ‘ligand-free’ copper(I) amide aggregates and free phen. To our knowledge, this is the first time that ligand-free copper(I) amides have been directly observed in mixtures of copper(I) amides and ancillary ligands. When combined with the newly reported high reactivity of ‘non-ligated’ copper(I) amide aggregates (see above), this has implications on our understanding of the reactivity and selectivity in the modified Ullmann reaction and suggests that any comprehensive reaction scheme or model should also consider the potential role of these ligand-free aggregates in the catalytic process.

### Stoichiometric and catalytic reactivity in the presence of 1,10-phenanthroline

The yields of coupling product obtained from the stoichiometric reactions of the copper(I) amide complexes with iodobenzene on addition of one equivalent of phen in [D_6_]benzene and [D_6_]DMSO are shown in Table 9.

**Table 9:** Yields obtained in reactions between the copper(I) amide complexes and iodobenzene in [D_6_]benzene and [D_6_]DMSO solvent in the presence of phen. Yields were determined by NMR using mesitylene as an internal standard. Yields are reported as a mean of at least two independent runs.

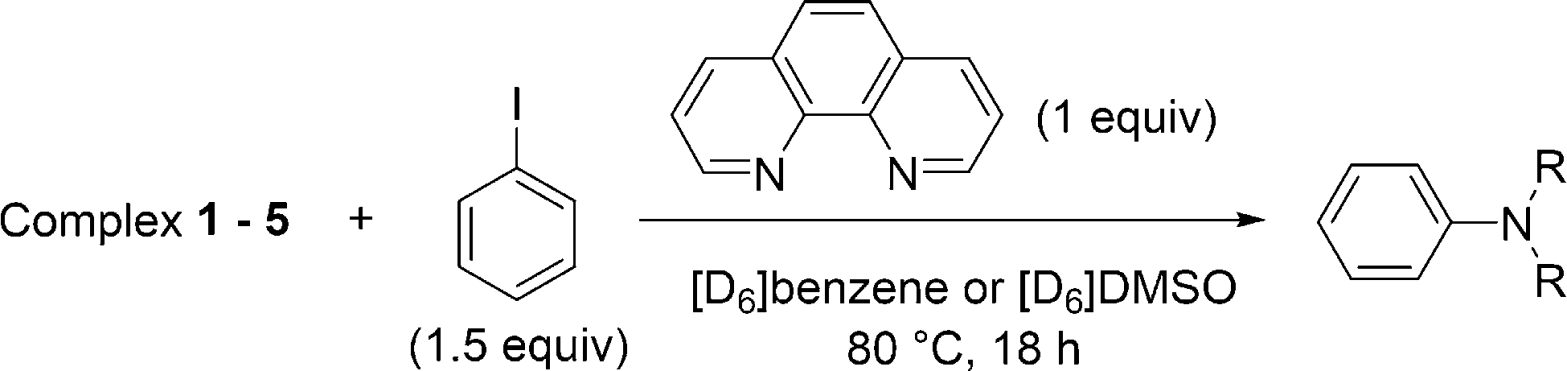		
Complex	Yield [%]	
	[D_6_]benzene solvent	[D_6_]DMSO solvent
**1**	0	0
**2**	0	0
**3**	44	26
**4**	51	11
**5**	9	66

Reactions involving the more sterically hindered copper(I) amides, **1** and **2**, produced no product with phen present, which is the same as the ligand-free experiments and again can be attributed to steric hindrance. The coupling yields in [D_6_]benzene using complexes **3** or **5** as reagents were not greatly affected by the addition of phen giving similar yields to the ligand-free experiments. However the yield of cross-coupled product formed from complex **4** in [D_6_]benzene decreased from 96 to 51 % on addition of phen. The yields on addition of phen in [D_6_]benzene are therefore all similar or reduced compared to the ligand-free systems (Table 6). The solution spectroscopic experiments reported above showed non-phen-bound copper(I) amide aggregates to be by far the predominant species in these solutions and it is these aggregates that are likely to constitute the reactive species, both in the presence and absence of phen. The lower reactivity of **4**+phen compared to phen-free **4** can be attributed to the lower stability of the copper(I) amide on addition of phen (see above).

The effect of phen on the reactivity of **3** was more pronounced when [D_6_]DMSO was used as the solvent, with the yield unexpectedly decreasing from 75 % to 26 %. The yields obtained with complexes **4** and **5** on addition of phen were also diminished in [D_6_]DMSO compared to their ligand-free analogues. These results are somewhat surprising in light of the prominent role played by phen in copper-catalysed Ullmann amination.[4–9] Nevertheless, they can be explained in the context of the solution studies which showed the favoured formation of the ionic cuprate species (**III**, Scheme 4) on addition of phen to copper(I) amide in DMSO. Previous studies on the reactivity of isolated bisamidocuprate complexes [Cu(NR_2_)_2_]^−^ (NR_2_=NPh_2_;[30] phthalimidate[29]) have shown these anionic species to have little or no reactivity in the cross-coupling reaction with aryl halides. If, as seems likely, the bis(dialkylamino)cuprate complexes present in the reaction mixture here also exhibit low reactivity, this could help explain the observed reduction in product yield. To verify this theory, lithium bis(piperidido)cuprate(I) was prepared (by the reaction of CuI and two equivalents of lithium piperidide) and then treated with iodobenzene (Scheme 5). Conversions obtained from this reaction were very low (<2 %), thus confirming bis(dialkylamido)cuprates to also be relatively inert in the C—N coupling reaction.

**Figure 6:**
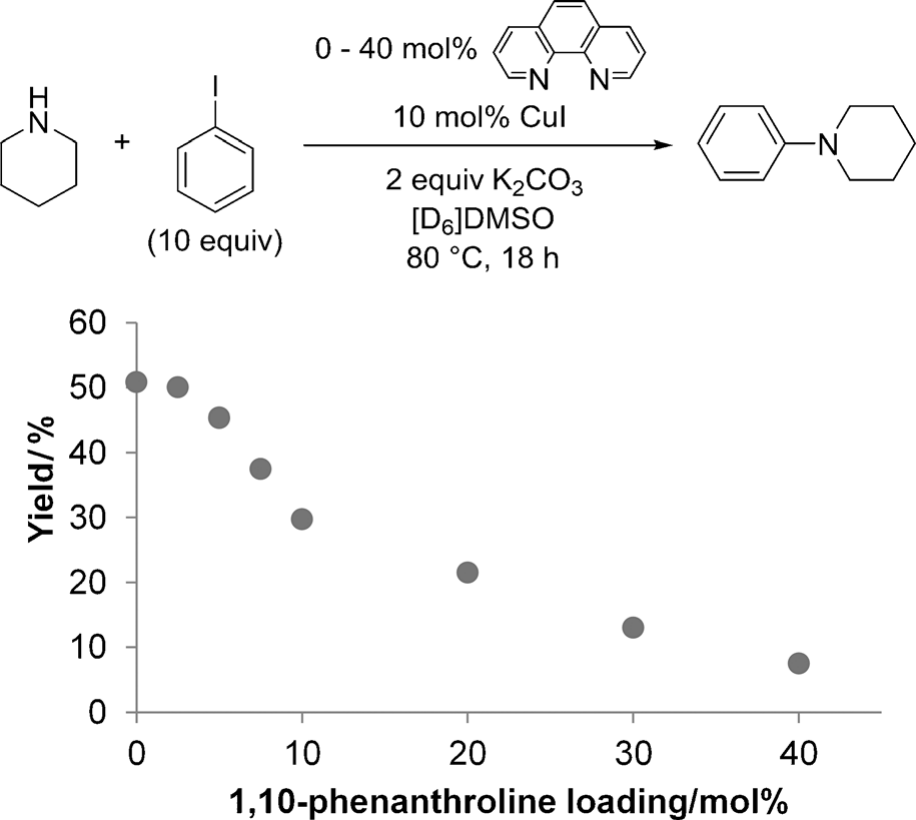
Chart showing the effect of phen on the yield of the C—N coupling between piperidine and iodobenzene. Yields were determined by NMR using mesitylene as an internal standard. Yields are reported as a mean of at least two independent runs.

Catalytic reactions were performed in the presence of 10 mol % CuI and 10 mol % phen in [D_6_]DMSO (Table 10) and also [D_6_]benzene, although all yields were 0 % in the latter case, which again can be attributed to the poor solubility of CuI and the base in this solvent (see above). In [D_6_]DMSO the sterically hindered amines, dicyclohexylamine and 2,2,6,6-tetramethylpiperidine, still did not produce any C—N coupling product with iodobenzene (Table 10, entries 1 and 2). However, the yields in the coupling reactions between pyrrolidine, piperidine and benzylamine with iodobenzene are decreased (relative to the phen-free systems) to 39 %, 30 % and 77 % respectively (Table 10, entries 3–5). To further investigate the influence of phen in dialkylamine systems, the coupling yield in the catalytic reaction between piperidine and excess iodobenzene was investigated over a range of phen loadings (Figure 6).

**Table 10:** Yields obtained in the modified Ullmann amination reactions in the presence of 10 mol % phen. Yields were determined by NMR using mesitylene as an internal standard. Yields are reported as a mean of at least two independent runs.

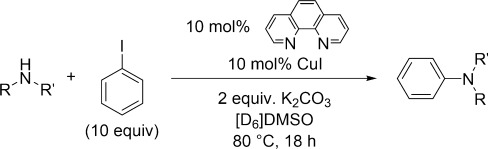		
Entry	HNRR′	Yield [%]
1	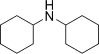	0
2	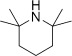	0
3	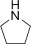	39
4	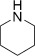	30
5	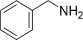	77

It is apparent from these results that increased loading of phen leads to a significant reduction in yield, with the highest yield observed in the ligand-free system. The addition of more phen is likely to further shift the solution equilibrium away from the reactive ligand-free copper(I) amide (or mono-ligated neutral [(phen)CuNR_2_] species) towards the unreactive ionic bisamidocuprate species.

Although these results may at first appear contrary to many previous studies, which favoured the use of phen-based ligands in Ullmann-type amination reactions, it should be noted that the position of the equilibrium and thus also the reactivity are likely to be very dependent upon a number of different factors. These include, but are not limited to, the p*K*_a_ of the amine, the solvent system and the reaction temperature. Direct comparison to the literature is therefore difficult and is compounded by the fact that conversions using the baseline ligand-free copper(I) catalyst are often not reported and phen ligand loadings are usually restricted to just one or in some cases two values. Our results though are compatible with most contemporary mechanistic models in which bisamidocuprates are off-cycle from the catalytic cycle and therefore unreactive towards aryl iodides.[27–30] They are also in agreement with recent computational studies, which have shown the barrier for oxidative addition of iodobenzene to ligand-free [(dmso)Cu(NPh_2_)] to be significantly lower than that for [(phen)Cu(NPh_2_)].[30]

## Conclusion

A series of copper(I) alkylamide complexes of differing steric bulk were synthesised and subsequently characterised in detail by X-ray crystallography and NMR techniques. Crystallographic data showed **1**, **2** and **4** to exist in their tetrameric form in the solid state. By using experimentally determined diffusion coefficients of **1**–**5** in [D_6_]benzene derived from ^1^H DOSY NMR studies, the behaviour of each of these complexes towards aggregation in solution was explored. Copper(I) amides **1**, **2** and **5** were shown to undergo rapid equilibrium on the NMR timescale whereas the trimeric, tetrameric and pentameric forms of **3** and **4** were distinguishable by NMR. The strength of the Cu—N bonds within the aggregate is thought to play a key role in determining the rate of aggregate exchange. The mean aggregation in solution for all compounds was determined and shown to form a strong inverse correlation with the steric bulk of the amide group.

Moreover, studies on the reactivities of **1**–**5** with iodobenzene, both in the presence and absence of phen, have revealed some new insights into the reactivity and solution behaviour of copper(I) amides with direct relevance to the catalytic mechanism of the modified Ullmann reaction. Firstly, the isolated copper(I) amides were shown to be competent intermediaries in the modified Ullmann amination reaction by comparing the similar stoichiometric and catalytic reactivity both with and without phen as an ancillary ligand. Most notably, in the absence of phen this represents the first report of a ligand-free copper(I) amide complex reacting with an aryl halide to yield the C—N-coupled product. Secondly, steric bulk was shown to significantly lower the reactivity of the copper(I) amide, thus providing some of the first experimental evidence that poor yields in catalytic reactions with bulky amines are likely inhibited at the aryl halide oxidative addition step rather than in formation of the copper(I) amide catalyst resting state. Thirdly the solution behaviour of copper(I) amide/phen mixtures is revealed to be more nuanced than often portrayed, with ‘ligand-free’ copper(I) amide aggregates shown (using ^1^H DOSY NMR experiments) for the first time to be present in appreciable quantities in non-polar solvents. Taken together with the newly reported reactivity of these ligand-free copper(I) amide aggregates, this suggests that any comprehensive reaction scheme or modelling should also consider the potential role of these ligand-free species in the catalytic process. Finally, studies on phen loading in the copper(I)-catalysed coupling reaction of piperidine and iodobenzene in DMSO surprisingly revealed that the ligand-free reaction gave the best yield, with yields dropping off with increasing phen loading. This can be attributed to the formation of the unreactive ion pair [Cu(NR_2_)_2_]^−^ [(phen)_2_Cu]^+^ in DMSO when phen is present in excess. At present, it is difficult to extrapolate these results beyond the dialkylamine substrates studied here, although further studies are underway to reveal how generally applicable these findings might be to other amine, amide and alcohol-based substrates.

## Experimental Section

**General information**: All manipulations were carried out under a nitrogen atmosphere in a glovebox or using standard Schlenk techniques. All solvents and reagents were purified and dried thoroughly prior to use (see the Supporting Information). Copper(I) mesityl was prepared according to a reported procedure by Tsuda et al.[42]

^1^H and ^13^C NMR spectroscopy data were obtained at room temperature by using Bruker AV-400 spectrometers, except for ^1^H DOSY and ^1^H–^1^H ROESY NMR, which were recorded on a Bruker AV-500. ESI-MS mass spectra were acquired on a Waters LCT Premier. X-ray crystallography data were collected using Oxford Diffraction Xcalibur 3 (**1** and **2**), Oxford Diffraction Xcalibur PX Ultra (**4**), and Agilent Xcalibur 3 E (**6**) diffractometers, and the structures were refined using the SHELXTL, SHELX-97, and SHELX-2013 program systems.[79–81]

CCDC-1027216 http://www.ccdc.cam.ac.uk/cgi-bin/catreq.cgi(**1**), CCDC-1027217 http://www.ccdc.cam.ac.uk/cgi-bin/catreq.cgi(**2**), CCDC-1027218 http://www.ccdc.cam.ac.uk/cgi-bin/catreq.cgi(**4**) and CCDC-1027219 http://www.ccdc.cam.ac.uk/cgi-bin/catreq.cgi(**6**) contain the supplementary crystallographic data for this paper. These data can be obtained free of charge from The Cambridge Crystallographic Data Centre via http://www.ccdc.cam.ac.uk/data_request/cif.

The yields reported for the syntheses of the copper(I) amide complexes were calculated from the amount isolated after purification. The quantities of each compound present after the catalytic and stoichiometric reactions were calculated from the NMR spectra, through the use of mesitylene as an internal standard, and are reported as a mean of at least two independent runs.

**Preparation of copper(I) dicyclohexylamide (1)**: A solution of dicyclohexylamine (0.80 mL, 4.00 mmol) in tetrahydrofuran (10 mL) was treated dropwise with *n*-butyllithium in hexanes (2.50 mL, 4.00 mmol, 1.6 m) at 0 °C. After stirring at 0 °C for 5 min, the solution was transferred dropwise to a suspension of copper(I) chloride (436 mg, 4.40 mmol) in tetrahydrofuran (5 mL) at 0 °C. After complete addition, the reaction mixture was allowed to come to room temperature and was stirred for 2 h. The mixture was then filtered through Celite, and then the filtrate was concentrated under reduced pressure to approximately 8 mL and then stored at −25 °C. After 3 days, the crystallised solid was isolated by filtration and then dried under vacuum to afford colourless crystals of the desired product (152 mg, 0.62 mmol, 16 %). ^1^H NMR (400 MHz, [D_6_]benzene, 22 °C, TMS): *δ*=1.20–1.45 (m, 6 H; CH_2_), 1.63–1.78 (m, 6 H; CH_2_), 1.82–1.93 (m, 4 H; β-CH_2_), 2.54–2.65 (m, 4 H; β-CH_2_), 2.92 ppm (tt, ^3^*J*(H,H)=11.0 Hz, ^3^*J*(H,H)=3.6 Hz, 2 H; α-CH); ^13^C NMR (101 MHz, [D_6_]benzene, 22 °C, TMS): *δ*=27.3 (s; γ-CH_2_), 27.6 (s; δ-CH_2_), 40.5 (s; β-CH_2_), 60.5 ppm (s; α-CH); elemental analysis calcd (%) for C_12_H_22_CuN: C 59.10, H 9.09, N 5.74; found: C 59.17, H 9.22, N 5.60. Colourless crystals suitable for X-ray diffraction were obtained by allowing the compound to crystallise slowly from the filtrate mentioned above at 4 °C over 3 days.

**Preparation of copper(I) 2,2,6,6-tetramethylpiperidide (2)**: A solution of 2,2,6,6-tetramethylpiperidine (3.40 mL, 20.00 mmol) in tetrahydrofuran (10 mL) was treated dropwise with *n*-butyllithium in hexanes (12.50 mL, 20.00 mmol, 1.6 m) at 0 °C. After stirring at 0 °C for 5 min, the solution was transferred dropwise to a suspension of copper(I) chloride (2.178 g, 22.00 mmol) in tetrahydrofuran (65 mL) at 0 °C. After complete addition, the reaction mixture was allowed to come to room temperature and was stirred for 40 min, after which it was then filtered through Celite. The filtrate was then concentrated under reduced pressure and then kept at −25 °C. After 5 days, crystallised solid was isolated by filtration and then dried under vacuum to afford the desired product as a white solid (1.751 g, 8.59 mmol, 43 %). ^1^H NMR (400 MHz, [D_6_]benzene, 22 °C, TMS): *δ*=1.54–1.60 (m, 4 H; γ-CH_2_), 1.69–1.81 ppm (m, 14 H; CH_3_ and β-CH_2_); ^13^C NMR (101 MHz, [D_6_]benzene, 22 °C, TMS): *δ*=20.1 (s; γ-CH_2_), 38.0 (s; CH_3_), 43.3 (s; β-CH_2_), 57.7 ppm (s; α-C); elemental analysis calcd (%) for C_9_H_18_CuN: C 53.04, H 8.90, N 6.87; found: C 52.83, H 9.08, N 6.85. Colourless crystals suitable for X-ray diffraction were obtained by allowing the compound to crystallise slowly from the filtrate mentioned above at 4 °C over 4 days.

**Preparation of copper(I) pyrrolidide (3)**: A solution of pyrrolidine (1.11 mL, 13.50 mmol) in tetrahydrofuran (10 mL) was treated dropwise with *n*-butyllithium in hexanes (5.20 mL, 13.00 mmol, 2.5 m) at room temperature to give a colourless solution. After stirring for 10 min, the solution was transferred dropwise to a suspension of copper(I) chloride (1.39 g, 14.00 mmol) in tetrahydrofuran (50 mL) at room temperature. After complete addition, the reaction mixture was stirred for 25 min and then filtered through Celite. The filtrate was concentrated under reduced pressure until a small amount of white solid had precipitated and then kept at −25 °C overnight, after which the product had crystallised. The solid was separated by filtration, washed with *n*-hexane (2×5 mL) and then dried under vacuum to give the product as a white crystalline solid, which was stored at −25 °C (917 mg, 6.86 mmol, 53 %). ^1^H NMR (400 MHz, [D_6_]benzene, 23 °C, TMS): *δ*=1.44–1.62 (m, 4 H; β-CH_2_), 3.11–3.31 ppm (m, 4 H; α-CH_2_); ^13^C NMR (101 MHz, [D_6_]benzene, 23 °C, TMS): *δ*=26.6 (s; β-CH_2_), 26.8 (s; β-CH_2_), 26.9 (s; β-CH_2_), 54.9 (s; α-CH_2_), 55.3 (s; α-CH_2_), 55.5 ppm (s, α- CH_2_); elemental analysis calcd (%) for C_7_H_8_CuN: C 35.94, H 6.03, N 10.48; found: C 35.71, H 5.85, N 10.33.

**Preparation of copper(I) piperidide (4)**: A solution of copper(I) mesityl (1.462 g, 8.00 mmol) in tetrahydrofuran (10 mL) was treated with piperidine (3.95 mL, 40.00 mmol) at room temperature. Soon after the addition, a yellow precipitate formed which slowly dissolved over time with stirring (can take over 24 h to dissolve). The resultant pale yellow solution was stirred at room temperature for 3 days, during which a white precipitate had formed. The mixture was evaporated to dryness under vacuum and then *n*-hexane (10 mL) was added to suspend a white solid. The solid was separated by filtration, washed with *n*-hexane (2×5 mL) and then dried under vacuum to afford the product as a white powder (781 mg, 5.29 mmol, 66 %). ^1^H NMR (400 MHz, [D_6_]benzene, 23 °C, TMS): *δ*=1.51–1.76 (m, 6 H; β-CH_2_ and γ-CH_2_), 3.14–3.37 ppm (m, 4 H; α-CH_2_); ^13^C NMR (101 MHz, [D_6_]benzene, 23 °C, TMS) *δ*=27.0 (s; γ-CH_2_), 33.7 (s; β-CH_2_), 34.0 (s; β-CH_2_), 34.3 (s; β-CH_2_), 56.8 (s; α-CH_2_), 57.0 (s; α-CH_2_), 57.2 ppm (s; α-CH_2_); elemental analysis calcd (%) for C_5_H_10_CuN: C 40.66, H 6.82, N 9.48; found: C 40.61, H 6.84, N 9.36. Colourless crystals suitable for X-ray diffraction were obtained by leaving the pale yellow solution mentioned above at room temperature over 3 days.

**Preparation of copper(I) benzylamide (5)**: A solution of copper(I) mesityl (439 mg, 2.40 mmol) in tetrahydrofuran (1 mL) was treated with benzylamine (288 μL, 2.64 mmol) at room temperature. After stirring at room temperature for 2 min, it was evaporated under vacuum to give a yellow residue. The yellow residue was stirred in *n*-hexane (6 mL) for 30 min at room temperature and then the resultant solid was filtered. The filtered solid was washed with *n*-hexane (2×5 mL) and then dried under vacuum to give the product as a white powder (304 mg, 1.79 mmol, 75 %). ^1^H NMR (400 MHz, [D_6_]benzene, 24 °C, TMS): *δ*=0.13–0.34 (m, 1 H; N-H), 3.80–3.97 (m, 2 H; CH_2_), 7.04–7.15 (m, 1 H; *p*-C_6_H_5_), 7.17–7.38 ppm (m, 4 H; *o*-C_6_H_5_ and *m*-C_6_H_5_); ^13^C NMR (101 MHz, [D_6_]benzene, 22 °C, TMS): *δ*=52.6 (s; CH_2_), 126.4 (s; Ph carbon), 127.8 (s; Ph carbon), 128.3 (s; Ph carbon), 146.6 ppm (s; Ph carbon); elemental analysis calcd (%) for C_7_H_8_CuN: C 49.55, H 4.75, N 8.25; found: C 49.39, H 4.65, N 8.35.

**Isolation of the intermediate in the synthesis of copper(I) piperidide (6)**: A solution of copper(I) mesityl (365 mg, 2.00 mmol) in tetrahydrofuran (2 mL) was treated with piperidine (988 μL, 10.00 mmol) and stirred at room temperature for 5 min after which a yellow precipitate was present. The solid was filtered and then washed *n*-hexane (3×3 mL) and then dried under vacuum to afford the product as a yellow powder (225 mg, 0.25 mmol, 50 %). ^1^H NMR (400 MHz, [D_6_]benzene, 21 °C, TMS): *δ*=0.47 (p, ^3^*J*(H,H)=5.4 Hz, 2 H; N-H), 1.01–1.20 (m, 12 H; β-CH_2_ and γ-CH_2_), 2.04 (s, 12 H; *p*-CH_3_), 2.18–2.35 (m, 8 H; α-CH_2_), 2.93 (s, 24 H; *o*-CH_3_), 6.70 ppm (s, 8 H; Ar-H); ^13^C NMR (101 MHz, [D_6_]benzene, 22 °C, TMS) *δ*=21.0 (s; *p*-CH_3_), 24.7 (s; γ-CH_2_), 27.1 (s; β-CH_2_), 28.8 (s; *o*-CH_3_), 47.0 (s; α-CH_2_), 126.0 (s; Ar *C*-H), 138.2 (s; Ar *C*-CH_3_), 140.1 (s; Ar *C*-CH_3_), 152.3 ppm (s; Ar *C*-CH_3_); Unable to acquire satisfactory elemental analysis due to decomposition of the complex. Yellow crystals suitable for X-ray diffraction were obtained by treating a filtered yellow solution of copper(I) mesityl in THF with piperidine and then leaving it to stand at room temperature overnight.

**DFT calculations**: All calculations were performed with the Gaussian 09 package.[82] The B3LYP DFT method[83, 84] was used for geometry optimisations with the SVP basis set[85, 86] for Cu and 6–31G(d)[87, 88] for C, H, N and Si. Solvation effects on the structures were then finally taken into account by performing self-consistent reaction field (SCRF) calculations using the CPCM polarisable conductor calculation model with benzene set as the solvent.[89, 90] All optimised geometries were confirmed as energy minima with no imaginary frequencies using frequency calculations at the same level of theory. All volume calculations were performed on the fully optimised geometries with the density contour chosen as 0.02 electrons Bohr^−3^ and the number of Monte Carlo points was increased to 100 to minimise variation. The calculations were repeated 10 times for each compound and the mean of the recommended radii from the volume calculation outputs were calculated and used as *r*_calc_.

**Stoichiometric C—N coupling reactions between the copper(I) amide complexes and iodobenzene**: [D_6_]DMSO or [D_6_]benzene (1 mL) was added to a screw-cap vial containing the copper(I) amide complex (0.10 mmol), 1,10-phenanthroline (18 mg, 0.10 mmol; if required) and a magnetic stirring flea followed by mesitylene (14.0 μL, 0.10 mmol) as an internal standard and then iodobenzene (16.8 μL, 0.15 mmol) in a nitrogen-filled glovebox. The vial was capped tightly and then taken out of the glovebox. The mixtures were then stirred at 80 °C using an oil bath for 18 h. The contents of the vial were then passed through a syringe filter to remove any solid in a glovebox or nitrogen-filled glove bag into a NMR tube. The filtered solution was then analysed by ^1^H NMR spectroscopy to calculate the quantity of coupling product formed.

**Catalytic reactions between alkylamines and iodobenzene**: [D_6_]DMSO (800.0 μL) was added to a screw-cap vial containing potassium carbonate (28 mg, 0.20 mmol) and a magnetic stirring flea followed by copper(I) iodide (100 μL of 0.1 m solution in [D_6_]DMSO, 0.01 mmol), 1,10-phenanthroline (100 μL of 0.1 m solution in [D_6_]DMSO, 0.01 mmol; if required), mesitylene (14.0 μL, 0.10 mmol) as an internal standard, iodobenzene (111.0 μL, 1.00 mmol) and then the amine (0.1 mmol) in a nitrogen-filled glovebox. The vial was capped tightly and then taken out of the glovebox. The mixtures were then stirred at 80 °C using an oil bath for 18 h. The contents of the vial were then passed through a syringe filter to remove any solid in a glovebox or nitrogen-filled glove bag into a NMR tube. The filtered solution was then analysed by ^1^H NMR spectroscopy to calculate the quantity of coupling product formed.
