# Supplementary material for: Synthesis, Characterisation and Reactivity of Copper(I) Amide Complexes and Studies on Their Role in the Modified Ullmann Amination Reaction
Source: Chemistry. 2015 Mar 18;21(19):7179–92. doi: 10.1002/chem.201405699 (PMC4471577; doi:10.1002/chem.201405699)
Supplement: Supplementary file 1 [file chem0021-7179-sd1.pdf]

CHEMISTRY
A European Journal

Supporting Information

Synthesis, Characterisation and Reactivity of Copper(I) Amide Complexes and Studies on Their Role in the Modified Ullmann Amination Reaction

Simon Sung,[a] D. Christopher Braddock,[a] Alan Armstrong,[a] Colin Brennan,[b] David Sale,[b] Andrew J. P. White,[a] and Robert P. Davies*[a]

chem_201405699_sm_miscellaneous_information.pdf

Electronic Supplementary Information

Synthesis, Characterisation and Reactivity of Copper(I) Amide Complexes and Studies on their Role in the Modified-Ullmann Amination Reaction

Simon Sung[a], D. Christopher Braddock[a], Alan Armstrong[a], Colin Brennan[b], David Sale[b] Andrew J. P. White[a] and Robert P. Davies*[a]

[a] *Department of Chemistry, Imperial College London, South Kensington, London, SW7 2AZ, UK*

[b] *Process Studies Group, Syngenta, Jealott's Hill Research Centre, Bracknell, Berkshire RG42 6EY, UK*

**Corresponding Author*

E-mail: r.davies@imperial.ac.uk

Tel: +44 (0)207 5945754

Contents

1 Experimental 3

2 NMR spectra of complexes 7

3 DFT optimised structures 19

4 ^{1}H DOSY NMR of the copper(I) amide complexes with internal standards 25

5 ^{1}H NMR data of 3 and 4 at 0.05 and 0.20 M concentration in [D_6]benzene 35

6 ^{1}H DOSY NMR data of 3 and 4 in the presence of 1,10-phenanthroline and internal standards 37

7 NMR data of 3 and 4 in the presence of 1,10-phenanthroline in [D_6]DMSO 39

8 ESI-MS data 47

9 X-Ray Crystallography 49

9.1 The X-ray crystal structure of 1 51

9.2 The X-ray crystal structure of 2 51

9.3 The X-ray crystal structure of 4 52

9.4 The X-ray crystal structure of 6 53

9.5 The X-ray crystal structure of 6b 54

10 References 57

1 Experimental

General information: All manipulations were carried out under a nitrogen atmosphere in a glovebox or using standard Schlenk techniques. Glassware was dried at a minimum temperature of 120 °C for at least 12 h prior to use. Toluene and *n*-hexane were dried and degassed over activated alumina using an Innovative Technologies PureSolv solvent purification system. Tetrahydrofuran was freshly distilled over sodium and benzophenone under a nitrogen atmosphere prior to use. Potassium carbonate was dried under vacuum at 180 °C for 48 h and 1,10-phenanthroline was dried under vacuum at 85 °C for 36 h. Copper(I) mesityl was prepared according to a reported procedure by Tsuda *et al*.[1] Dimethyl sulfoxide and [D_6]DMSO were distilled over calcium hydride under vacuum and stored over 4 Å molecular sieves under a nitrogen atmosphere. All amines used were distilled over calcium hydride or sodium and then stored over 4 Å molecular sieves under a nitrogen atmosphere. All other chemicals were used as received from their respective suppliers. All products, other reagents and deuterated NMR solvents were stored in a nitrogen-filled glovebox, where liquid reagents were stored over 4 Å molecular sieves.

^{1}H and ^{13}C NMR spectroscopy data were obtained at room temperature using Bruker AV-400 spectrometers except for ^{1}H DOSY and ^{1}H-^{1}H ROESY NMR which were recorded on a Bruker AV-500. Elemental analyses were performed at the London Metropolitan University. ESI-MS mass spectra were acquired on a Waters LCT Premier. X-ray crystallography data were collected using Oxford Diffraction Xcalibur 3 (**1** and **2**), Oxford Diffraction Xcalibur PX Ultra (**4**), and Agilent Xcalibur 3 E (**6**) diffractometers, and the structures were refined using the SHELXTL, SHELX-97, and SHELX-2013 program systems.[2–4] The absolute structures of **1**, **4** and **6** were determined by use of the Flack parameter (0.037(9), 0.07(3) and 0.070(11) respectively). CCDC codes for **1**, **2**, **4** and **6** are 1027216, 1027217, 1027218 and 1027219 respectively.

The yields reported for the syntheses of the copper(I) amide complexes were calculated from the amount isolated after purification. The quantities of each compound present after the catalytic and stoichiometric reactions were calculated by NMR through the use of mesitylene as an internal standard and are reported as a mean of at least two independent runs. ^{1}H and ^{13}C NMR data were analysed using MESTRELAB MestReNova; ^{1}H-^{1}H ROESY NMR data were analysed using Bruker TopSpin; and ^{1}H DOSY NMR data were processed and analysed using Bruker Dynamics Centre and TopSpin respectively.

Preparation of ^{1}H DOSY NMR solutions of the copper(I) amide complexes with internal standards: Copper(I) amide complex (0.04 mmol, 0.05 M) and 1,2,3,4-tetraphenylnaphthalene (15 mg, 0.04 mmol, 0.05 M) were added to a glass vial and then dissolved in [D_6]benzene (668 μL). 1-phenylnaphthalene (13 μL, 0.07 mmol, 0.10 M) and tetramethylsilane (19 μL, 0.14 mmol, 0.20 M) were then added. Finally, the solution was transferred to a J. Youngs tap NMR tube for analysis. (Amount of copper(I) amide complex was adjusted for the desired concentration).

Preparation of the NMR solution of 3 and 4 in the presence of 1,10-phenanthroline and DOSY NMR internal standards in [D_6]benzene: Complex **3** or **4** (0.04 mmol, 0.05 M), 1,10-phenanthroline (6 mg, 0.04 mmol, 0.05 M) and 1,2,3,4-tetraphenylnaphthalene (15 mg, 0.04 mmol, 0.05 M) were added to a glass vial and then dissolved in [D_6]benzene (668 μL) to give a blue solution. 1-phenylnaphthalene (13 μL, 0.07 mmol, 0.10 M) and tetramethylsilane (19 μL, 0.14 mmol, 0.20 M) were then added. Finally, the solution was transferred to a J. Youngs tap NMR tube for analysis.

Preparation of the NMR solution of 3 and 4 in the presence of 1,10-phenanthroline in [D_6]DMSO: A colourless solution of 1,10-phenanthroline (18 mg, 0.10 mmol) in [D_6]DMSO (1 mL) was added to a vial containing complex **3** or **4** (0.10 mmol) and was then stirred for 18 h at room temperature to give a dark red solution. The solution was then transferred to a J. Youngs tap NMR tube.

Preparation of the NMR solution of lithium amidocuprates in [D_6]DMSO for comparison: Solutions of pyrrolidine or piperidine (5.5 mmol) in tetrahydrofuran (10 mL) were treated dropwise with *n*-butyllithium in hexanes (2 mL, 5 mmol, 2.5 M) at 0 °C. After complete addition, the solutions were allowed to come to room temperature and stirring was continued for 1 h. The solutions were then evaporated to dryness until a white powder remained. The solid was dried under vacuum at 40 °C to give the corresponding lithium amides as a white powder. The lithium amides were then isolated in a nitrogen-filled glove box. Solutions of the lithium amides (0.1 mmol) in [D_6]DMSO (500 μL) in vials were then treated dropwise with solutions of copper(I) iodide (0.05 mmol) in [D_6]DMSO (500 μL) to generate the corresponding lithium amidocuprates. The solutions were then transferred to a J. Youngs tap NMR tubes for analysis.

Preparation of the ESI-MS solution of 4 in the presence of 1,10-phenanthroline in dimethyl sulfoxide: Dimethyl sulfoxide (1 mL) was added to a vial containing complex **4** (5 mg, 0.03 mmol) and 1,10-phenanthroline (6 mg, 0.03 mmol) and a magnetic stirring flea. The mixture was stirred overnight at room temperature to give a deep-red solution with a small amount of unwanted solid. The contents of the vial were passed through a syringe filter to give a clear deep-red solution. 9.1 μL of the filtrate was then taken and diluted to 1 mL using dimethyl sulfoxide to produce a pale yellow solution with the desired 0.1 mg mL^{-1} concentration for analysis.

Preparation of the ESI-MS solution of 4 in the presence of 1,10-phenanthroline in benzene: Benzene (1 mL) was added to a vial containing complex **4** (5 mg, 0.03 mmol) and 1,10-phenanthroline (6 mg, 0.03 mmol) and a magnetic stirring flea. The mixture was stirred for 10 min at room temperature to give a deep-blue solution. 9.1 μL of the solution was taken and diluted to 1 mL using benzene to produce a virtually colourless solution with the desired 0.1 mg mL^{-1} concentration for analysis.

Procedure for the stoichiometric reaction between the lithium bis(piperidido)cuprate(I) and iodobenzene: A solution of lithium bis(piperidido)cuprate(I) (0.05 mmol) in $[D_6]$DMSO (1 mL) was made as described previously in a screw-cap vial. Mesitylene (14.0 μL, 0.10 mmol) as an internal standard and iodobenzene (16.8 μL, 0.15 mmol) were added and then the vial was capped tightly and taken out of the glovebox. The solutions were then stirred at 80 °C in an oil bath for 18 h. In a glovebox, the contents of the vial were then passed through a syringe filter into a NMR tube to remove any solid. The filtered solution was then analysed by ^{1}H NMR spectroscopy to calculate the quantity of coupling product formed.

Effect of the catalytic loading of 1,10-phenanthroline on the catalytic C-N coupling between piperidine and iodobenzene: $[D_6]$DMSO (volume required to make up to 1 mL total solvent) was added to a screw-cap vial containing potassium carbonate (28 mg, 0.20 mmol) and a magnetic stirring flea followed by copper(I) iodide (100 μL of 0.1 M solution in $[D_6]$DMSO, 0.01 mmol), ligand (25, 50, 75, 100, 200, 300 or 400 μL of 0.1 M stock solution, 0.0025, 0.005, 0.0075, 0.0100, 0.0200 or 0.0400 mmol), mesitylene (14 μL, 0.10 mmol) as an internal standard, iodobenzene (111 μL, 1.00 mmol) and then piperidine (10 μL,

0.10 mmol) in a nitrogen-filled glovebox. The vial was capped tightly and then taken out of the glovebox. The mixtures were then stirred at 80 °C using an oil bath for 18 h. The contents of the vial were then passed through a syringe filter to remove any solid in a glovebox or nitrogen-filled glove bag into a NMR tube. The filtered solution was then analysed by ^{1}H NMR spectroscopy to calculate the quantity of 1-phenylpiperidine product formed.

2 NMR spectra of complexes

The following NMR spectra were obtained using Bruker AV-400 spectrometers at 400 MHz between 21 and 24 °C.

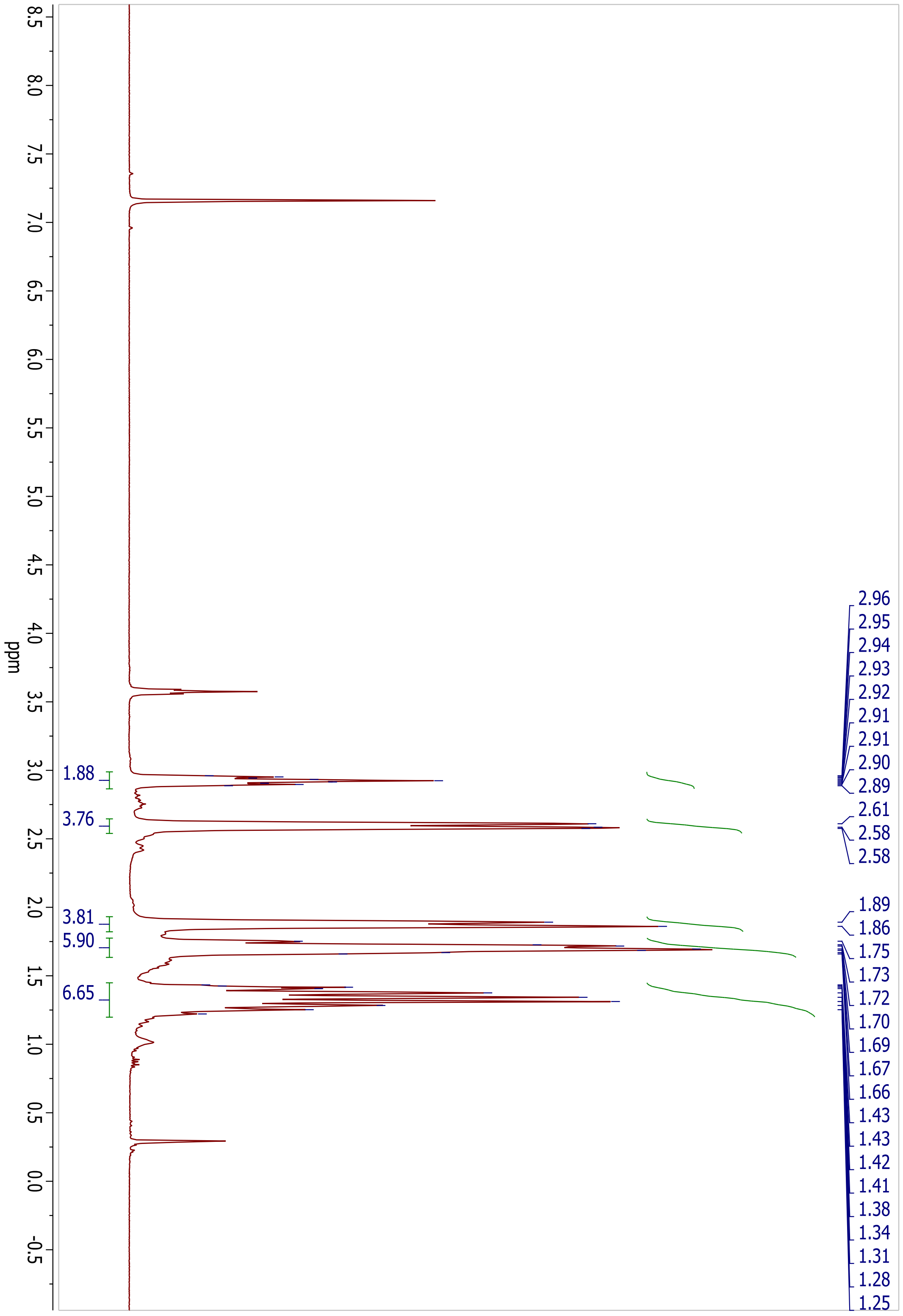

Figure S1 ^{1}H NMR spectrum of **1** in [D_6]benzene

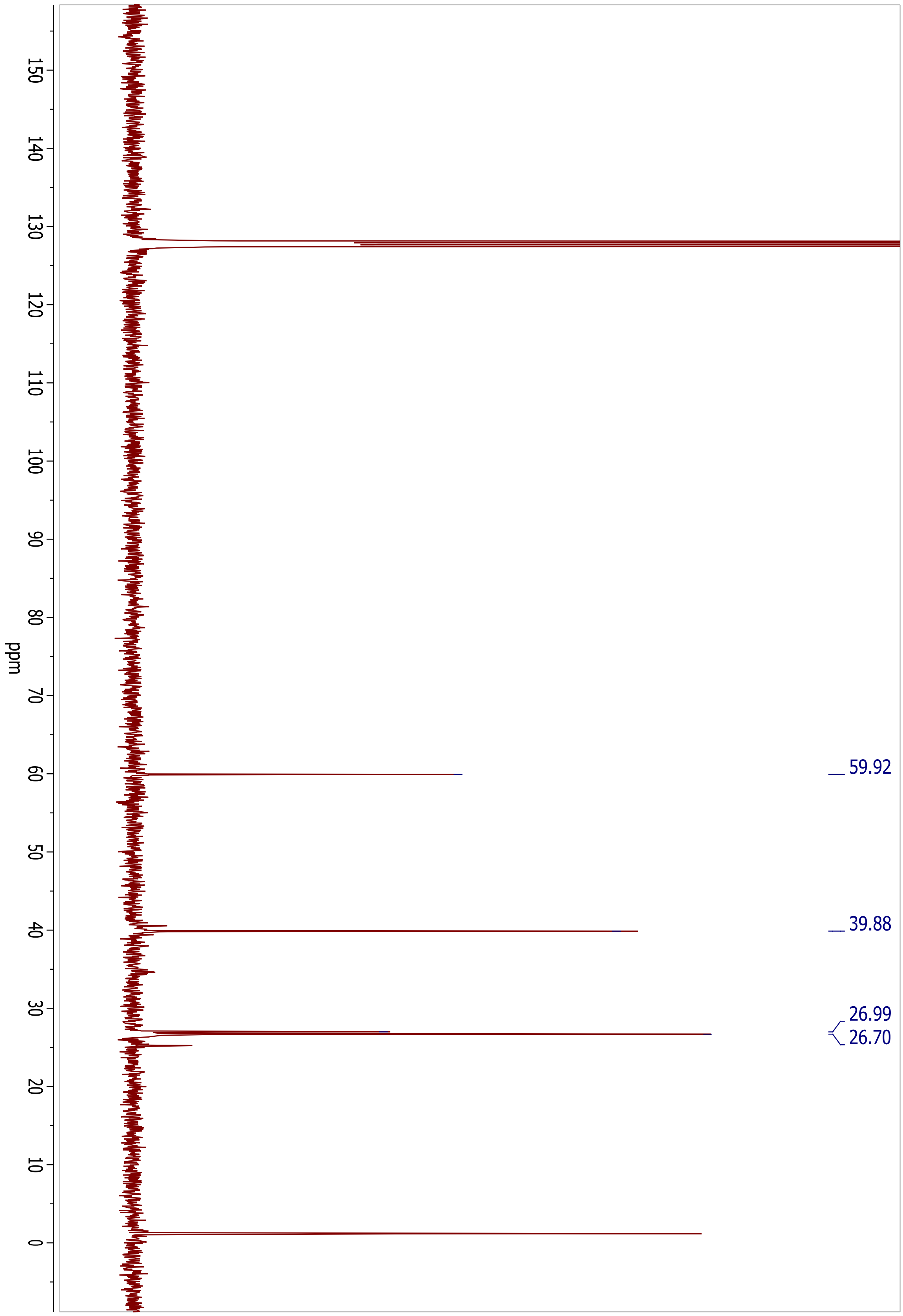

Figure S2 ^{13}C NMR spectrum of **1** in [D_6]benzene

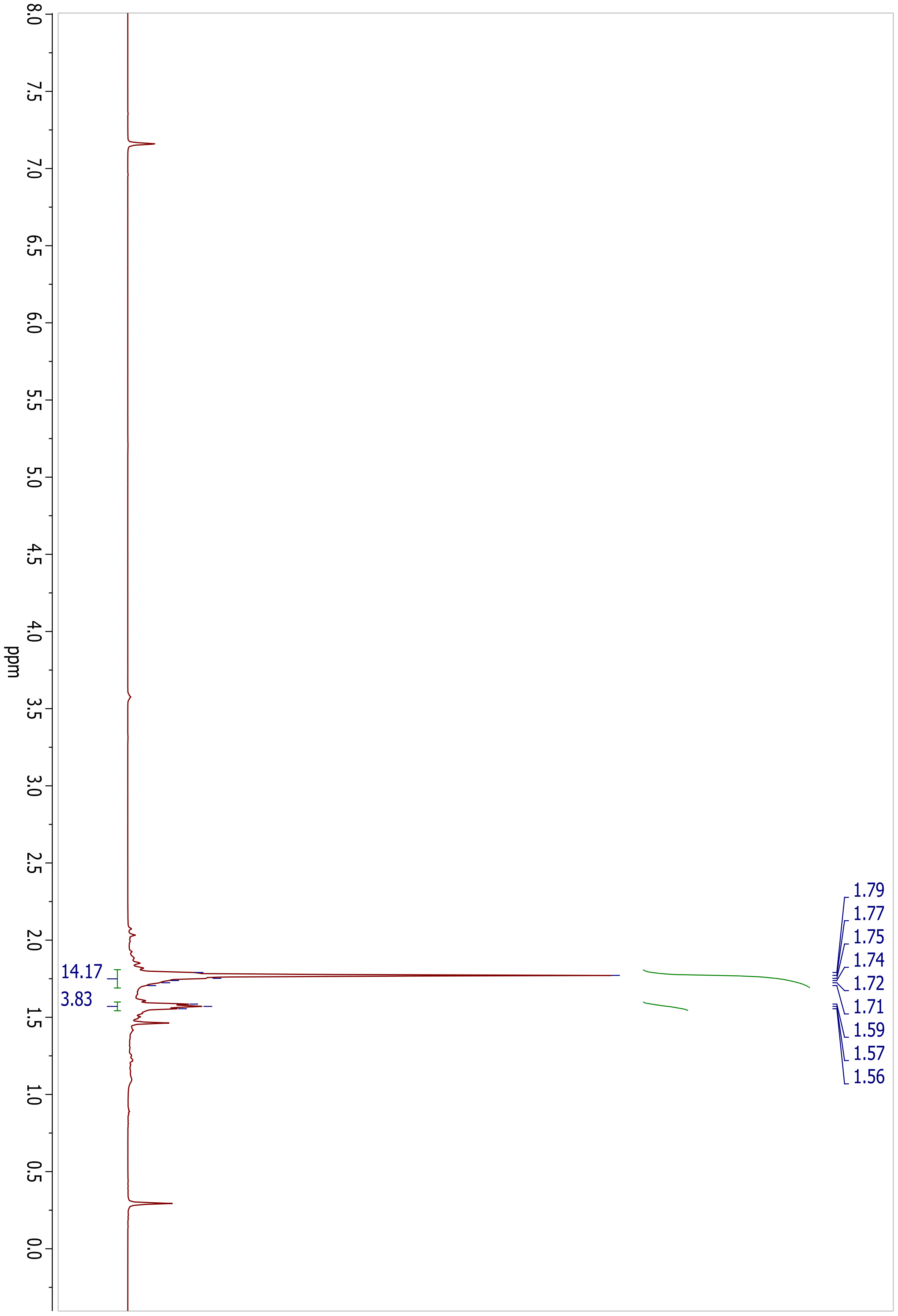

Figure S3 ^{1}H NMR spectrum of **2** in [D$_6$]benzene

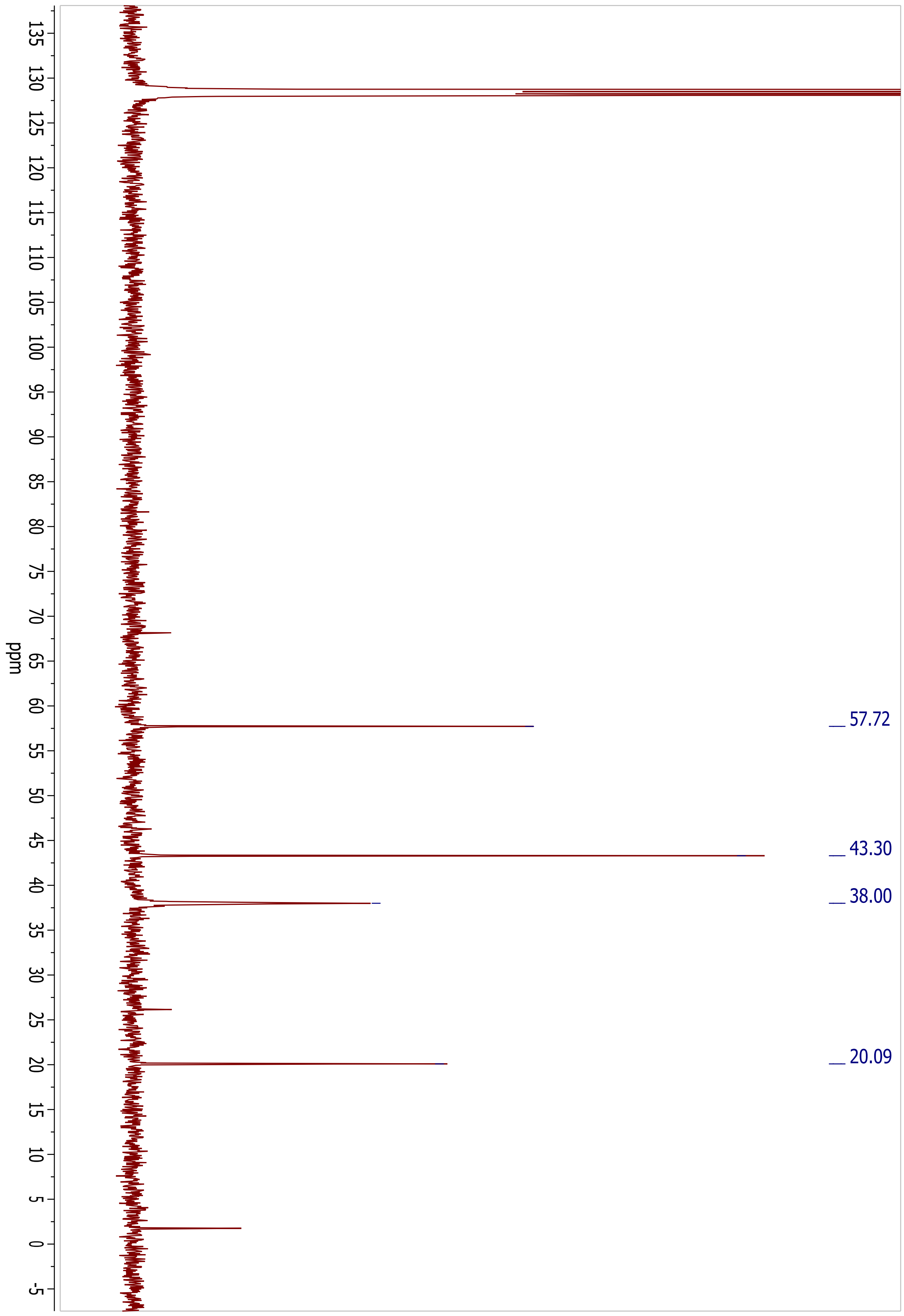

Figure S4 ^{13}C NMR spectrum of **2** in [D$_6$]benzene

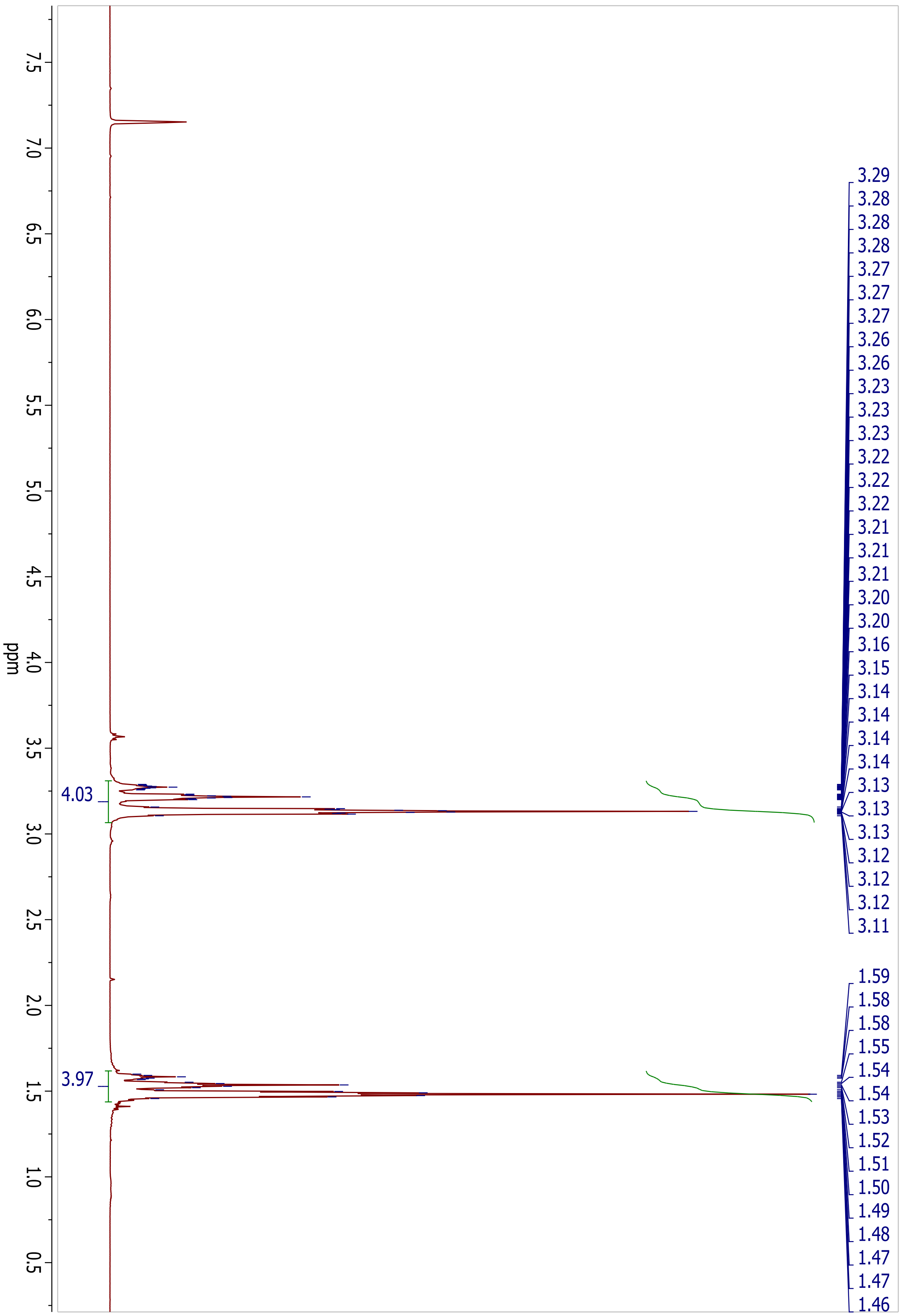

Figure S5 ^{1}H NMR spectrum of **3** in [D$_6$]benzene

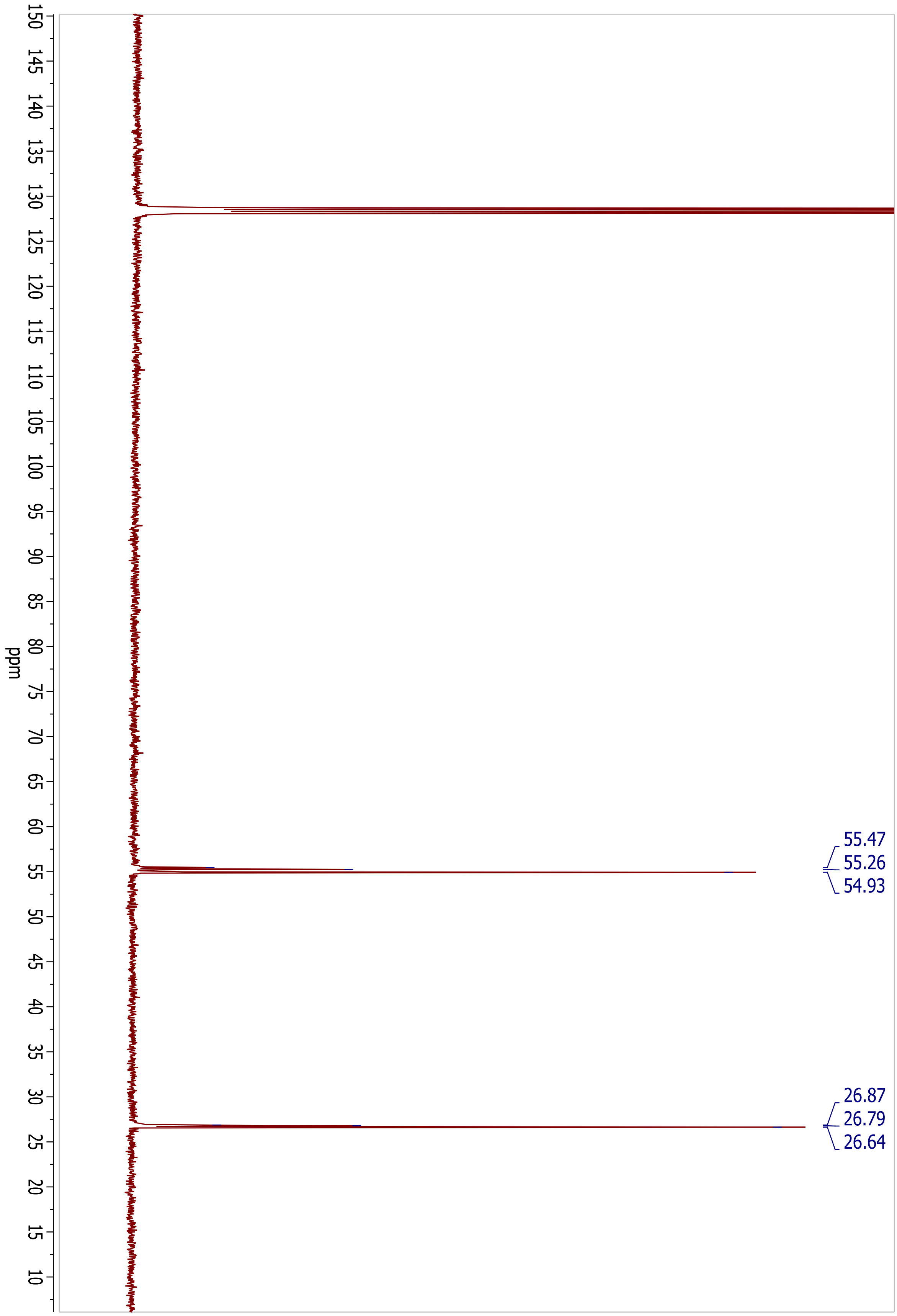

Figure S6 ^{13}C NMR spectrum of **3** in [D$_6$]benzene

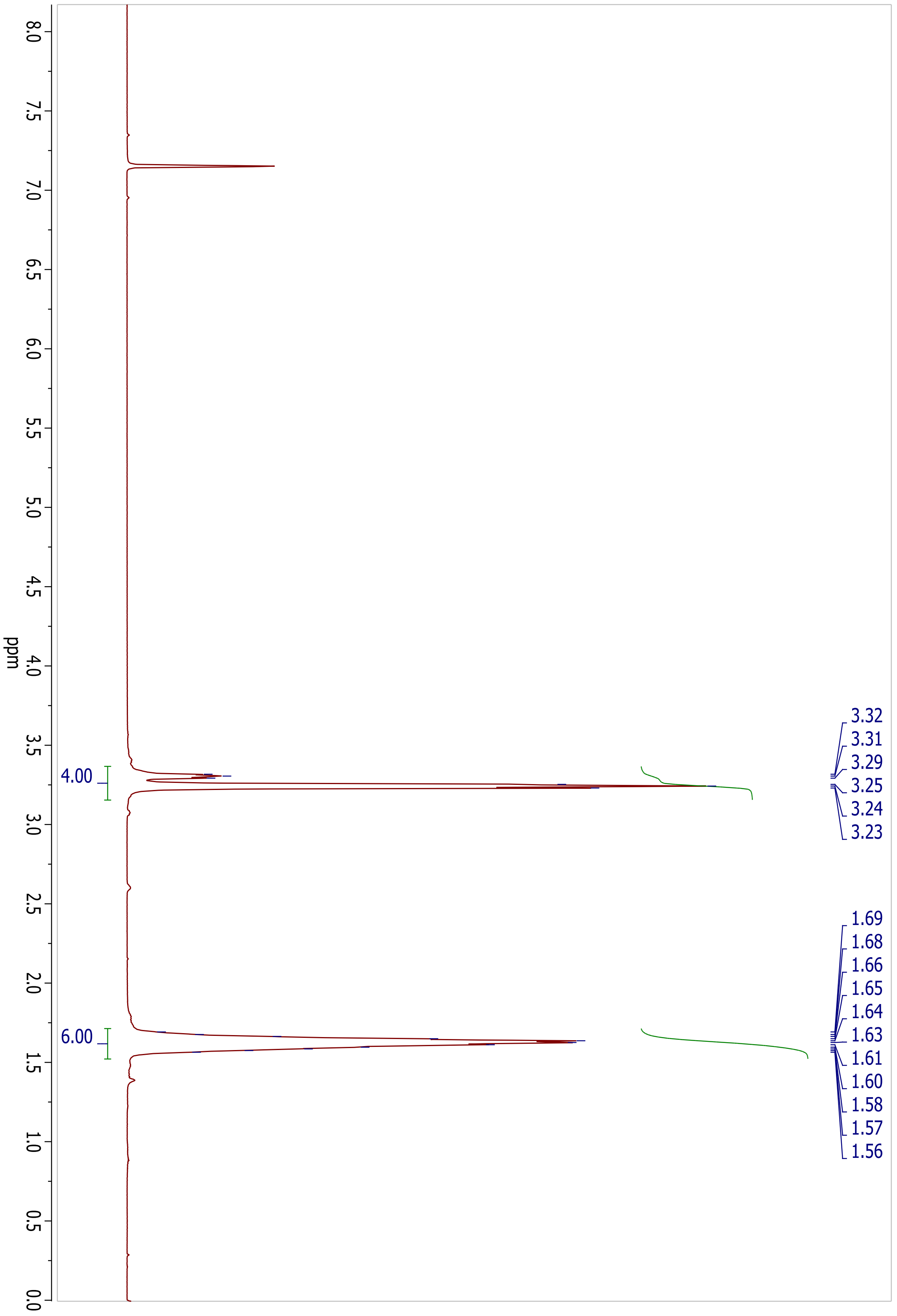

Figure S7 ^{1}H NMR spectrum of **4** in [D$_6$]benzene

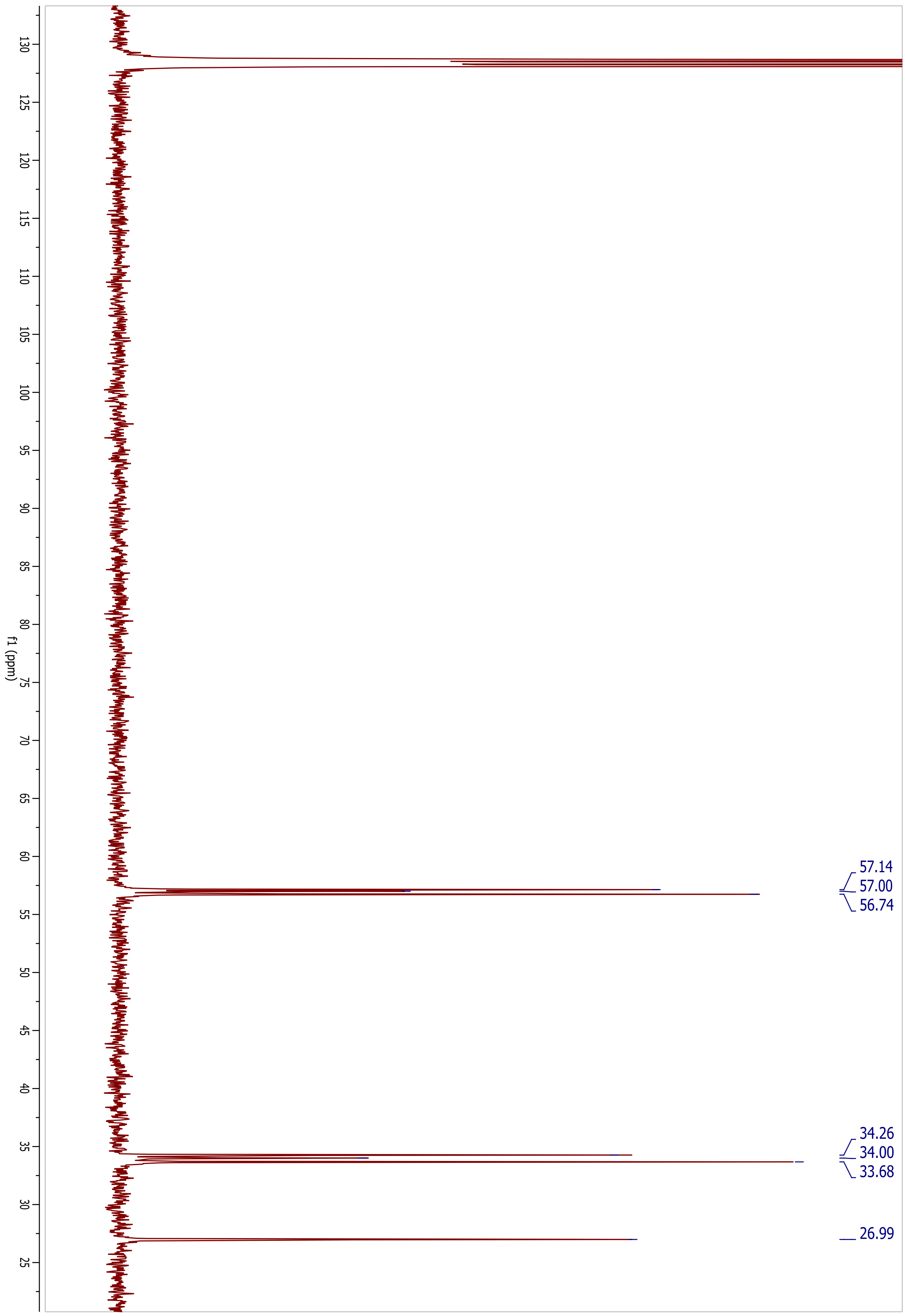

Figure S8 ^{13}C NMR spectrum of **4** in [D_6]benzene

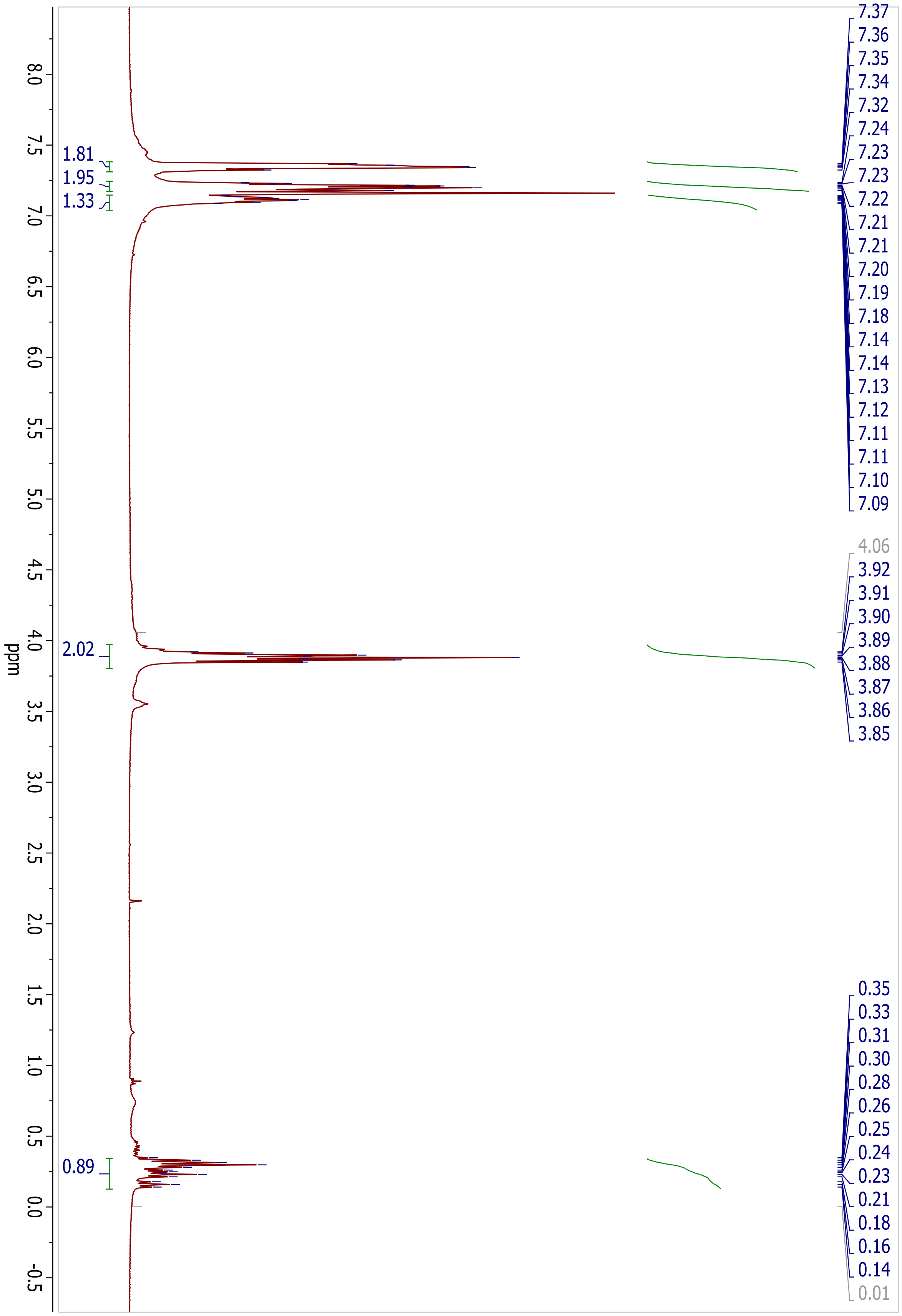

Figure S9 ^{1}H NMR spectrum of **5** in [D$_6$]benzene

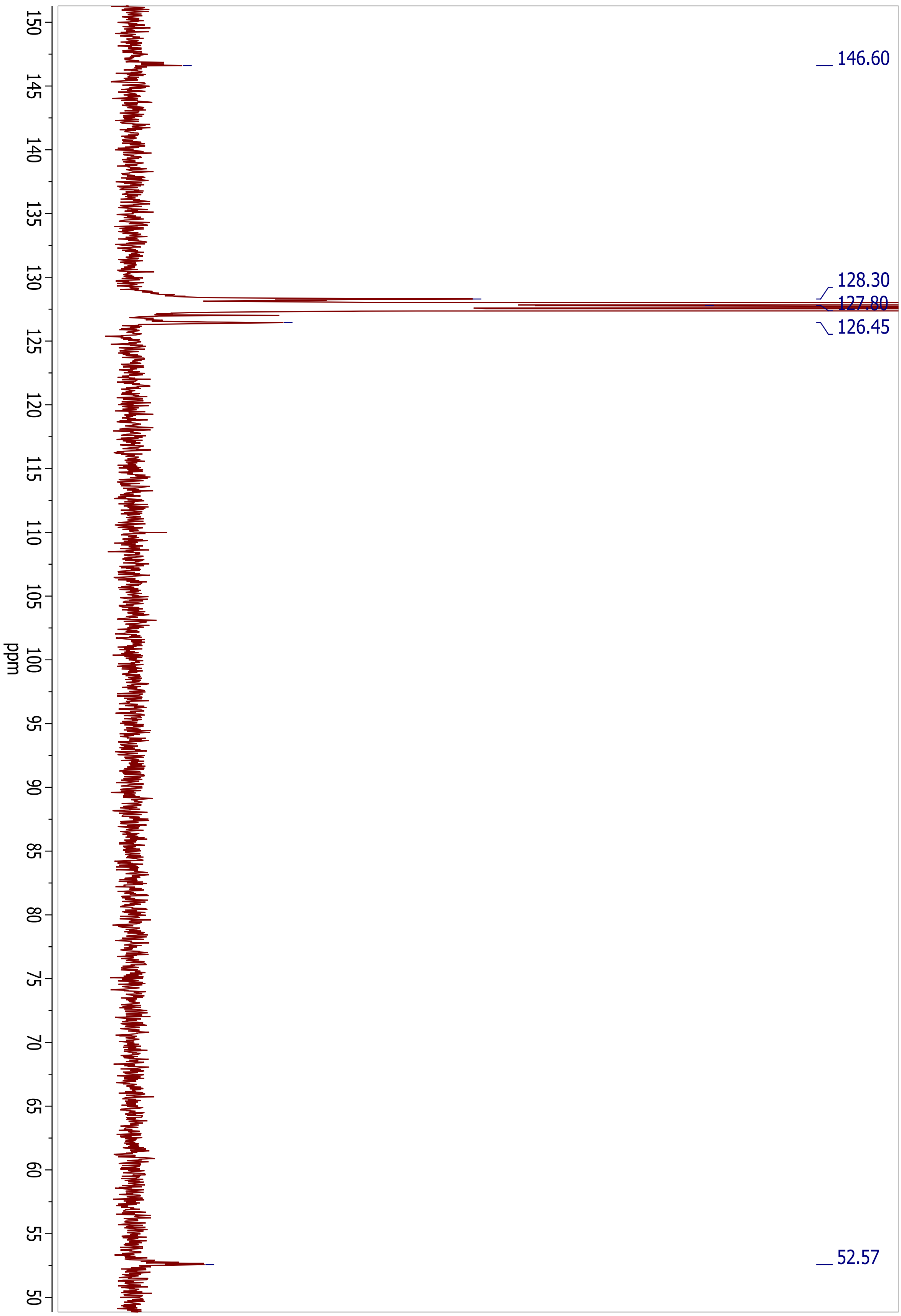

Figure S10 ^{13}C NMR spectrum of **5** in [D$_6$]benzene

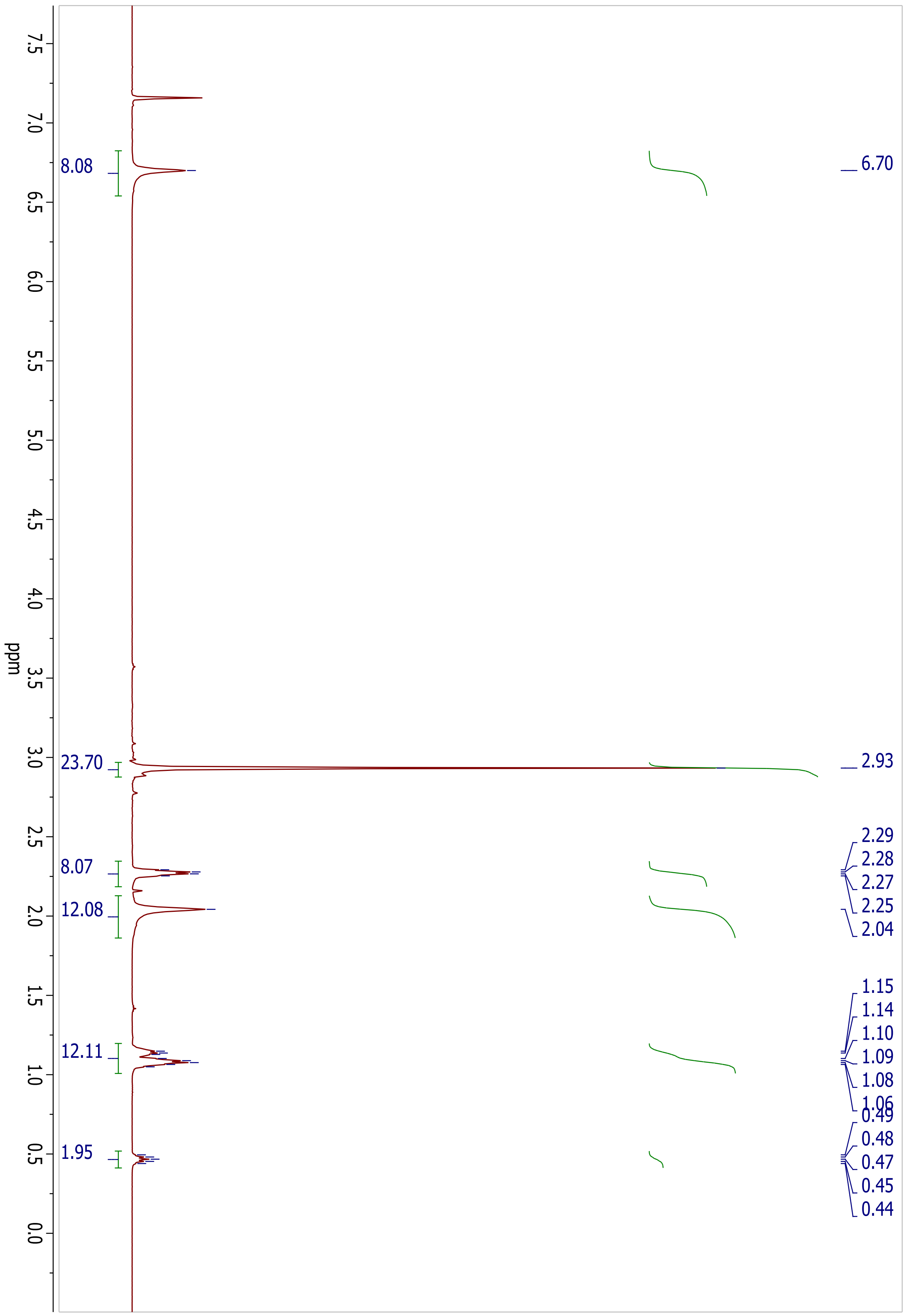

Figure S11 ^{1}H NMR spectrum of **6** in [D_6]benzene.

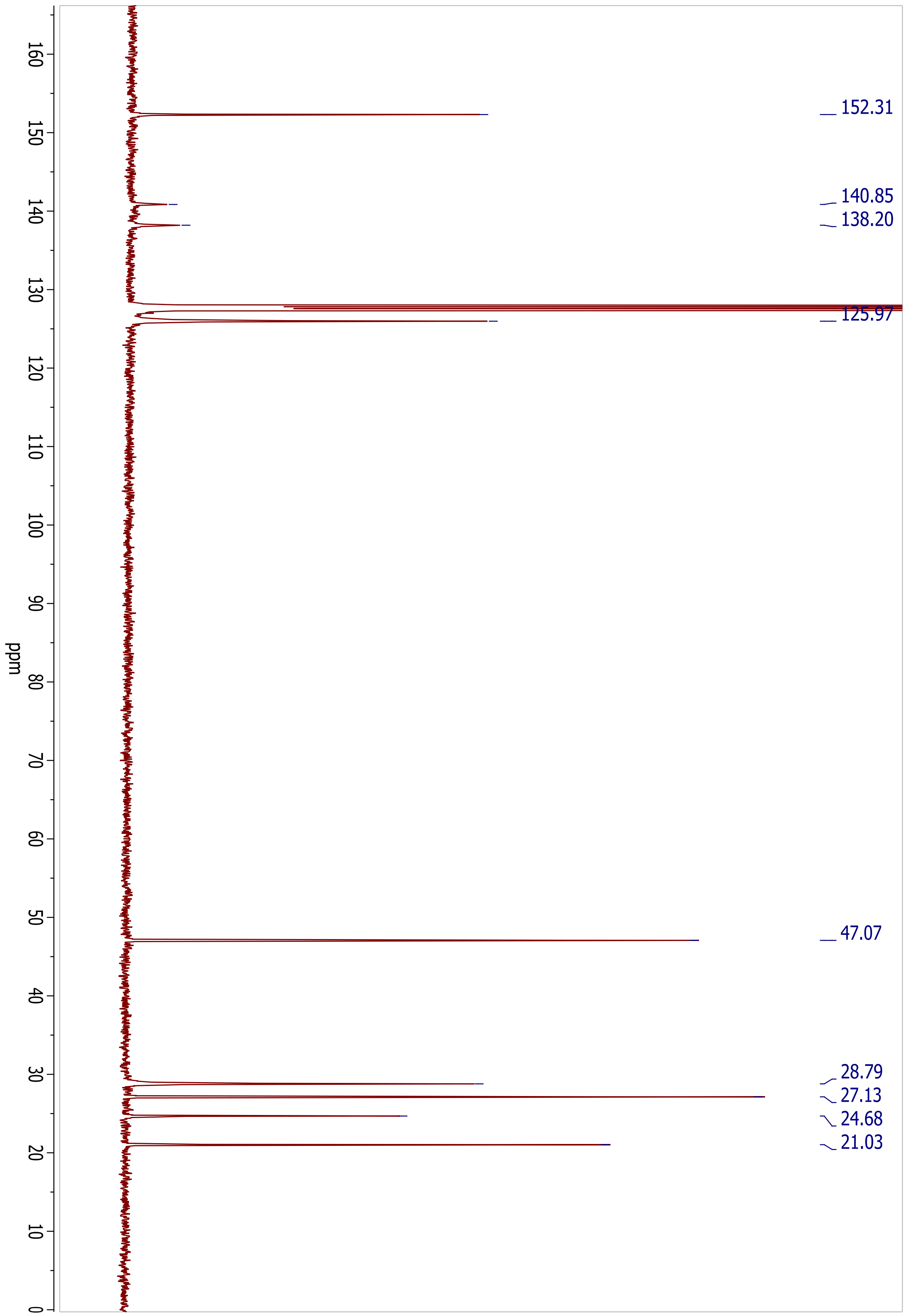

Figure S12 ^{13}C NMR spectrum of **6** in [D$_6$]benzene

3 DFT optimised structures

Grey = carbon; orange = copper; blue = N; green-grey = Si; hydrogen atoms are not shown for clarity. The methods used to calculate the energy minimised structures and the radii, r_{calc}, can be found in the full-text.

Optimised Structures for complex **1**		Symmetry	*E* / a.u.	r_{calc} / Å
Monomer	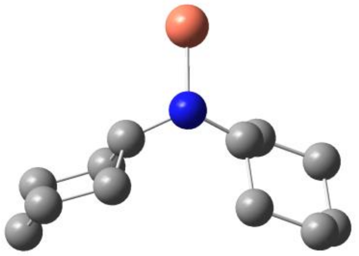	C_1	-2165.57772321	4.79
Dimer	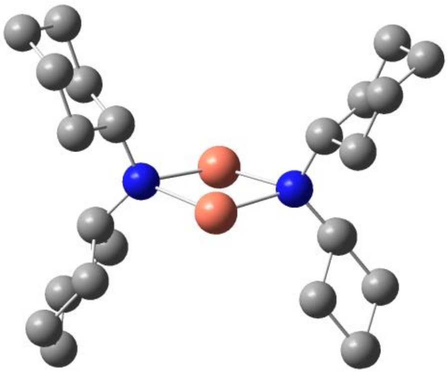	C_1	-4331.26892615	5.89
Trimer	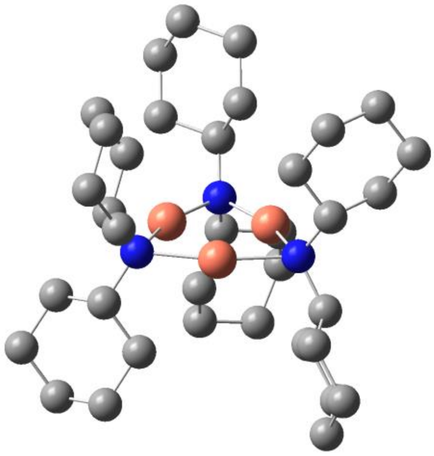	C_1	-6497.01507698	6.65
Tetramer	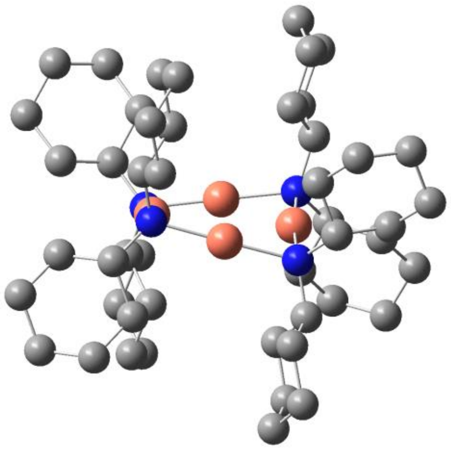	C_1	-8662.69577489	7.27

Table S1 DFT optimised structures for aggregates of complex **1**

Optimised Structures for complex **2**		Symmetry	*E* / a.u.	r_{calc} / Å
Monomer	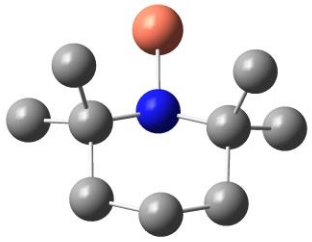	C_s	-2048.85455902	4.55
Dimer	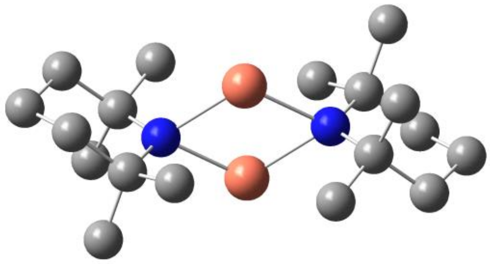	C_1	-4097.78202724	5.50
Trimer	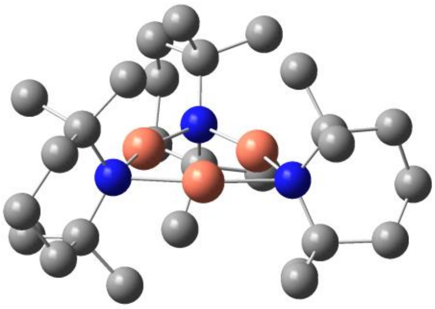	C_1	-6146.77412924	6.21
Tetramer	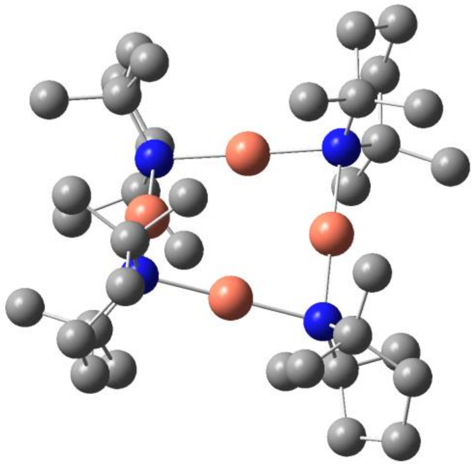	C_1	-8195.70768115	6.83

Table S2 DFT optimised structures for aggregates of complex **2**

	Optimised Structures for complex **3**	Symmetry	*E* / a.u.	r_{calc} / Å
Monomer	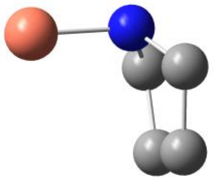	C_1	-1852.28085671	3.73
Dimer	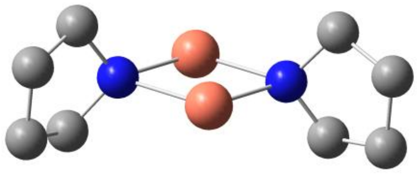	C_1	-3704.65330564	4.56
Trimer	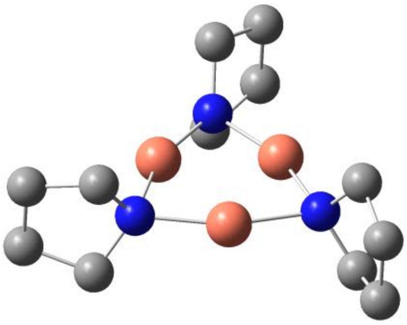	C_1	-5557.09175653	5.15
Tetramer	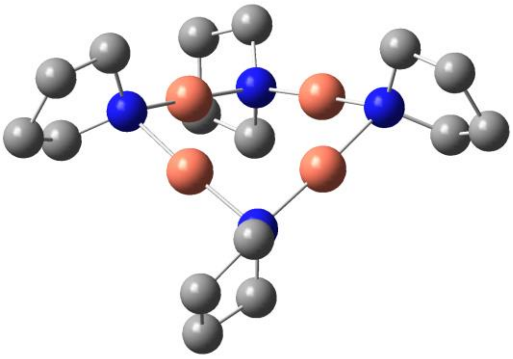	C_1	-7409.48008567	5.65
Pentamer	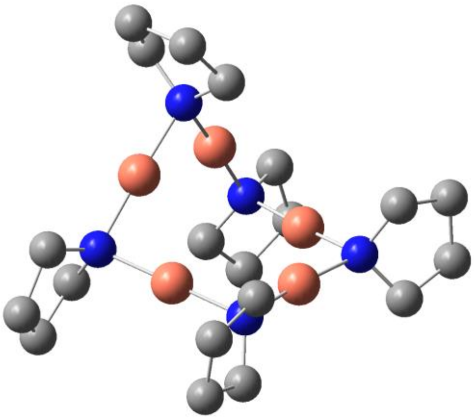	C_1	-9261.84999589	6.04

Table S3 DFT optimised structures for aggregates of complex **3**

Optimised Structures for complex **4**		Symmetry	*E* / a.u.	r_{calc} / Å
Monomer	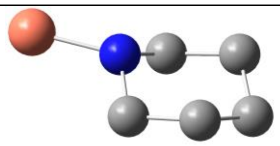	C_s	-1891.60028997	3.92
Dimer	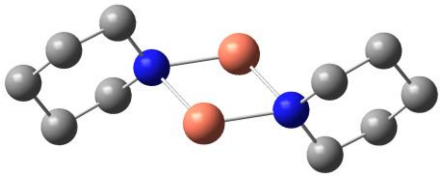	C_1	-3783.29551833	4.76
Trimer	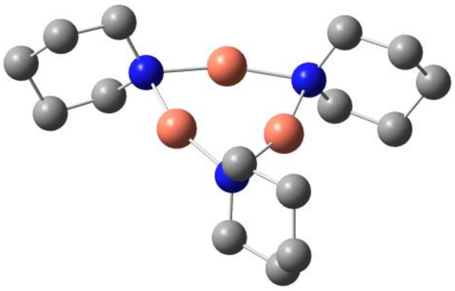	C_1	-5675.05265839	5.38
Tetramer	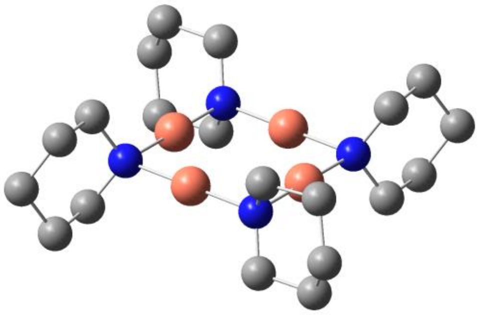	C_1	-7566.75916409	5.89
Pentamer	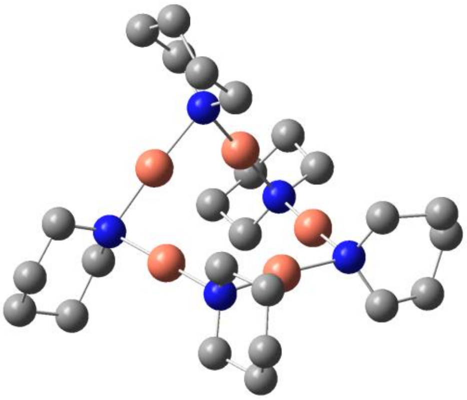	C_1	-9458.43820746	6.37

Table S4 DFT optimised structures for aggregates of complex **4**

Optimised Structures for complex **5**		Symmetry	*E* / a.u.	r_{calc} / Å
Monomer	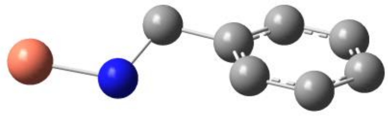	C_1	-1966.60407882	4.07
Dimer	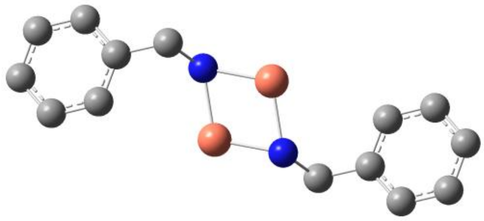	C_1	-3933.30251837	4.97
Trimer	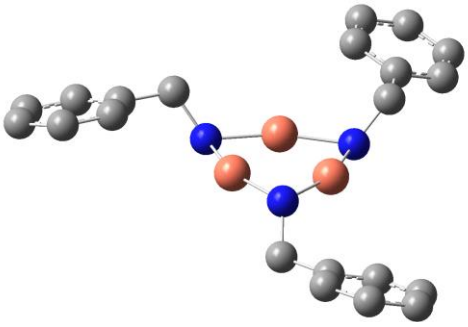	C_1	-5900.06726632	5.62
Tetramer	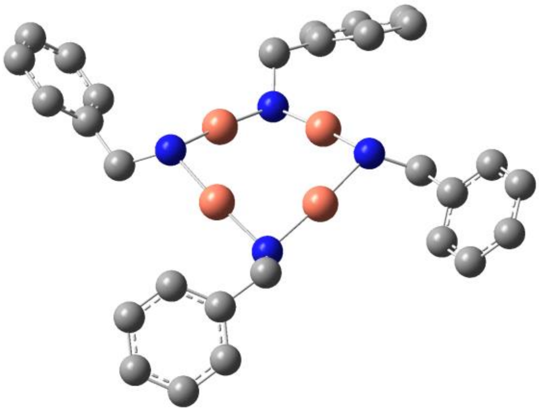	C_1	-7866.78345538	6.15

Table S5 DFT optimised structures for aggregates of complex **5**

Optimised Compounds		Symmetry	*E* / a.u.	r_{calc} / Å
1,2,3,4-tetraphenylnaphthalene	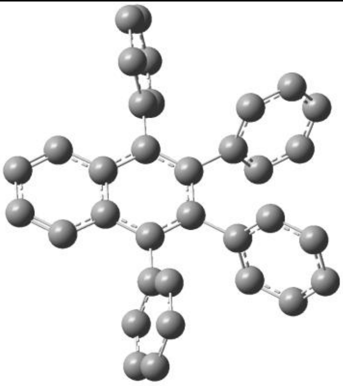	C_2	-1310.09911902	5.81
1-phenylnaphthalene	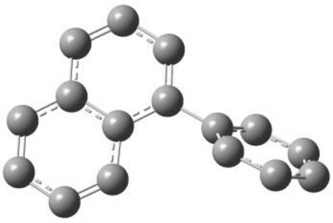	C_1	-616.94878610	4.65
Tetramethylsilane	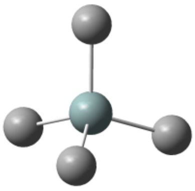	T_d	-449.19214745	3.90
1,10-phenanthroline	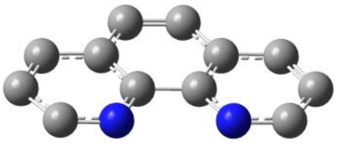	C_1	-571.61609373	4.40

Table S6 DFT optimised structures for aggregates of DOSY NMR standards and 1,10-phenanthroline

4 ^{1}H DOSY NMR of the copper(I) amide complexes with internal standards

When experimentally determined $Log_{10}D$ of the internal standards (1,2,3,4-tetraphenylnaphthlene, 1-phenylnaphthalene, and tetramethylsilane) were plotted against their $Log_{10}r$ values, linear regression lines were produced where $r^2 > 0.99$. Using these trendlines, the radii of the copper(I) amide aggregates in solution (r_{obs}) were determined using their observed $Log_{10}D$ values. The aggregates can then be identified by comparing r_{obs} with r_{calc} of DFT optimised copper(I) amide structures.

The following ^{1}H DOSY NMR spectra were obtained using a Bruker AV-500 spectrometer at 500 MHz and 25 °C.

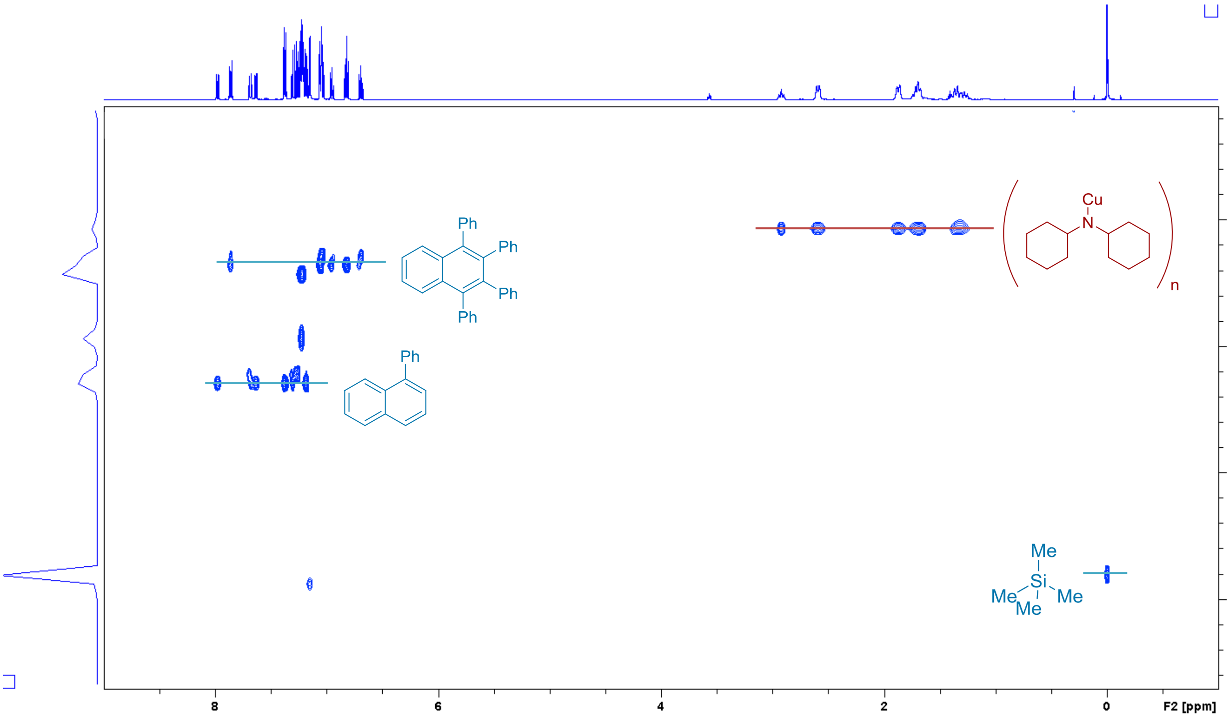

Figure S13 ^{1}H DOSY NMR spectrum of **1** with internal standards at 0.05 M concentration in [D$_6$]benzene

Compounds	r_{calc} / Å	Log(r_{calc} / Å)	D_{obs} / m^2s^{-1}	Log(D_{obs} / m^2s^{-1})
1,2,3,4-tetraphenylnaphthlene	5.81	0.764	6.67E-10	-9.18
1-phenylnaphthalene	4.65	0.668	1.15E-09	-8.94
Tetramethylsilane	3.90	0.591	1.90E-09	-8.72

y = -2.62x - 7.18 R^2 = 0.998

	D_{obs} / m^2s^{-1}	Log(D_{obs} / m^2s^{-1})	Calculated Log(r_{obs} / Å)	r_{obs} / Å
Complex 1	5.36E-10	-9.27	0.798	6.28

Table S7 *D-r* analysis from ^{1}H DOSY NMR data of **1** with internal standards at 0.05 M concentration in [D_6]benzene

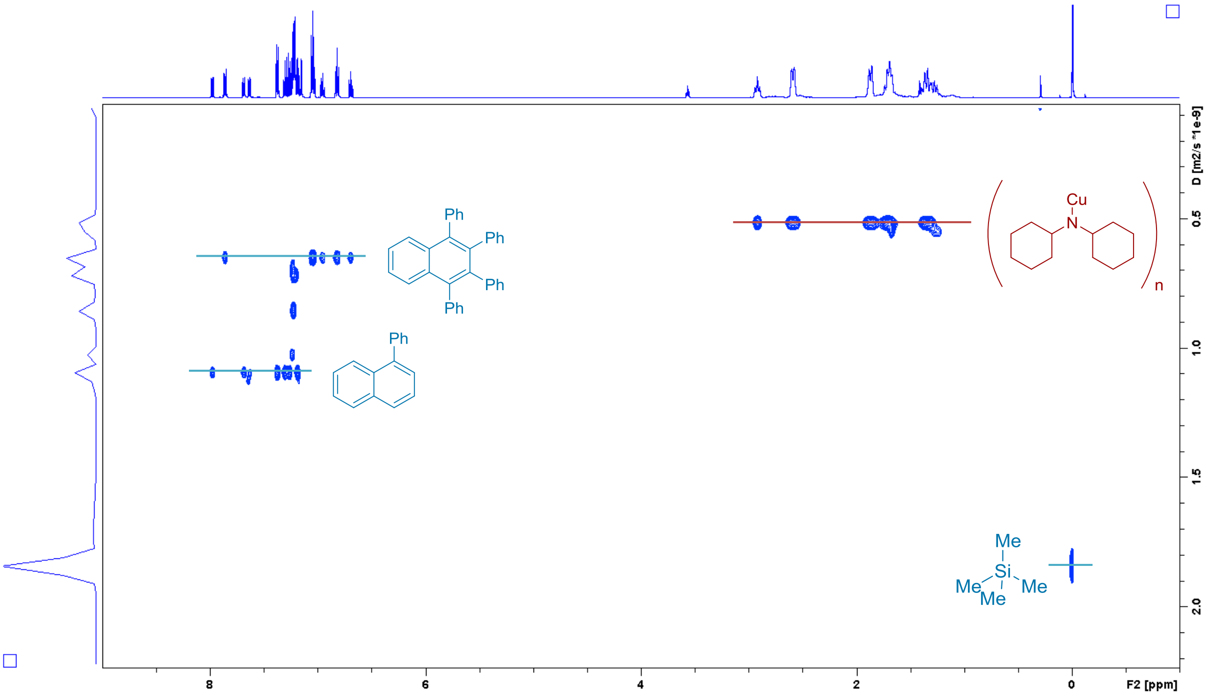

Figure S14 ^{1}H DOSY NMR spectrum of **1** with internal standards at 0.15 M concentration in [D_6]benzene

Compounds	r_{calc} / Å	Log(r_{calc} / Å)	D_{obs} / m^2s^{-1}	Log(D_{obs} / m^2s^{-1})
1,2,3,4-tetraphenylnaphthlene	5.81	0.764	6.53E-10	-9.18
1-phenylnaphthalene	4.65	0.668	1.10E-09	-8.96
Tetramethylsilane	3.90	0.591	1.84E-09	-8.73

y = -2.59x - 7.21 R^2 = 0.996

	D_{obs} / m^2s^{-1}	Log(D_{obs} / m^2s^{-1})	Calculated Log(r_{obs} / Å)	r_{obs} / Å
Complex 1	5.17E-10	-9.29	0.800	6.31

Table S8 *D-r* analysis from ^{1}H DOSY NMR data of **1** with internal standards at 0.15 M concentration in [D_6]benzene

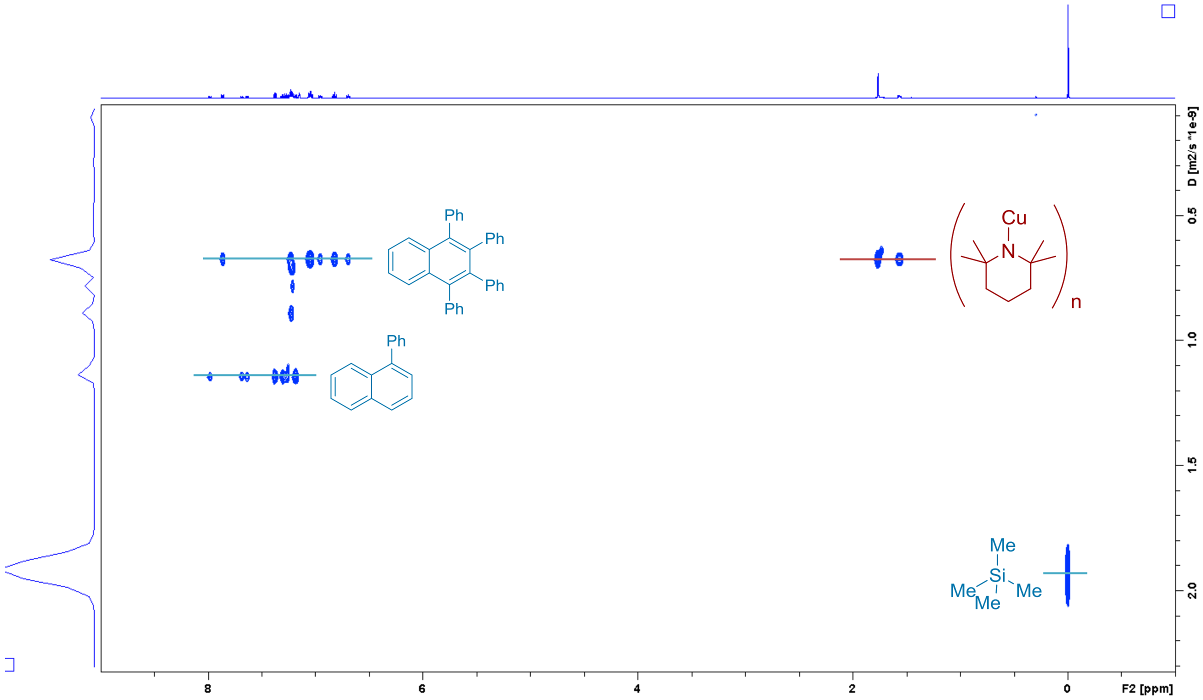

Figure S15 ^{1}H DOSY NMR spectrum of **2** with internal standards at 0.05 M concentration in [D_6]benzene

Compounds	r_{calc} / Å	Log(r_{calc} / Å)	D_{obs} / m^2s^{-1}	Log(D_{obs} / m^2s^{-1})
1,2,3,4-tetraphenylnaphthlene	5.81	0.764	6.77E-10	-9.17
1-phenylnaphthalene	4.65	0.668	1.15E-09	-8.94
Tetramethylsilane	3.90	0.591	1.93E-09	-8.71

y = -2.62x - 7.18 $R^2 = 0.996$

	D_{obs} / m^2s^{-1}	Log(D_{obs} / m^2s^{-1})	Calculated Log(r_{obs} / Å)	r_{obs} / Å
Complex 2	6.77E-10	-9.17	0.761	5.77

Table S9 *D*-*r* analysis from ^{1}H DOSY NMR data of **2** with internal standards at 0.05 M concentration in [D_6]benzene

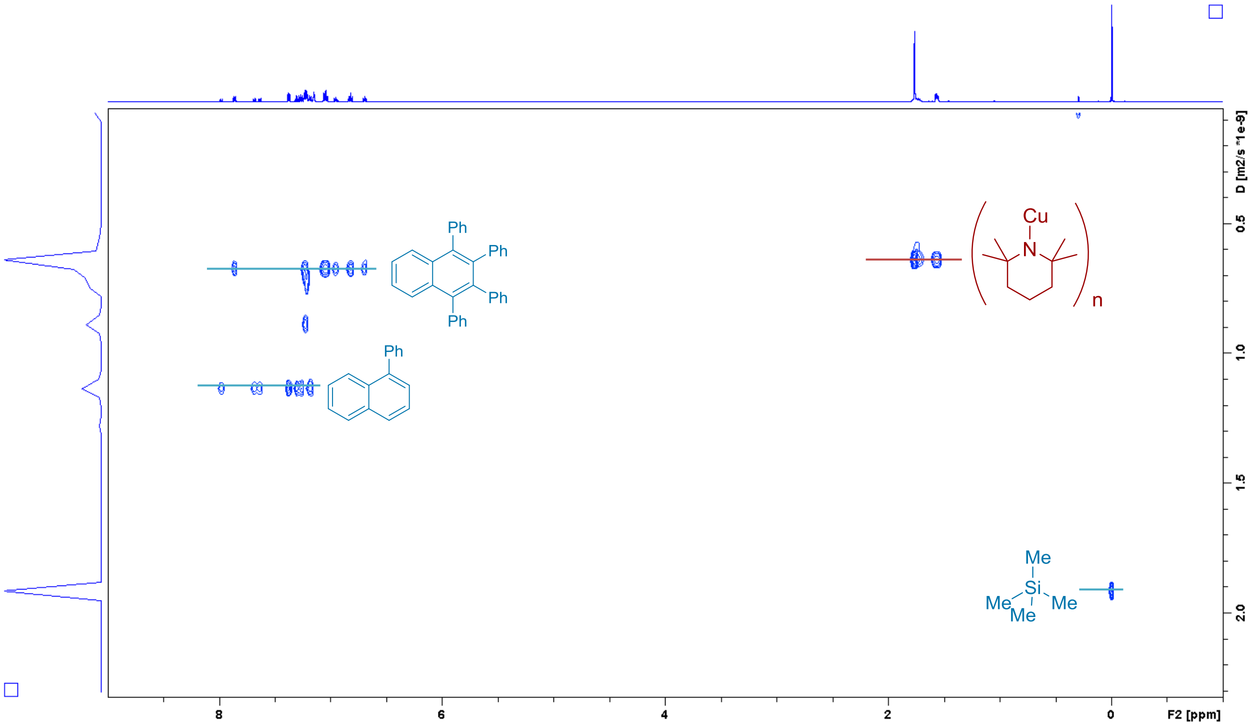

Figure S16 ^{1}H DOSY NMR spectrum of **2** with internal standards at 0.10 M concentration in [D_6]benzene

Compounds	r_{calc} / Å	Log(r_{calc} / Å)	D_{obs} / m^2s^{-1}	Log(D_{obs} / m^2s^{-1})
1,2,3,4-tetraphenylnaphthlene	5.81	0.764	6.76E-10	-9.17
1-phenylnaphthalene	4.65	0.668	1.14E-09	-8.94
Tetramethylsilane	3.90	0.591	1.92E-09	-8.72

y = -2.60x - 7.19 R^2 = 0.996

	D_{obs} / m^2s^{-1}	Log(D_{obs} / m^2s^{-1})	Calculated Log(r_{obs} / Å)	r_{obs} / Å
Complex 2	6.41E-10	-9.19	0.770	5.89

Table S10 *D*-*r* analysis from ^{1}H DOSY NMR data of **2** with internal standards at 0.10 M concentration in [D_6]benzene

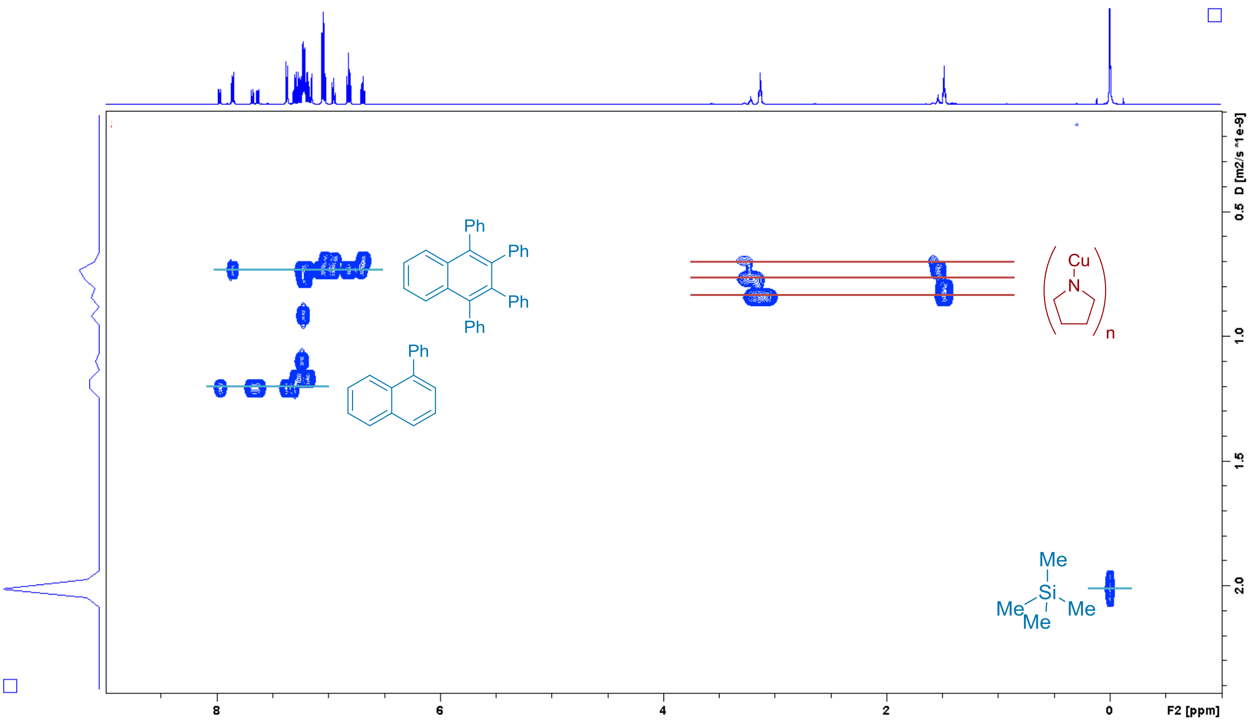

Figure S17 ^{1}H DOSY NMR spectrum of **3** with internal standards at 0.05 M concentration in [D$_6$]benzene

Compounds	r_{calc} / Å	Log(r_{calc} / Å)	D_{obs} / m^{2}s^{-1}	Log(D_{obs} / m^{2}s^{-1})
1,2,3,4-tetraphenylnaphthlene	5.81	0.764	7.36E-10	-9.13
1-phenylnaphthalene	4.65	0.668	1.21E-09	-8.92
Tetramethylsilane	3.90	0.591	2.01E-09	-8.70

$y = -2.51x - 7.22$ $R^2 = 0.995$

	D_{obs} / m^{2}s^{-1}	Log(D_{obs} / m^{2}s^{-1})	Calculated Log(r_{obs} / Å)	r_{obs} / Å
	8.45E-10	-9.07	0.737	5.45
Complex 3	7.72E-10	-9.11	0.752	5.65
	7.00E-10	-9.16	0.769	5.88

Table S11 *D*-*r* analysis from ^{1}H DOSY NMR data of **3** with internal standards at 0.05 M concentration in [D$_6$]benzene

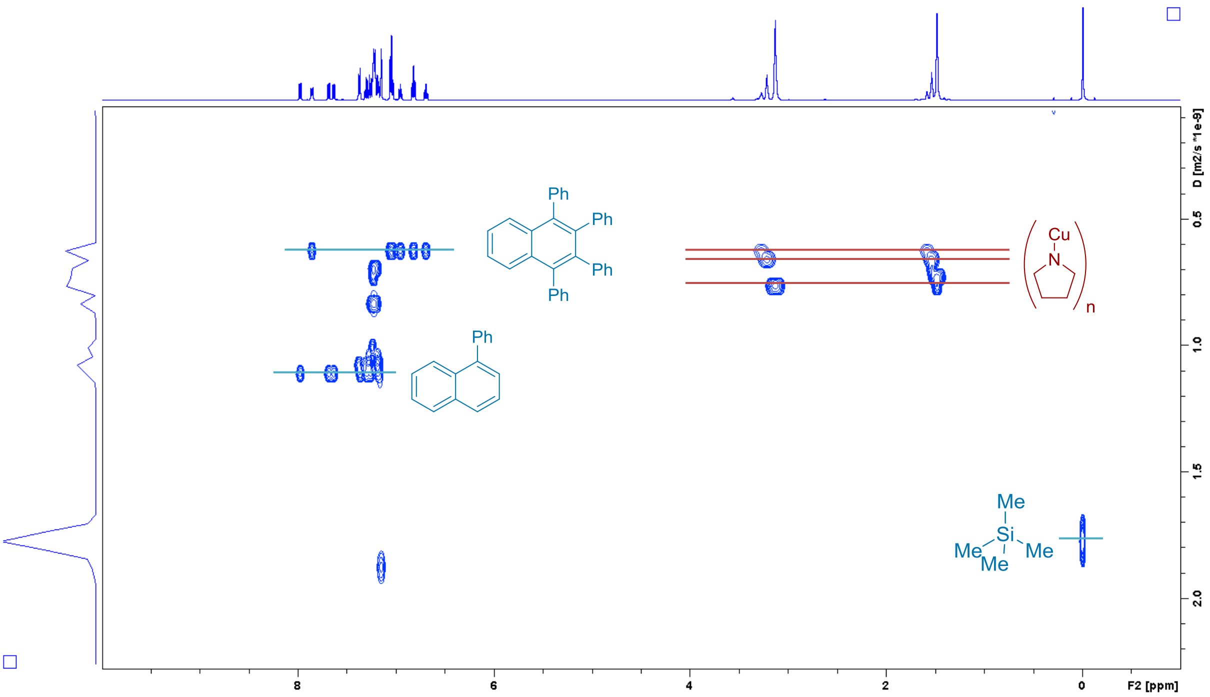

Figure S18 ^{1}H DOSY NMR spectrum of **3** with internal standards at 0.20 M concentration in $[D_6]$benzene

Compounds	r_{calc} / Å	Log(r_{calc} / Å)	D_{obs} / m^2s^{-1}	Log(D_{obs} / m^2s^{-1})
1,2,3,4-tetraphenylnaphthlene	5.81	0.764	6.28E-10	-9.20
1-phenylnaphthalene	4.65	0.668	1.11E-09	-8.95
Tetramethylsilane	3.90	0.591	1.77E-09	-8.75

$y = -2.60x - 7.21$ $R^2 = 1.000$

	D_{obs} / m^2s^{-1}	Log(D_{obs} / m^2s^{-1})	Calculated Log(r_{obs} / Å)	r_{obs} / Å
Complex 3	7.67E-10	-9.12	0.730	5.38
	6.63E-10	-9.18	0.755	5.68
	6.28E-10	-9.20	0.764	5.80

Table S12 *D*-*r* analysis from ^{1}H DOSY NMR data of **3** with internal standards at 0.20 M concentration in $[D_6]$benzene

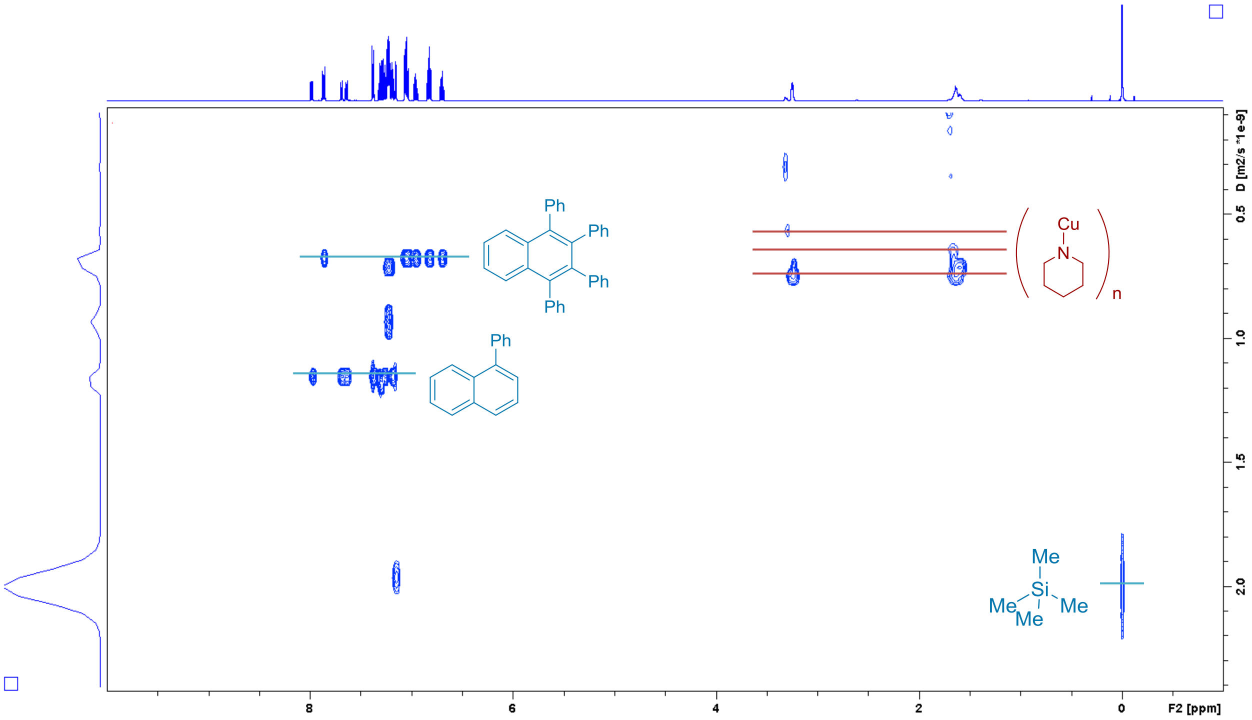

Figure S19 ^{1}H DOSY NMR spectrum of **4** with internal standards at 0.05 M concentration in [D_6]benzene

Compounds	r_{calc} / Å	Log(r_{calc} / Å)	D_{obs} / m^2s^{-1}	Log(D_{obs} / m^2s^{-1})
1,2,3,4-tetraphenylnaphthlene	5.81	0.764	6.79E-10	-9.17
1-phenylnaphthalene	4.65	0.668	1.16E-09	-8.94
Tetramethylsilane	3.90	0.591	2.00E-09	-8.70

y = -2.70x - 7.12 R^2 = 0.995

	D_{obs} / m^2s^{-1}	Log(D_{obs} / m^2s^{-1})	Calculated Log(r_{obs} / Å)	r_{obs} / Å
Complex 4	7.52E-10	-9.12	0.744	5.55
	6.41E-10	-9.19	0.770	5.89
	5.67E-10	-9.25	0.790	6.16

Table S13 *D*-*r* analysis from ^{1}H DOSY NMR data of **4** with internal standards at 0.05 M concentration in [D_6]benzene

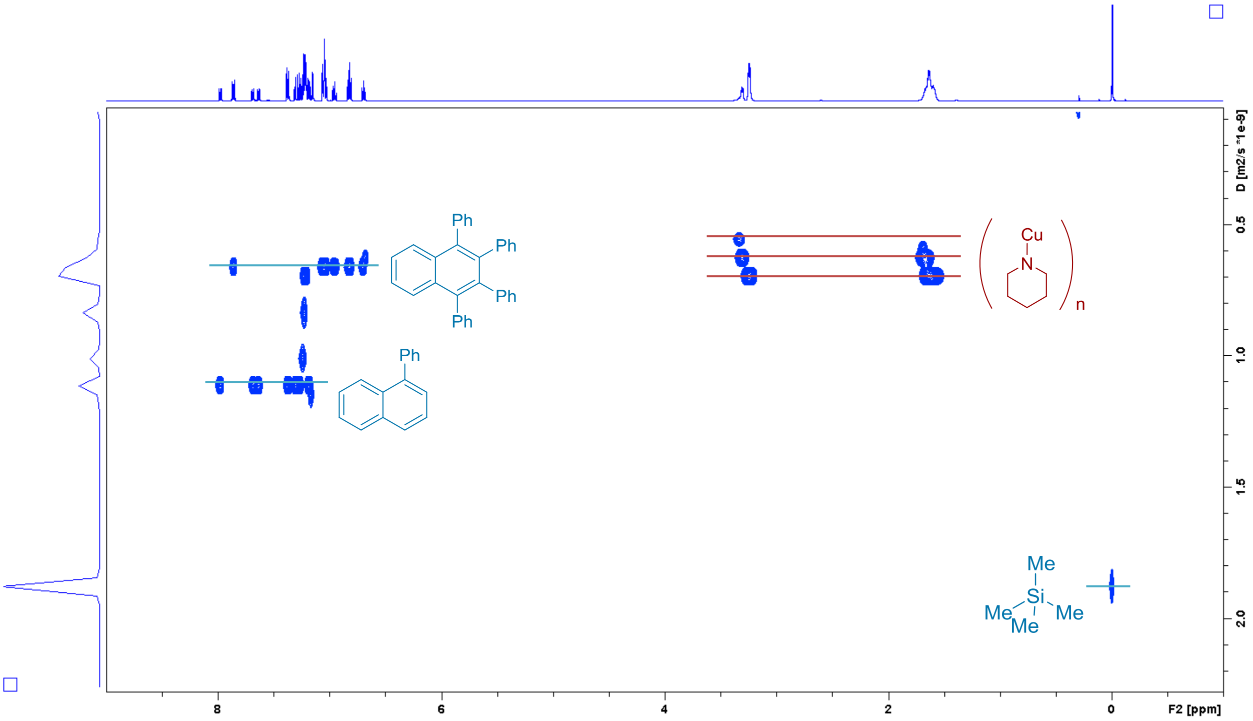

Figure S20 ^{1}H DOSY NMR spectrum of **4** with internal standards at 0.20 M concentration in [D$_6$]benzene

Compounds	r_{calc} / Å	Log(r_{calc} / Å)	D_{obs} / m^{2}s^{-1}	Log(D_{obs} / m^{2}s^{-1})
1,2,3,4-tetraphenylnaphthlene	5.81	0.764	6.63E-10	-9.18
1-phenylnaphthalene	4.65	0.668	1.12E-09	-8.95
Tetramethylsilane	3.90	0.591	1.88E-09	-8.73

y = -2.60x - 7.20 R^2 = 0.996

	D_{obs} / m^{2}s^{-1}	Log(D_{obs} / m^{2}s^{-1})	Calculated Log(r_{obs} / Å)	r_{obs} / Å
Complex 4	6.98E-10	-9.16	0.752	5.65
	6.29E-10	-9.20	0.770	5.89
	5.59E-10	-9.25	0.789	6.16

Table S14 *D*-*r* analysis from ^{1}H DOSY NMR data of **4** with internal standards at 0.20 M concentration in [D$_6$]benzene

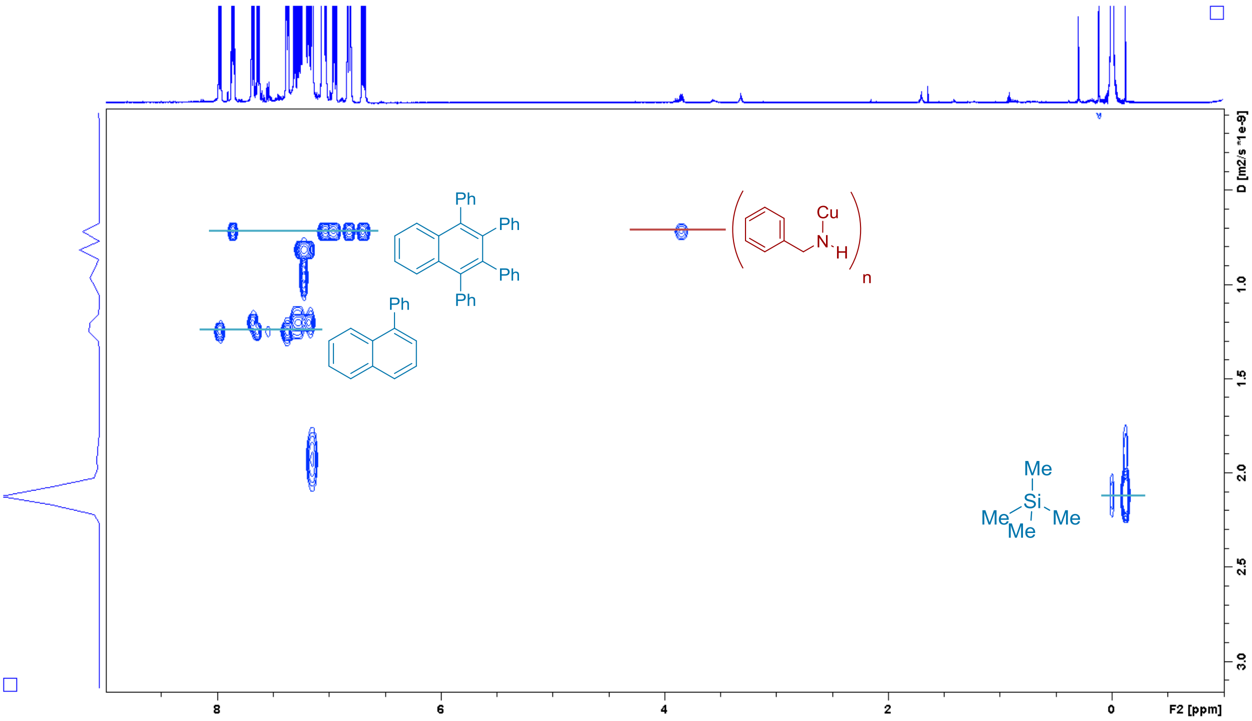

Figure S21 ^{1}H DOSY NMR spectrum of **5** with internal standards at 0.01 M concentration in [D$_6$]benzene

Compounds	r_{calc} / Å	Log(r_{calc} / Å)	D_{obs} / m^2s^{-1}	Log(D_{obs} / m^2s^{-1})
1,2,3,4-tetraphenylnaphthlene	5.81	0.764	7.22E-10	-9.14
1-phenylnaphthalene	4.65	0.668	1.25E-09	-8.90
Tetramethylsilane	3.90	0.591	2.12E-09	-8.67

y = -2.70x - 7.09 R^2 = 0.997

	D_{obs} / m^2s^{-1}	Log(D_{obs} / m^2s^{-1})	Calculated Log(r_{obs} / Å)	r_{obs} / Å
Complex 5	7.22E-10	-9.14	0.762	5.78

Table S15 *D-r* analysis from ^{1}H DOSY NMR data of **5** with internal standards at 0.01 M concentration in [D$_6$]benzene

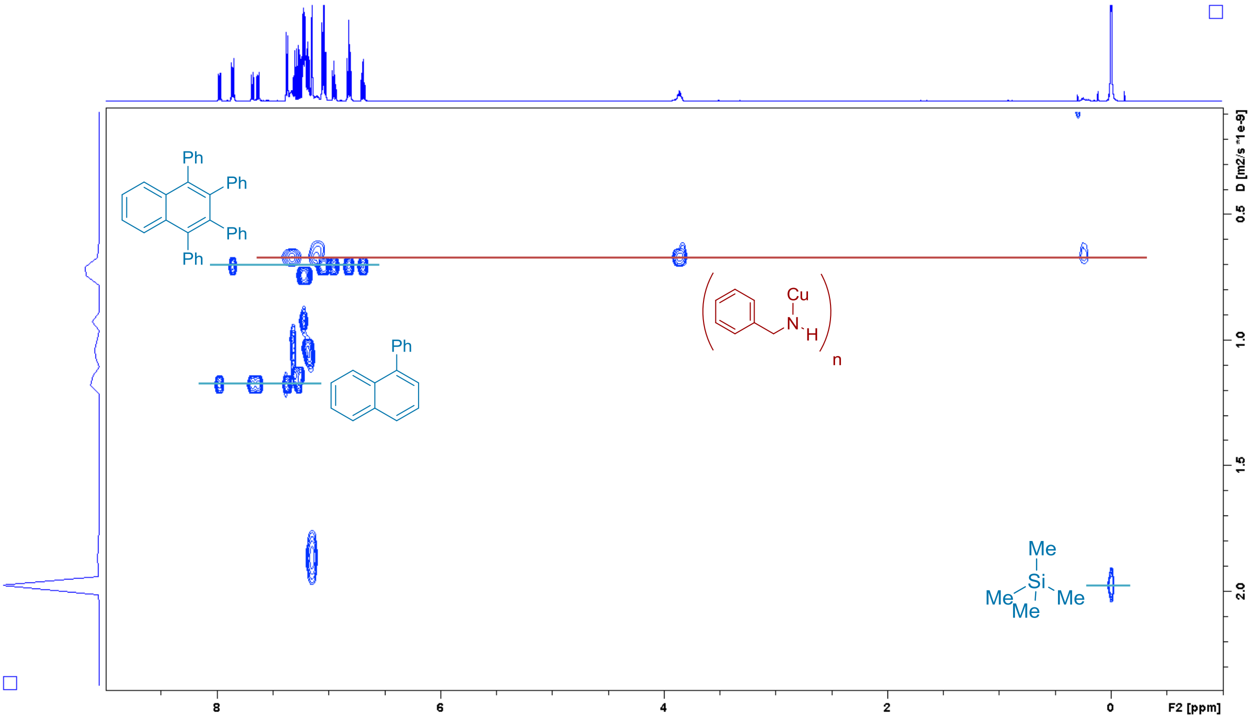

Figure S22 ^{1}H DOSY NMR spectrum of **5** with internal standards at 0.05 M concentration in [D_6]benzene

Compounds	r_{calc} / Å	Log(r_{calc} / Å)	D_{obs} / m^2s^{-1}	Log(D_{obs} / m^2s^{-1})
1,2,3,4-tetraphenylnaphthlene	5.81	0.764	7.08E-10	-9.15
1-phenylnaphthalene	4.65	0.668	1.18E-09	-8.93
Tetramethylsilane	3.90	0.591	1.98E-09	-8.70

$y = -2.56x - 7.20$ $R^2 = 0.995$

	D_{obs} / m^2s^{-1}	Log(D_{obs} / m^2s^{-1})	Calculated Log(r_{obs} / Å)	r_{obs} / Å
Complex 5	6.72E-10	-9.17	0.770	5.88

Table S16 *D*-*r* analysis from ^{1}H DOSY NMR data of **5** with internal standards at 0.05 M concentration in [D_6]benzene

5 ^{1}H NMR data of 3 and 4 at 0.05 and 0.20 M concentration in [D$_6$]benzene

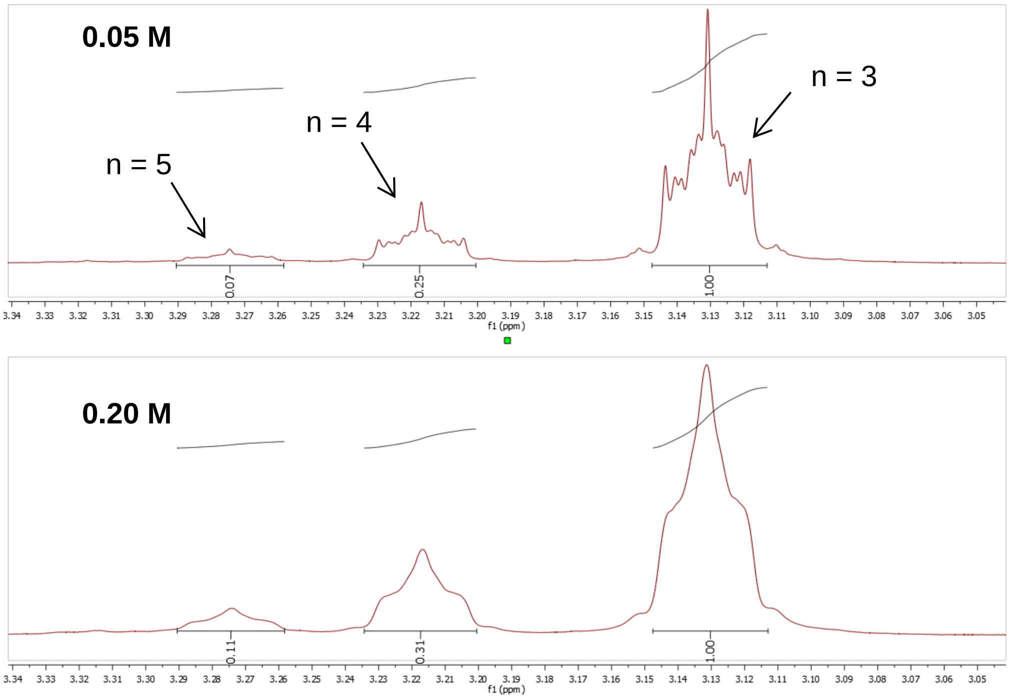

Figure S23 Cropped ^{1}H NMR spectra of **3** at 0.05 and 0.20 M concentration in [D$_6$]benzene comparing the integrals for the α-CH_2 protons' resonances of the trimeric (n = 3), tetrameric (n = 4) and pentameric (n = 5) aggregates

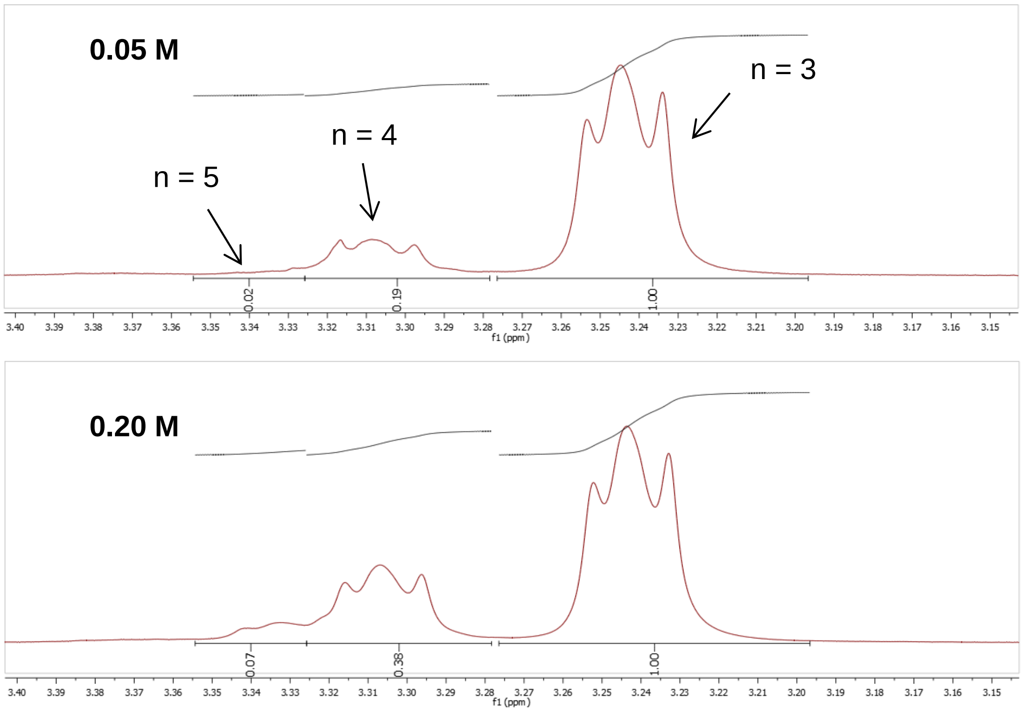

Figure S24 Cropped ^{1}H NMR spectra of **4** at 0.05 and 0.20 M concentration in [D$_6$]benzene comparing the integrals for the α-CH_2 protons' resonances of the trimeric (n = 3), tetrameric (n = 4) and pentameric (n = 5) aggregates

The integrations of the tetramer and pentamer resonances increased with respect to the integration of the trimer resonances at higher concentrations in the ^{1}H NMR spectra of **3** and **4** from 0.05 M to 0.20 M, which confirmed there were equilibria between these aggregates.

6 ^{1}H DOSY NMR data of 3 and 4 in the presence of 1,10-phenanthroline and internal standards

The following ^{1}H DOSY NMR spectra were obtained using a Bruker AV-500 spectrometer at 500 MHz and 25°C.

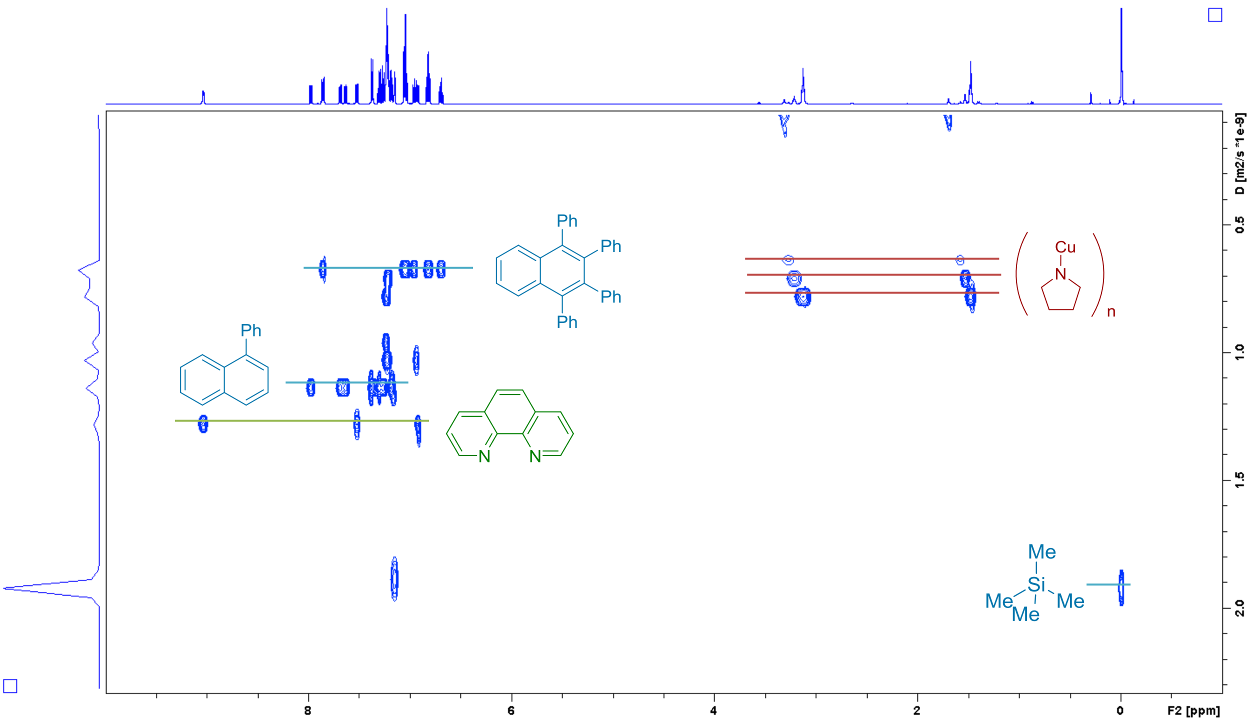

Figure S25 ^{1}H DOSY NMR spectrum of **3** with one equivalent of 1,10-phenanthroline and internal standards in [D$_6$]benzene at 0.05 M concentration

Compounds	r_{calc} / Å	Log(r_{calc} / Å)	D_{obs} / m^{2}s^{-1}	Log(D_{obs} / m^{2}s^{-1})
1,2,3,4-tetraphenylnaphthlene	5.81	0.764	6.76E-10	-9.17
1-phenylnaphthalene	4.65	0.668	1.14E-09	-8.94
Tetramethylsilane	3.90	0.591	1.92E-09	-8.72
1,10-phenanthroline	4.40	-	-	-

y = -2.61x - 7.18 R^2 = 0.996

	D_{obs} / m^{2}s^{-1}	Log(D_{obs} / m^{2}s^{-1})	Calculated Log(r_{obs} / Å)	r_{obs} / Å
Complex 3	7.83E-10	-9.11	0.737	5.45
Complex 3	7.12E-10	-9.15	0.752	5.65
Complex 3	6.41E-10	-9.19	0.770	5.89
1,10-phenanthroline	1.28E-09	-8.89	0.655	4.51

Table S17 *D-r* analysis from ^{1}H DOSY NMR data of **3** with one equivalent of 1,10-phenanthroline and internal standards in [D$_6$]benzene at 0.05 M concentration

^1H DOSY NMR analysis gave observed radii (r_{obs}) values that were similar with the expected aggregates for complex **3** and of free 1,10-phenanthroline.

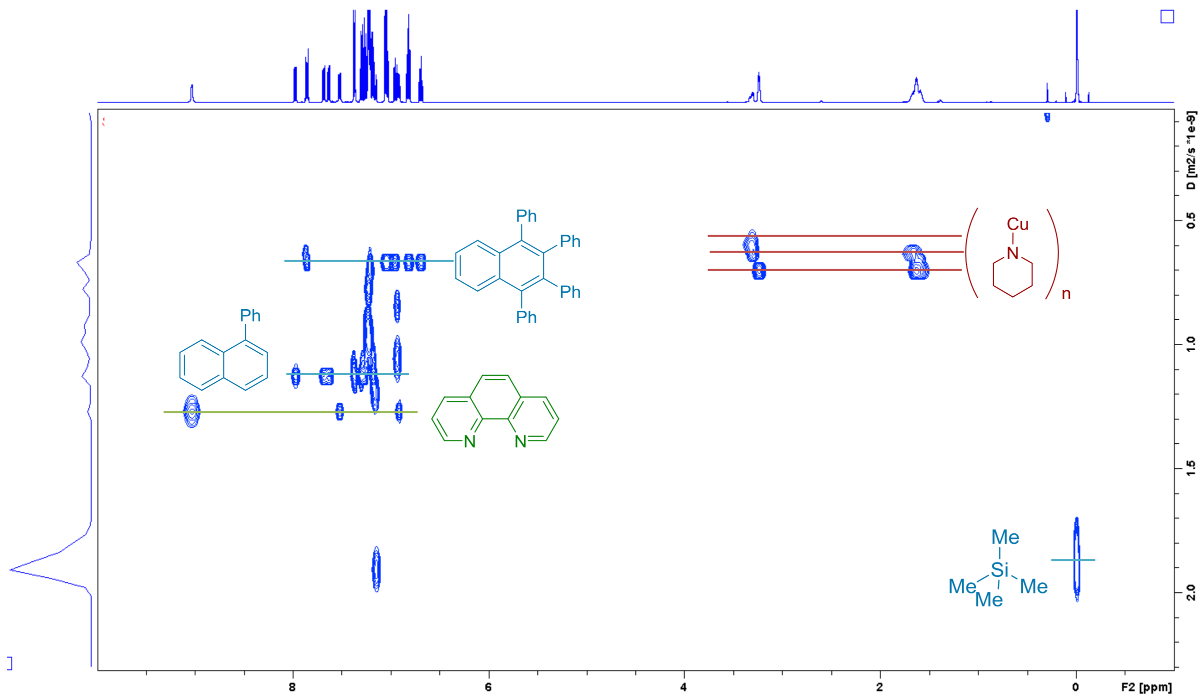

Figure S26 ^1H DOSY NMR spectrum of **4** with one equivalent of 1,10-phenanthroline and internal standards in [D$_6$]benzene at 0.05 M concentration

Compounds	r_{calc} / Å	Log(r_{calc} / Å)	D_{obs} / m^2s^{-1}	Log(D_{obs} / m^2s^{-1})
1,2,3,4-tetraphenylnaphthlene	5.81	0.764	6.70E-10	-9.17
1-phenylnaphthalene	4.65	0.668	1.13E-09	-8.95
Tetramethylsilane	3.90	0.591	1.91E-09	-8.72
1,10-phenanthroline	4.40	-	-	-

$y = -2.61x - 7.19$ $R^2 = 0.996$

	D_{obs} / m^2s^{-1}	Log(D_{obs} / m^2s^{-1})	Calculated Log(r_{obs} / Å)	r_{obs} / Å
Complex 4	7.06E-10	-9.15	0.752	5.65
Complex 4	6.34E-10	-9.20	0.770	5.89
Complex 4	5.64E-10	-9.25	0.790	6.16
1,10-phenanthroline	1.27E-09	-8.90	0.654	4.51

Table S18 *D*-*r* analysis from ^1H DOSY NMR data of 4 with one equivalent of 1,10-phenanthroline and internal standards in [D$_6$]benzene at 0.05 M concentration

^1H DOSY NMR analysis gave observed radii values that were similar with the expected aggregates for complex **4** and of free 1,10-phenanthroline.

7 NMR data of 3 and 4 in the presence of 1,10-phenanthroline in [D$_6$]DMSO

The following ^{1}H and ^{13}C NMR spectra were obtained using Bruker AV-400 spectrometers at 400 MHz and 23 °C. ^{1}H-^{1}H ROESY NMR spectrum was obtained using a Bruker DRX400 or AV-500 spectrometer at 400 and 500 MHz respectively at 23 °C.

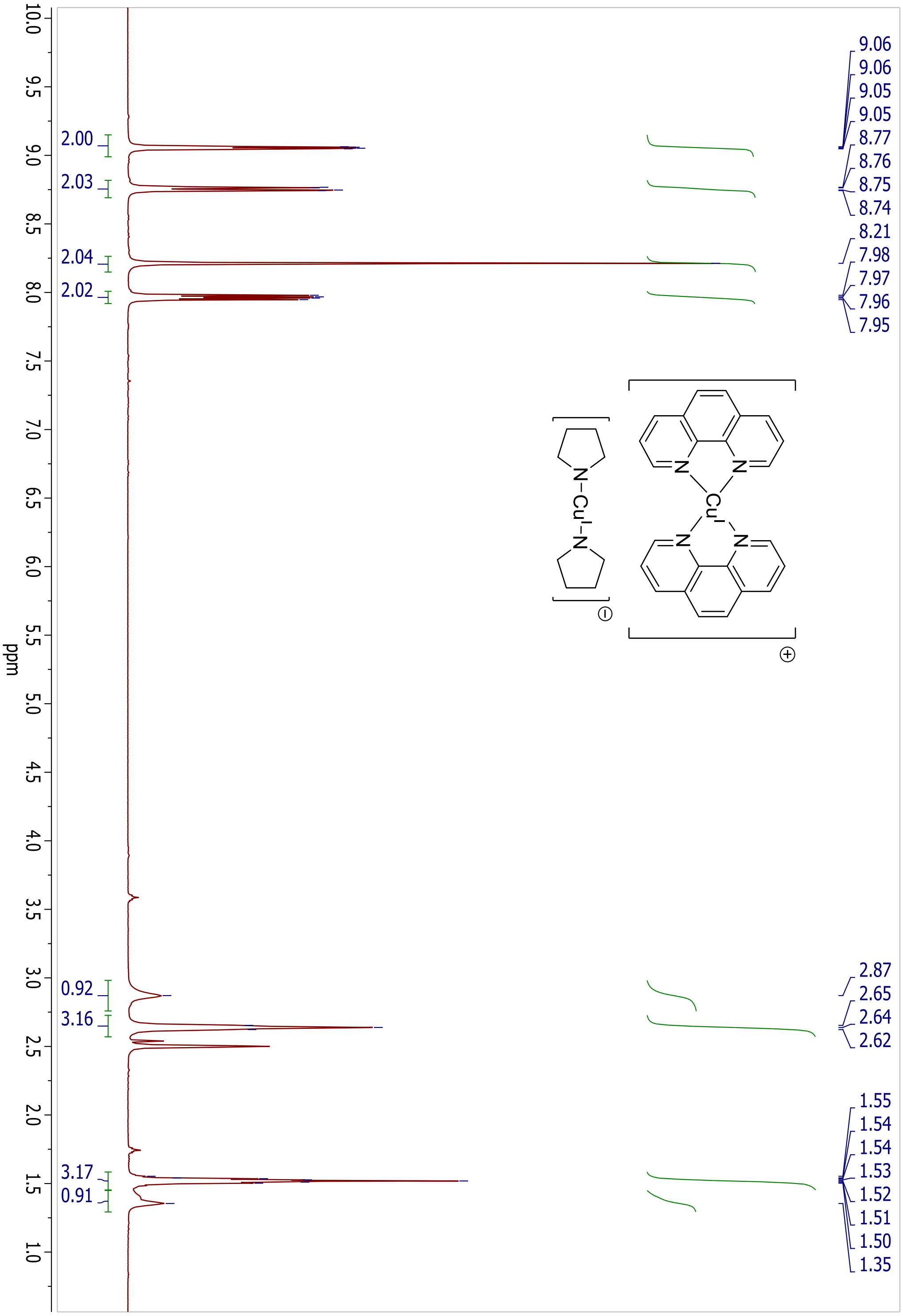

Figure S27 ^{1}H NMR spectrum of **3** with one equivalent of 1,10-phenanthroline in [D$_6$]DMSO

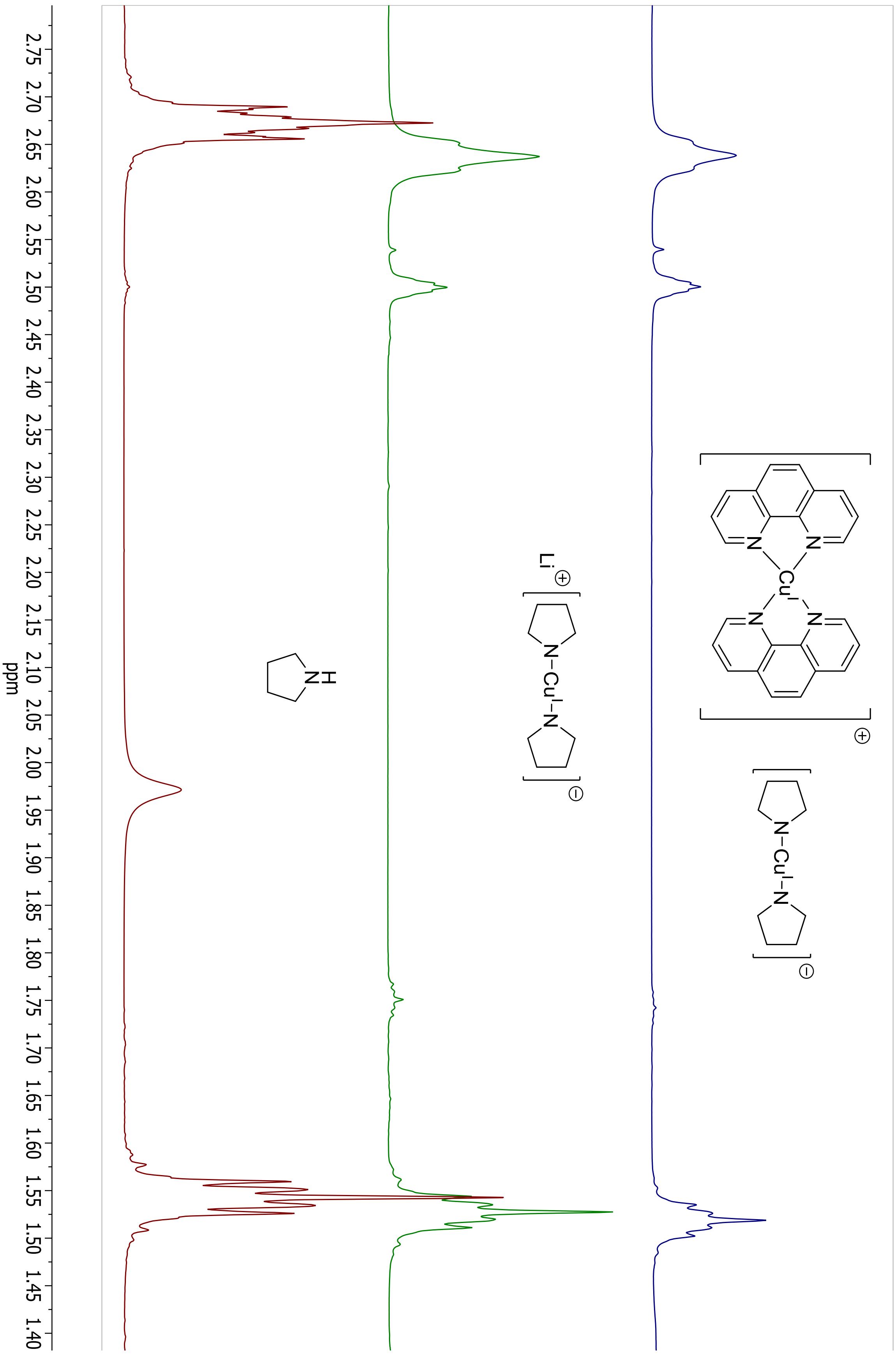

Figure S28 Comparison of ^{1}H NMR spectra of **3** with one equivalent of 1,10-phenanthroline, lithium di(pyrrolidide)cuprate and pyrrolidine in [D$_6$]DMSO

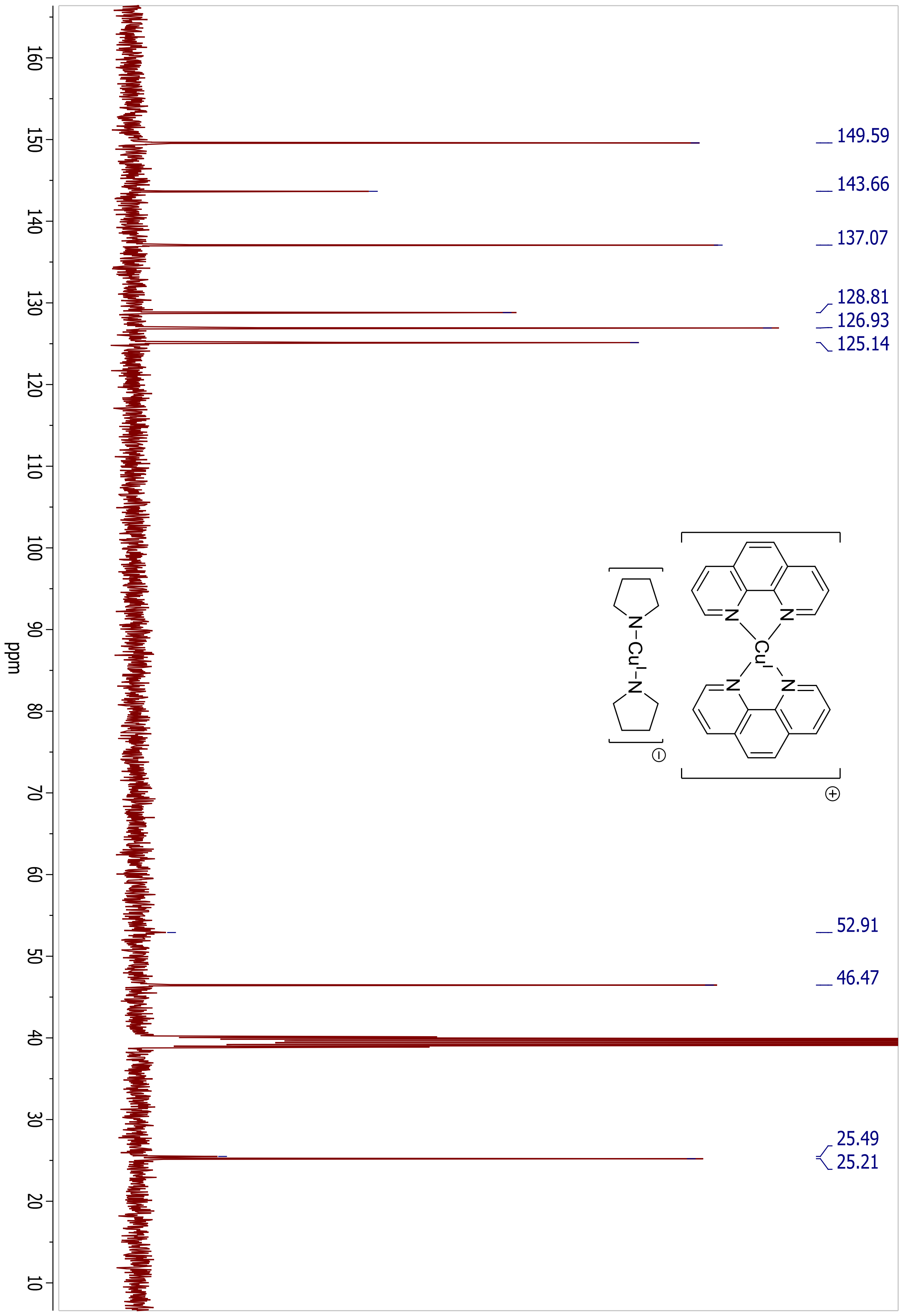

Figure S29 ^{13}C NMR spectrum of **3** with one equivalent of 1,10-phenanthroline in [D$_6$]DMSO

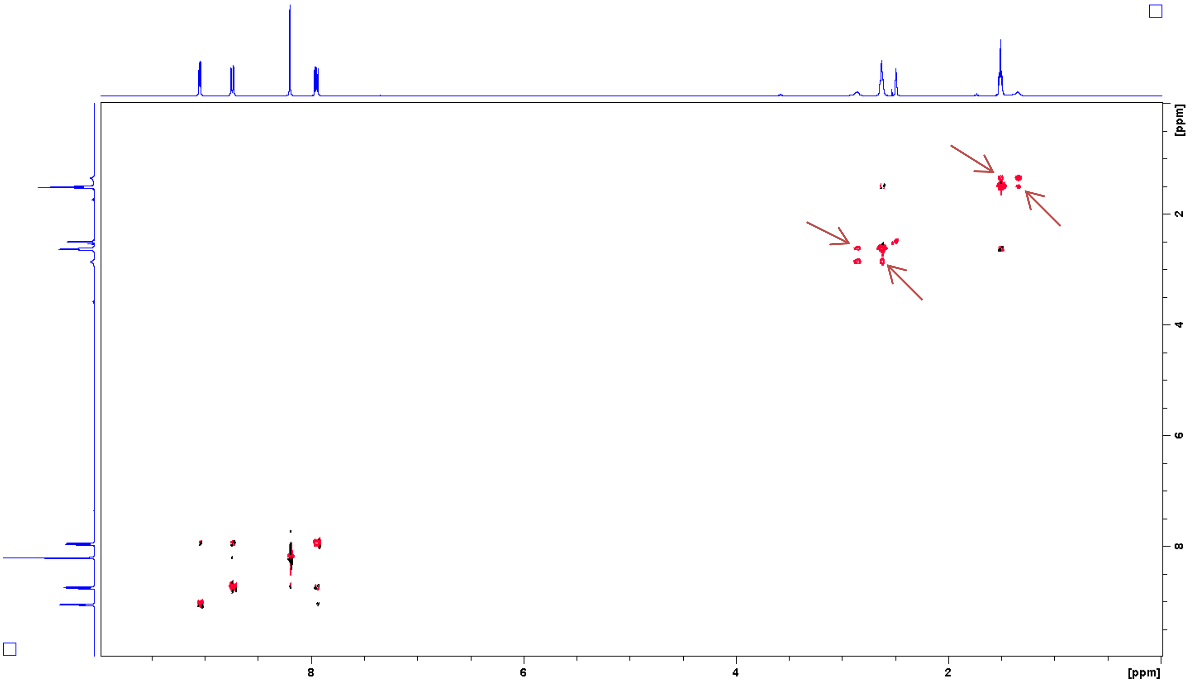

Figure S30 ^{1}H-^{1}H ROESY NMR spectrum of **3** with one equivalent of 1,10-phenanthroline in [D_6]DMSO. Positive- and negative-phase are black and red colour respectively. Negative-phase cross peaks involving the α-protons at 2.64 ppm and the α-protons of another $[NR_2]^-$ moiety at 2.85 ppm and also involving the β-protons at 1.52 ppm and the β-protons of another $[NR_2]^-$ moiety at 1.34 ppm are indicated by red arrows.

In the ^{1}H-^{1}H ROESY NMR spectrum, the diagonal is in the negative-phase. No cross peaks involving the pyrrolidide and phen ligands' protons are present, which supported the presence of a double salt structure in [D_6]DMSO solution. In addition, negative-phase cross peaks between the α- and β-protons of the $[Cu(pyrrolidide)_2]^-$ anion and the α- and β-protons of another pyrrolidide moiety with similar chemical shifts indicated there is chemical exchange with another species that is likely to be $[Cu_4(pyrrolidide)_5]^-$.

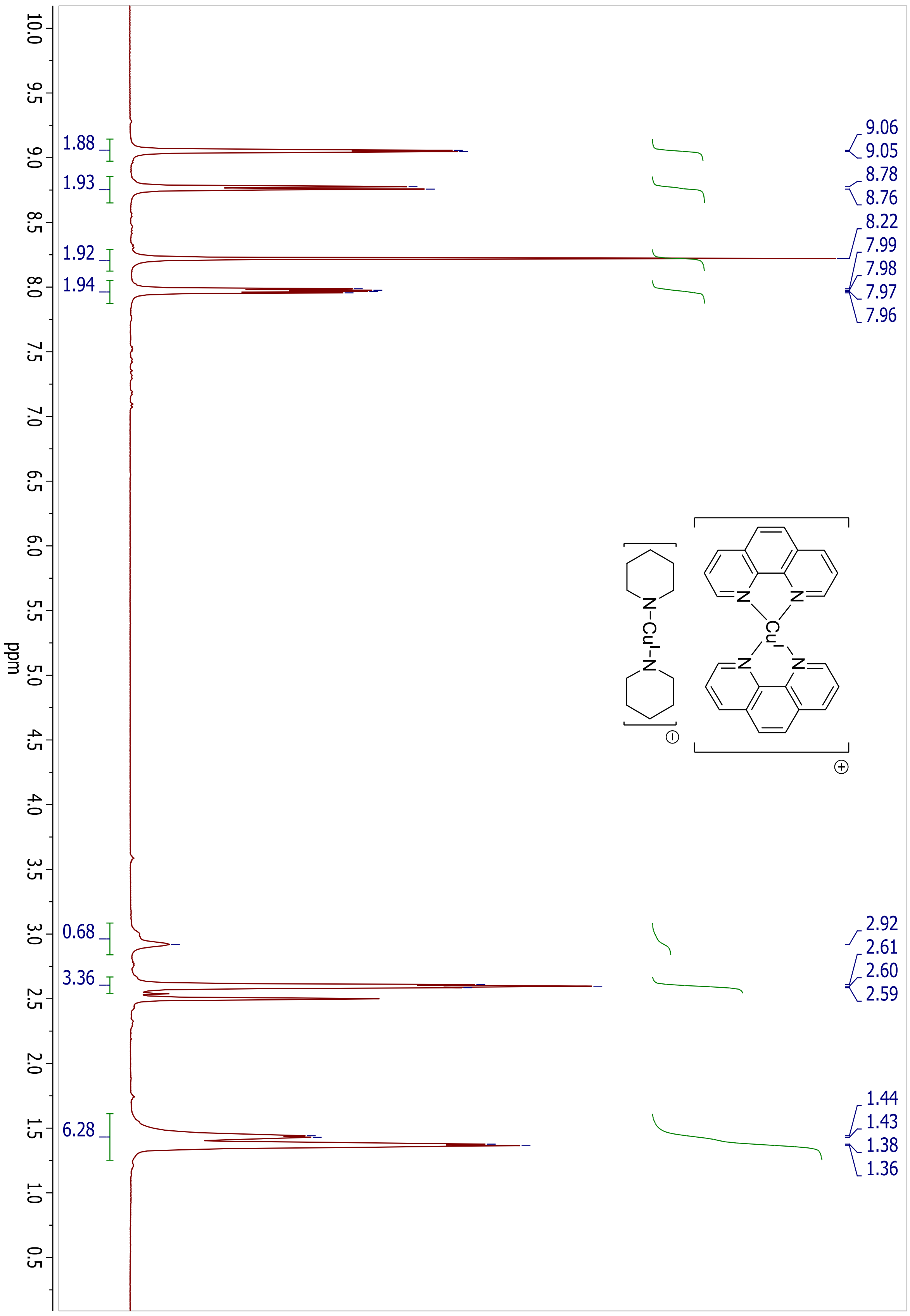

Figure S31 ^{1}H NMR spectrum of **4** with one equivalent of 1,10-phenanthroline in [D$_6$]DMSO

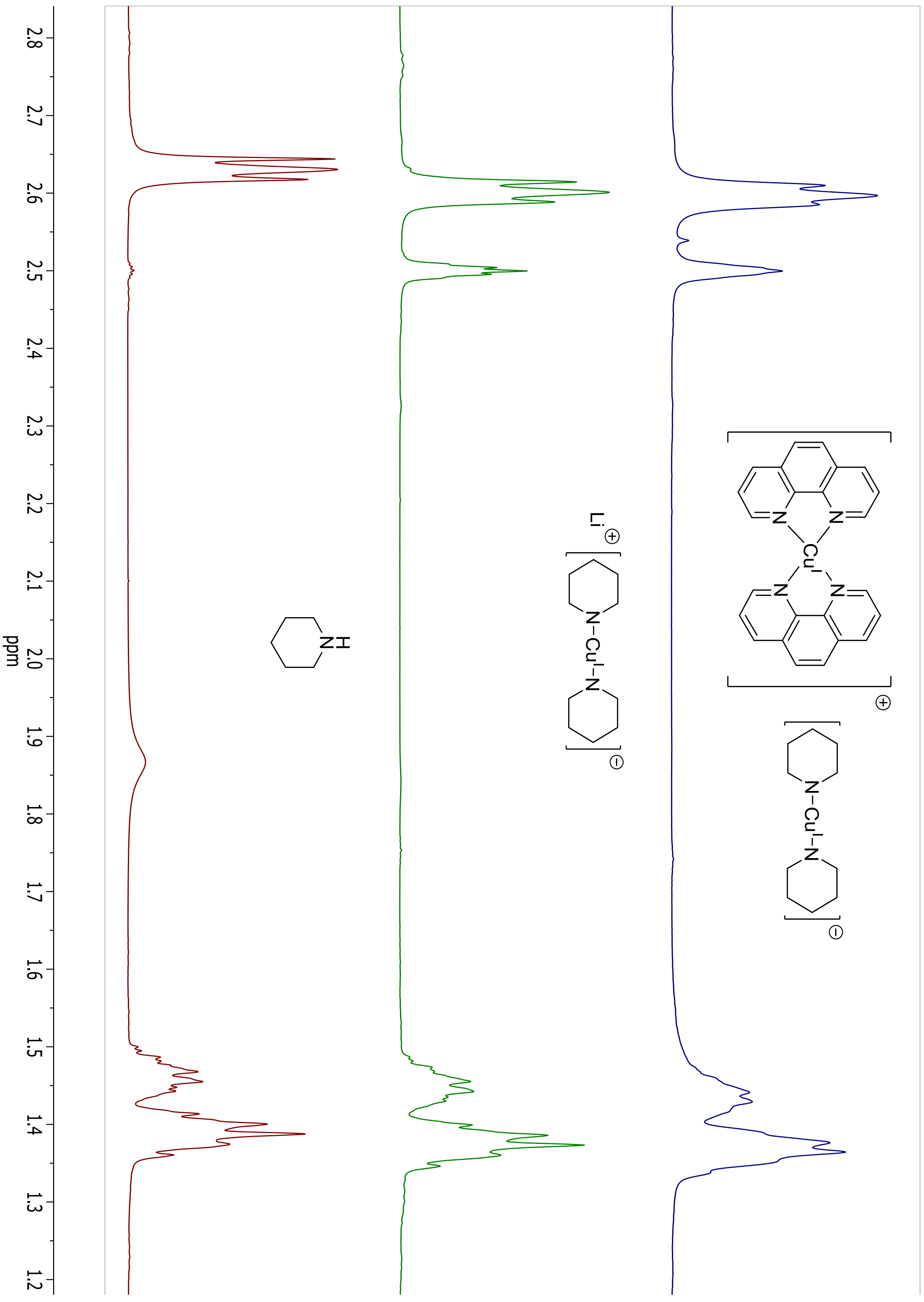

Figure S32 Comparison of ^{1}H NMR spectra of **4** with one equivalent of 1,10-phenanthroline, lithium di(piperidido)cuprate and piperidine in $[D_6]$DMSO

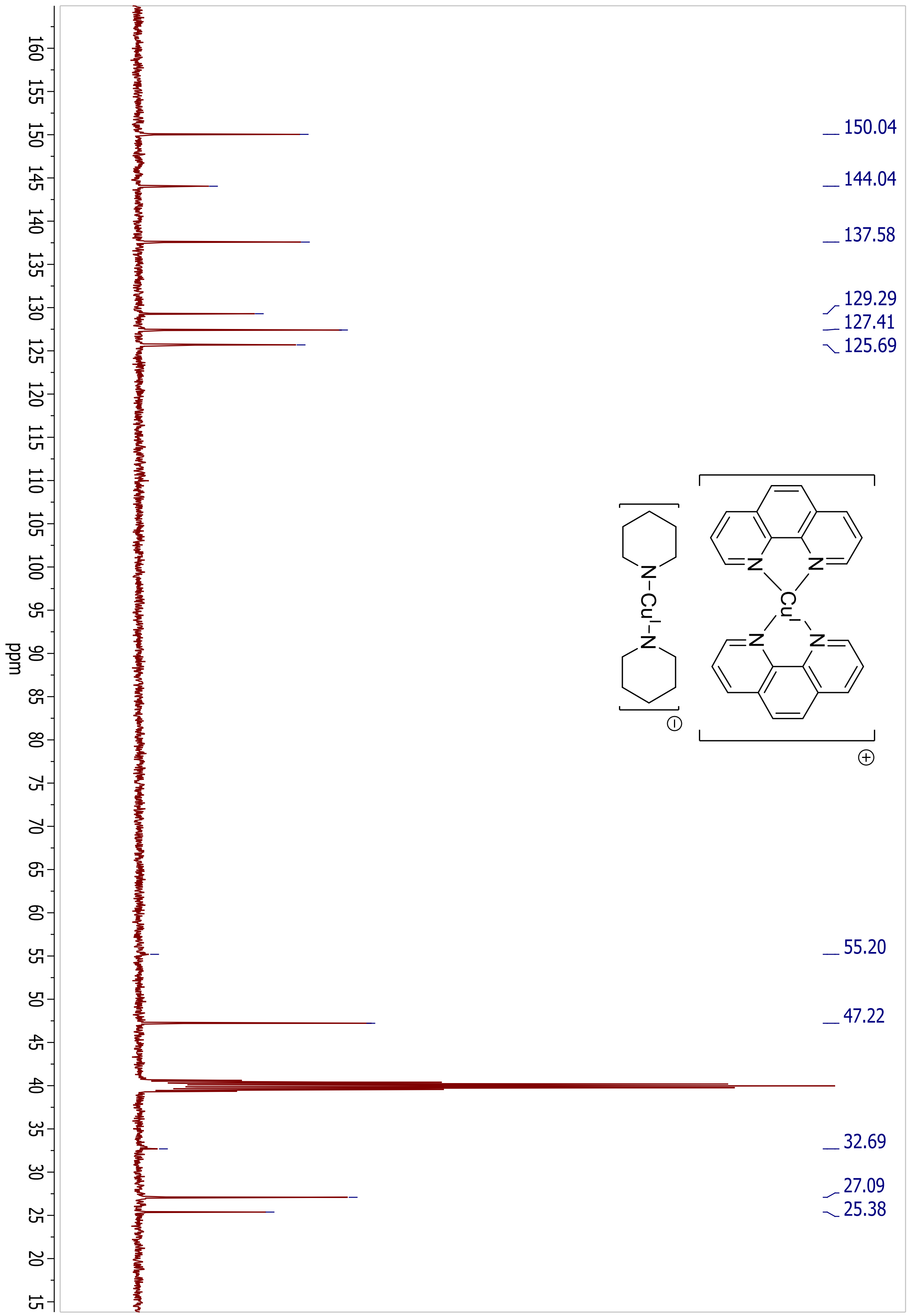

Figure S33 ^{13}C NMR spectrum of **4** with one equivalent of 1,10-phenanthroline in [D$_6$]DMSO

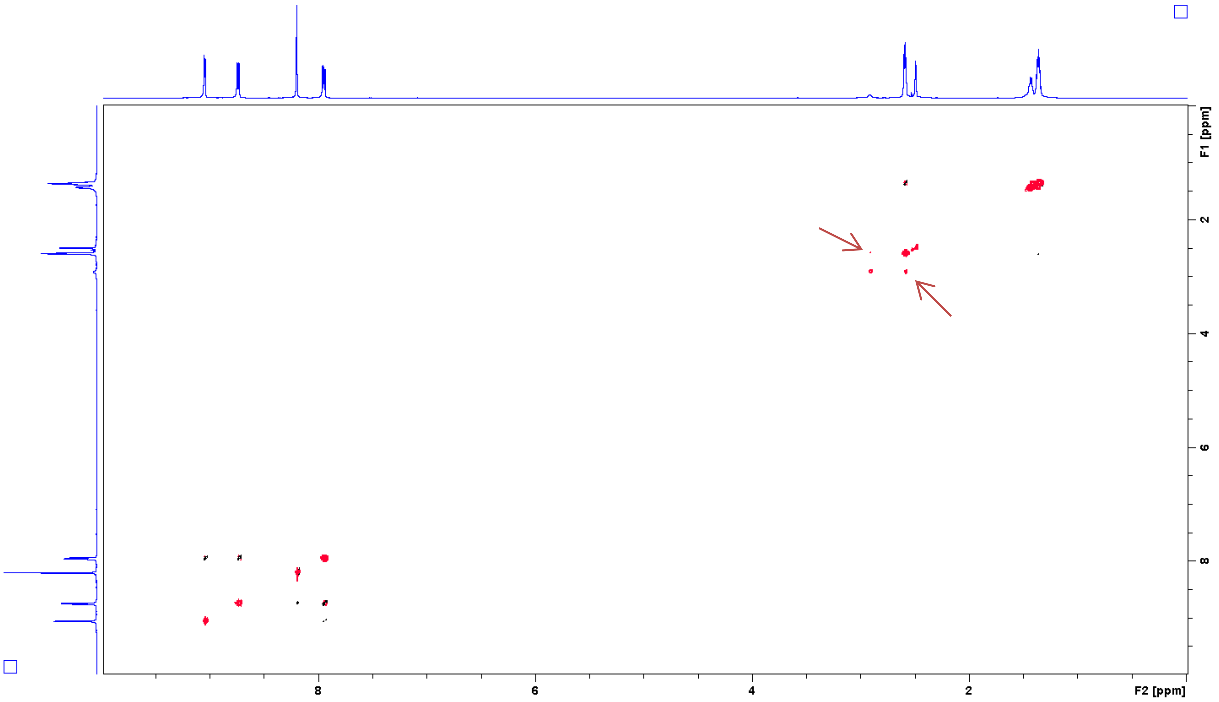

Figure S34 ^{1}H-^{1}H ROESY NMR spectrum of **4** with one equivalent of 1,10-phenanthroline in [D_6]DMSO. Positive- and negative-phase are black and red colour respectively. Negative-phase cross peaks are indicated by red arrows.

In the ^{1}H-^{1}H ROESY NMR spectrum, the diagonal is in the negative-phase. No cross peaks involving the piperidide and phen ligands' protons are present, which supported the presence of a double salt structure in [D_6]DMSO solution. In addition, negative-phase cross peaks between the α-protons of the $[Cu(piperidide)_2]^-$ anion and the α-protons of another piperidide moiety with similar chemical shifts indicated there is chemical exchange with another species, that is likely to be $[Cu_4(piperidide)_5]^-$. The chemical exchange of the β- and γ-protons of the $[Cu(piperidide)_2]^-$ anion is not visible as the resonances overlap with the other species.

8 ESI-MS data

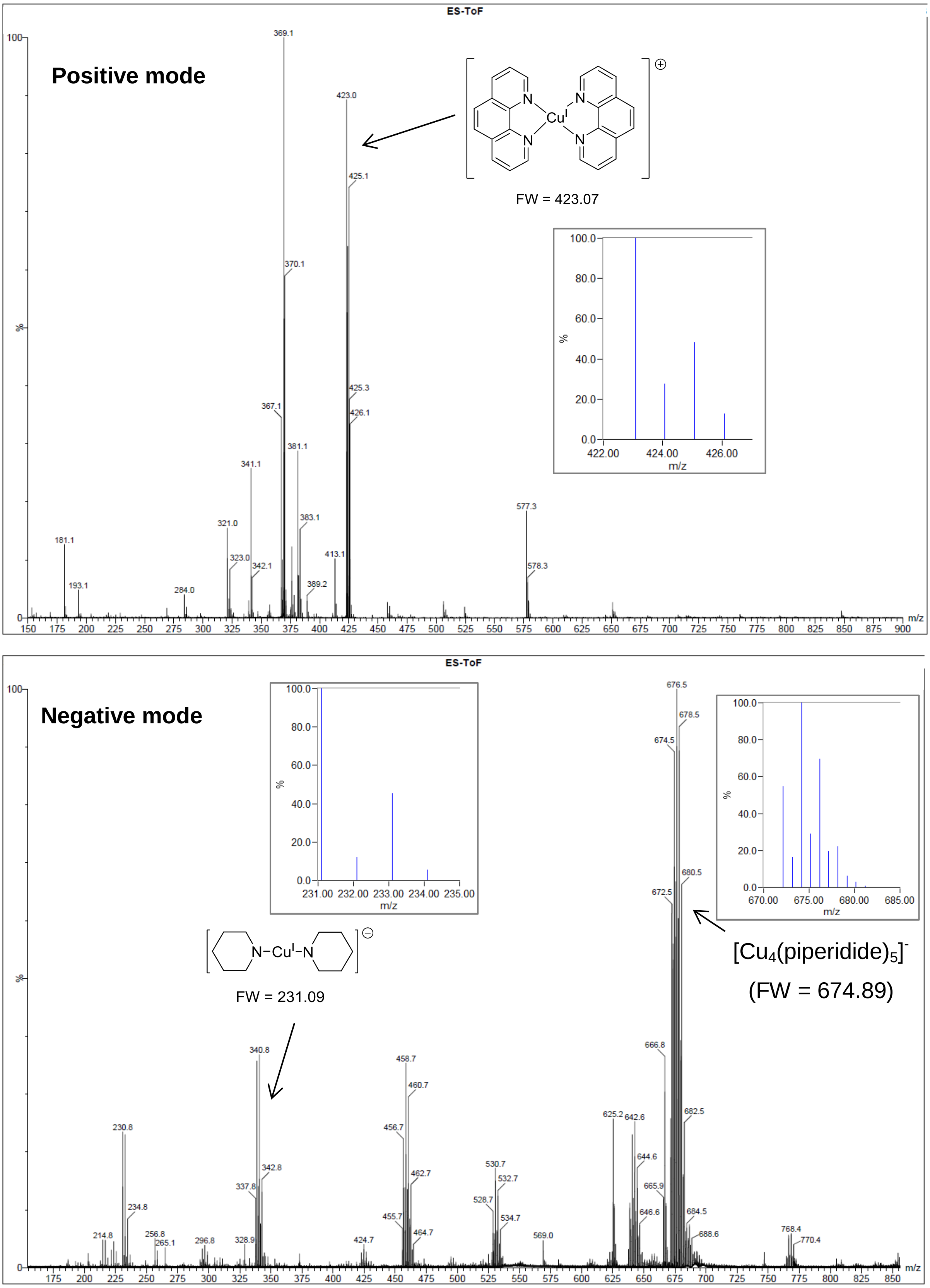

Figure S35 Positive and negative mode ESI-MS data of **4** in the presence of one equivalent of 1,10-phenanthroline in DMSO. Inset mass spectra show the expected isotope patterns for the adjacent ions

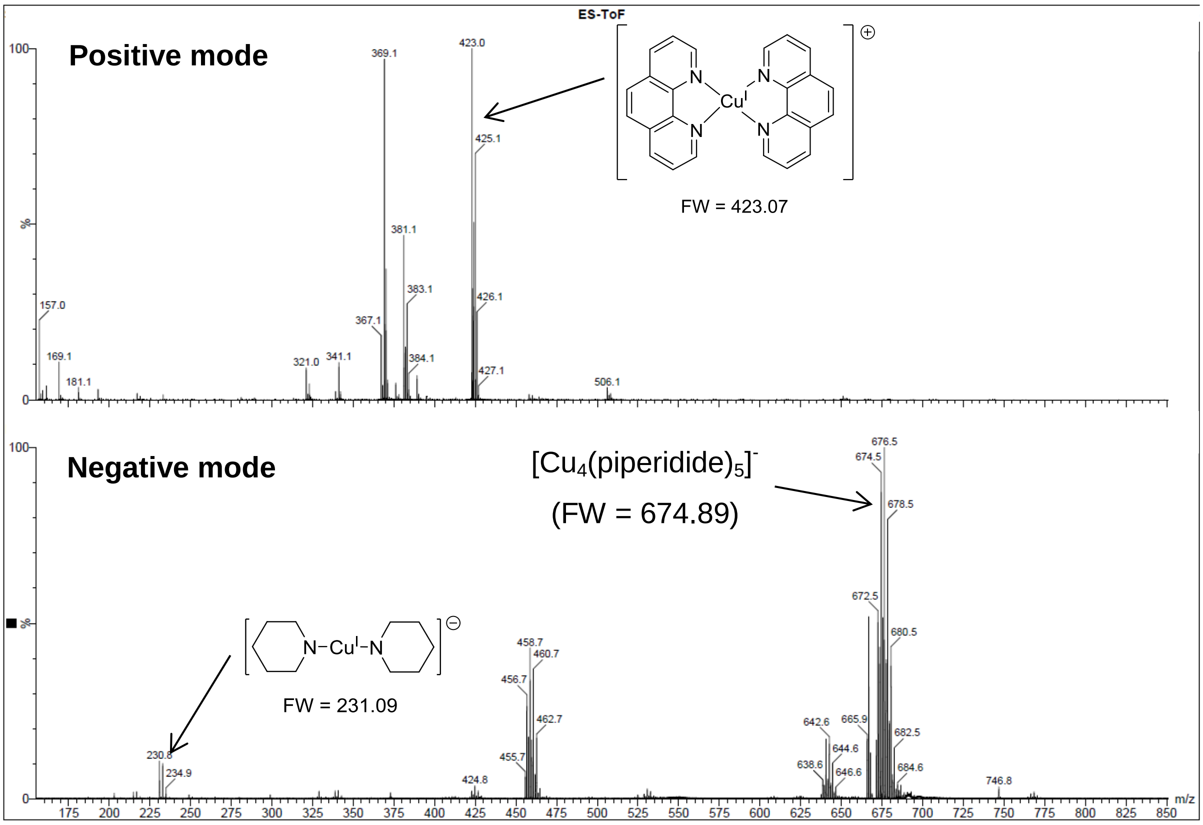

Figure S36 Positive and negative mode ESI-MS data of **4** in the presence of one equivalent of 1,10-phenanthroline in benzene.

9 X-Ray Crystallography

Data	1	2	4	6
**Formula**	$C_{48}H_{88}Cu_4N_4$	$C_{36}H_{72}Cu_4N_4$	$C_{20}H_{40}Cu_4N_4$	$C_{46}H_{66}Cu_4N_2$
**Formula weight**	975.38	815.14	590.72	901.16
**Colour, habit**	colourless blocky needles	colourless blocky needles	colourless needles	yellow blocks
**Crystal size / mm^3**	0.34 × 0.09 × 0.08	0.46 × 0.35 × 0.31	0.27 × 0.14 × 0.12	0.44 × 0.40 × 0.31
**Temperature / K**	173	173	173	173
**Crystal system**	monoclinic	monoclinic	orthorhombic	tetragonal
**Space group**	C2 (no. 5)	C2/c (no. 15)	Pca2_1 (no. 29)	P$4_3$22 (no. 95)
***a* / Å**	21.1288(4)	16.4470(3)	20.5083(2)	17.5541(2)
***b* / Å**	15.6506(2)	16.3182(3)	5.85452(6)	17.5541(2)
***c* / Å**	15.7898(3)	15.7208(3)	19.69462(18)	28.8897(6)
**α / °**	90	—	90	90
**β / °**	110.370(2)	116.481(3)	90	90
**γ / °**	90	—	90	90
***V* / Å^3**	4894.83(16)	3776.56(16)	2364.66(4)	8902.2(3)
Z	4	4 [b]	4	8 [c]
**ρ_{calcd} / g cm^{-3}**	1.324	1.434	1.659	1.345
**Radiation used**	Mo-Kα	Mo-Kα	Cu-Kα	Mo-Kα
**μ / mm^{-1}**	1.749	2.252	4.127	1.917
**2θ max / °**	65	65	145	57
**No. of unique reflns**				
**measured (R_{int})**	13409 (0.0268)	6441 (0.0236)	4385 (0.0305)	8967 (0.0209)
**obs, $\|F_o\| > 4\sigma(\|F_o\|)$**	11310	5592	4085	7011
**No. of variables**	505	207	253	537
**R_1(obs), wR_2(all) [a]**	0.0321, 0.0791	0.0261, 0.0648	0.0269, 0.0707	0.0390, 0.0857

Table S19 Crystal Data, Data Collection and Refinement Parameters for the structures of **1**, **2**, **4**, **6**. [a] $R_1 = \Sigma||F_o| - |F_c||/\Sigma|F_o|$; $wR_2 = \{\Sigma[w(F_o^2 - F_c^2)^2] / \Sigma[w(F_o^2)^2]\}^{1/2}$; $w^{-1} = \sigma^2(F_o^2) + (aP)^2 + bP$. [b] The complex has crystallographic Ci symmetry. [c] There are two independent C2-symmetric complexes.

**CCDC**	1027220
**Formula**	$C_{46}H_{66}Cu_4N_2$
**Formula weight**	901.16
**Colour, habit**	yellow blocks
**Crystal size / mm^3**	0.23 × 0.15 × 0.03
**Temperature / K**	173
**crystal system**	monoclinic
**Space group**	I2/a (no. 15)
***a* / Å**	16.7045(3)
***b* / Å**	24.7813(4)
***c* / Å**	21.8758(4)
**α / °**	90
**β / °**	101.2961(19)
**γ / °**	90
***V* / Å^3**	8880.2(3)
Z	8 [b]
**D_c / g cm^{-3}**	1.348
**Radiation used**	Mo-Kα
**μ / mm^{-1}**	1.922
**2θ max / °**	56
**No. of unique reflns**	
**measured (R_{int})**	9177 (0.0349)
**obs, $\|F_o\| > 4\sigma(\|F_o\|)$**	7227
**No. of variables**	562
**R_1(obs), wR_2(all)** [a]	0.0484, 0.0959

Table S20 Crystal Data, Data Collection and Refinement Parameters for the structure of **6b**. [a] $R_1 = \Sigma||F_o| - |F_c||/\Sigma|F_o|$; $wR_2 = \{\Sigma[w(F_o^2 - F_c^2)^2] / \Sigma[w(F_o^2)^2]\}^{1/2}$; $w^{-1} = \sigma^2(F_o^2) + (aP)^2 + bP$. [b] There are two independent C2-symmetric complexes.

9.1 The X-ray crystal structure of 1

The absolute structure of **1** was determined by use of the Flack parameter [0.037(9)].

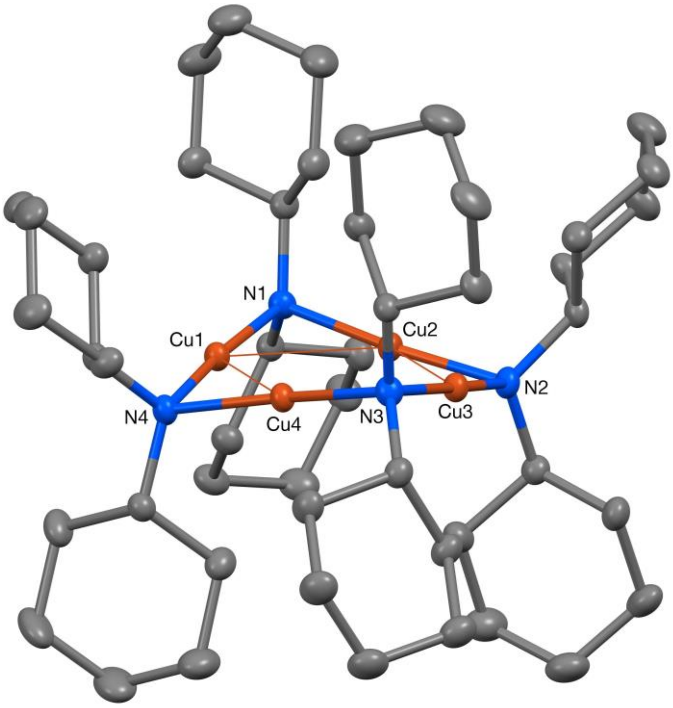

Figure S37 The crystal structure of **1** (50% probability ellipsoids).

9.2 The X-ray crystal structure of 2

The structure of **2** has crystallographic C_i symmetry about an inversion centre at the middle of the Cu_4N_4 ring.

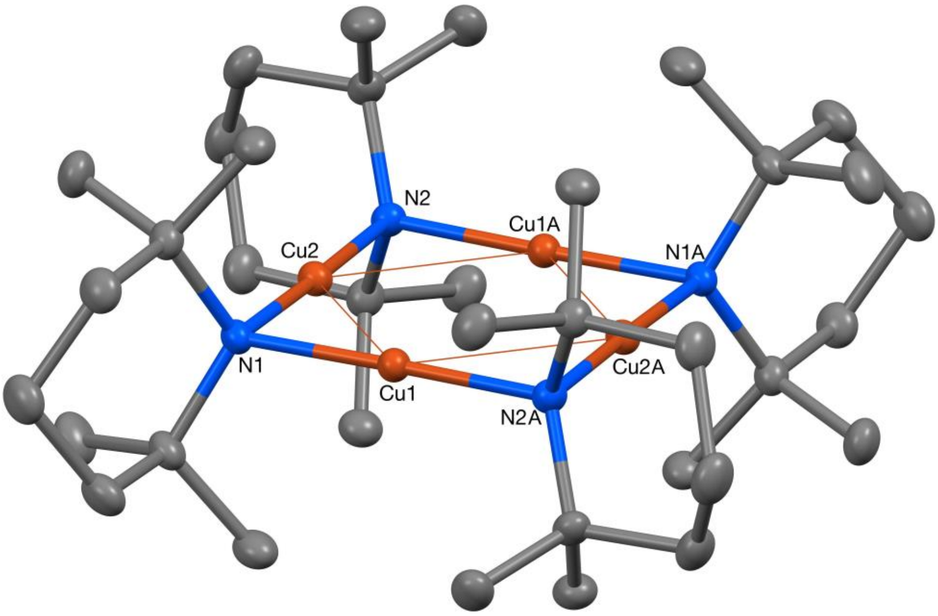

Figure S38 The crystal structure of the C_i-symmetric complex **2** (50% probability ellipsoids).

9.3 The X-ray crystal structure of 4

The absolute structure of **4** was determined by use of the Flack parameter [0.07(3)].

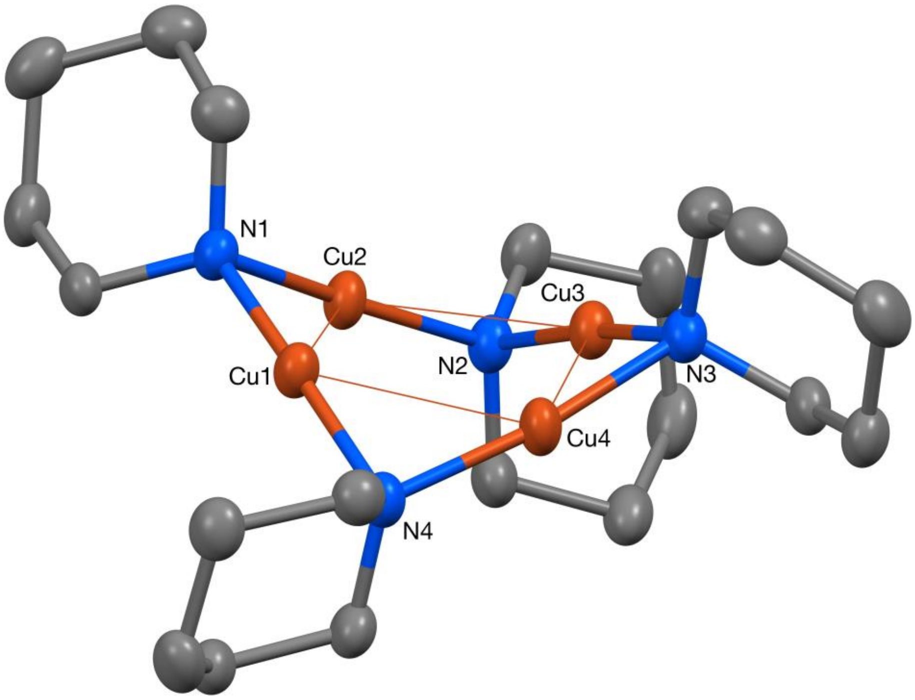

Figure S39 The crystal structure of **4** (50% probability ellipsoids).

9.4 The X-ray crystal structure of 6

The structure of **6** was found to contain two independent complexes (**6-i** and **6-ii**), each with C_2 symmetry. However, the two independent complexes differ in that whilst the Cu(1)-based molecule has the C_2 axis along the Cu(1)···Cu(2) vector (the copper atoms *not coordinated* to the piperidine ligands, *i.e.* the *short* diagonal of the Cu_4 rhombus), the Cu(4)-based molecule has the C_2 axis along the Cu(4)···Cu(5) vector (the copper atoms *coordinated* to the piperidine ligands, *i.e.* the *long* diagonal of the Cu_4 rhombus). For this latter case, a direct consequence of the position of the C_2 axis is that both the N(31) and N(61)-based piperidine ligands are inherently disordered. In each case this disorder was modelled by using one complete, geometry optimised, 50% occupancy orientation, with a second 50% occupancy orientation generated by action of the C_2 axis; the non-hydrogen atoms of the unique orientations were refined anisotropically. The N–H hydrogen atoms of the piperidine ligands in both independent complexes could not be located from ΔF maps and so were added in idealised positions at an N–H distance of 0.90 Å. The absolute structure of **6** was determined by use of the Flack parameter [0.070(11)].

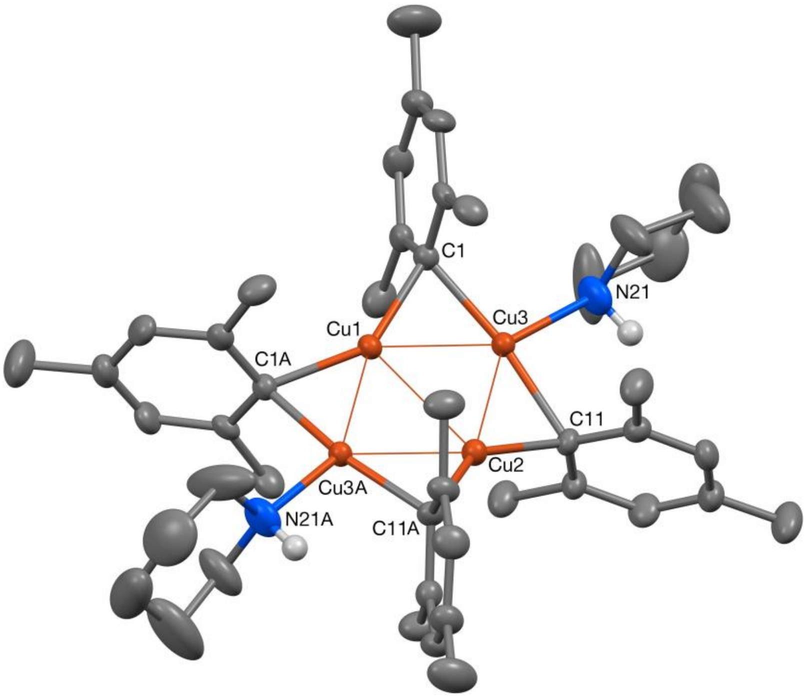

Figure S40 The structure of one (**6-i**) of the two crystallographically independent C_2-symmetric complexes present in the crystal of **6** (50% probability ellipsoids).

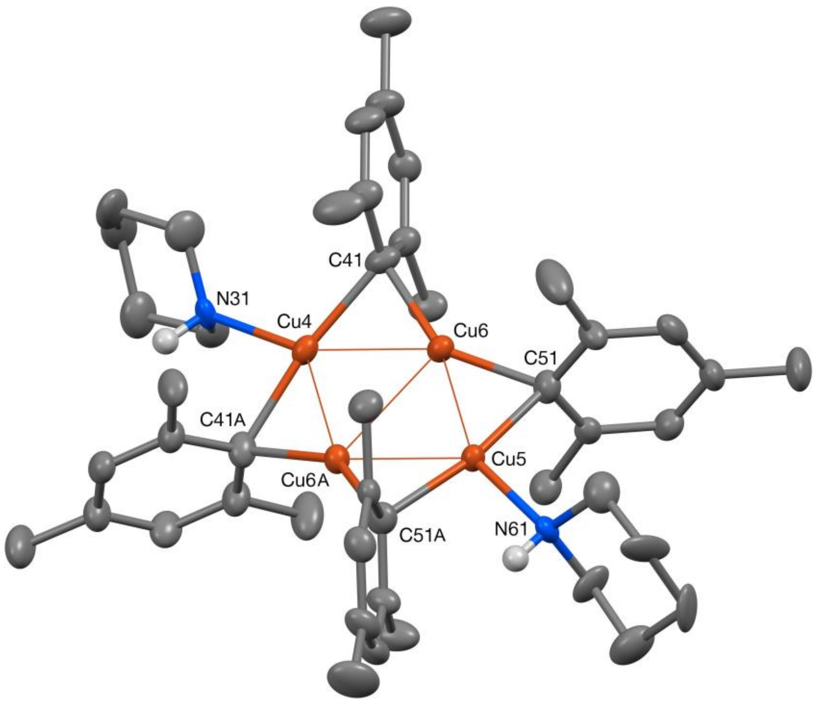

Figure S41 The structure of one (**6-ii**) of the two crystallographically independent C_2-symmetric complexes present in the crystal of **6** (50% probability ellipsoids).

9.5 The X-ray crystal structure of 6b

The structure of **6b** was found to contain two independent complexes (**6b-i** and **6b-ii**), each with C2 symmetry. However, the two independent complexes differ in that whilst the Cu(1)-based molecule has the C2 axis along the Cu(1)···Cu(2) vector (the copper atoms not coordinated to the piperidine ligands, i.e. the short diagonal of the Cu4 rhombus), the Cu(4)-based molecule has the C2 axis along the Cu(4)···Cu(5) vector (the copper atoms coordinated to the piperidine ligands, i.e. the long diagonal of the Cu4 rhombus). For this latter case, a direct consequence of the position of the C2 axis is that both the N(31) and N(61)-based piperidine ligands are inherently disordered. In each case this disorder was modelled by using one complete, geometry optimised, 50% occupancy orientation, with a second 50% occupancy orientation generated by action of the C2 axis; the non-hydrogen atoms of the unique orientations were refined anisotropically. Additionally, the N(21)-based piperidine ligand in the Cu(1)-based complex was also found to be disordered. Two orientations were identified of *ca*. 57 and 43% occupancy, their geometries were optimised, the thermal parameters of adjacent atoms were restrained to be similar, and only the non-hydrogen atoms of the major occupancy orientation were refined anisotropically (those of the minor occupancy orientation were refined isotropically). The N–H hydrogen atoms of the piperidine ligands in both independent complexes could not be located from ΔF maps and so were added in idealised positions at an N–H distance of 0.90 Å.

Despite being a very different polymorph, the molecular structure of **6b** is remarkably similar to that of **6**. As with the chiral tetragonal species, the structure of the racemic monoclinic species **6b** contains two independent C_2-symmetric molecules (**6b-i** and **6b-ii**) with one molecule having the C_2 axis along the short diagonal of the Cu_4 rhombus (the Cu(1)···Cu(2) vector) and the other having the C_2 axis along the long diagonal of the Cu_4 rhombus (the Cu(4)···Cu(5) vector). The geometries of the two molecules in **6b** are very similar both to each other and to the two independent molecules in **6**. The Cu_4 rhombi are perfectly flat as a result of the C_2 symmetry, and are substantially distorted away from square with unique interior angles of 67.32(2), 112.28(3) and 113.07(3)° for **6b-i**, and 67.35(3), 67.48(3) and 112.59(2)° for **6b-ii**. The Cu···Cu side lengths range between 2.4440(7) – 2.4526(6) Å, the short and long diagonals differ by ca. 1.36 Å due mainly to a substantial contraction in the short diagonal separation to 2.7126(9) and 2.7151(9) Å for Cu(1)···Cu(2) and Cu(6)···Cu(6A) respectively. Associated with this are significant distortions from linear for the C–Cu–C moieties at the copper atoms involved in the short diagonals with angles of 142.0(2), 143.2(2) and 142.52(18)° at Cu(1), Cu(2) and Cu(6) respectively cf. angles of 169.46(16), 169.3(2) and 169.8(2)° at Cu(3), Cu(4) and Cu(5). The Cu4C4 ring has a folded geometry in both **6b-i** and **6b-ii** with ca. 36° folds about both the C(1)···C(11A) and C(41)···C(51A) vectors.

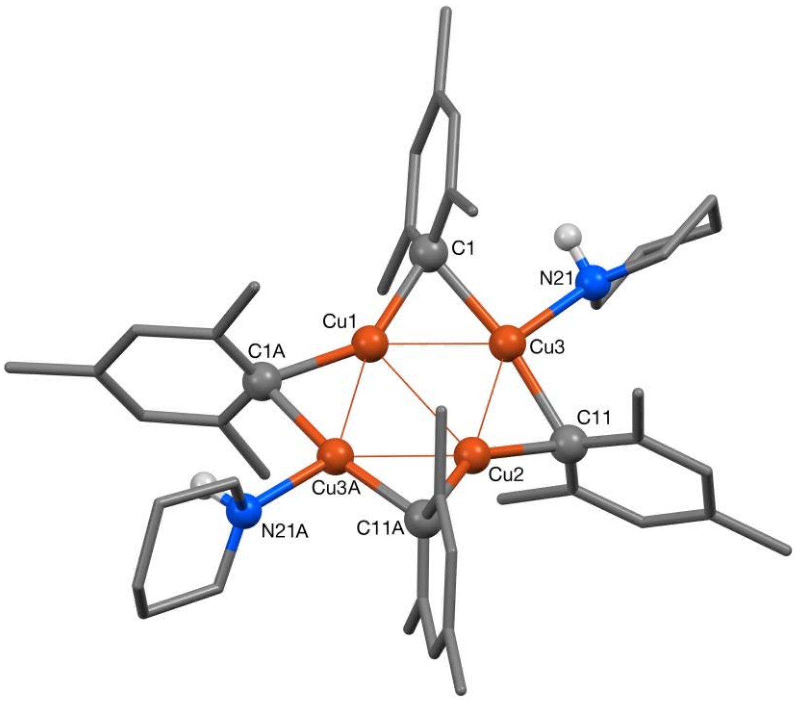

Figure S42 The structure of one (**6b-i**) of the two crystallographically independent C_2-symmetric complexes present in the crystal of **6b**

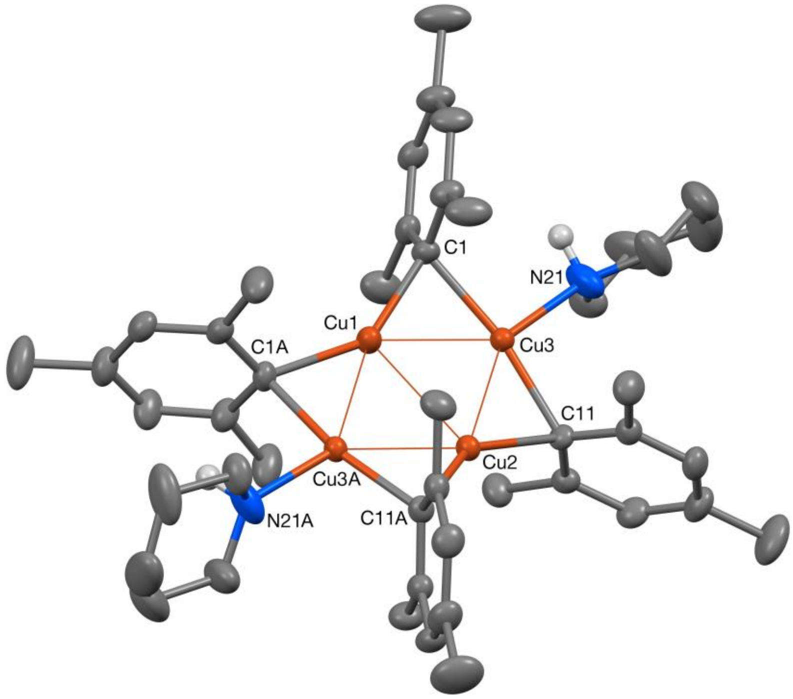

Figure S43 The structure of one (**6b-i**) of the two crystallographically independent C_2-symmetric complexes present in the crystal of **6b** (50% probability ellipsoids).

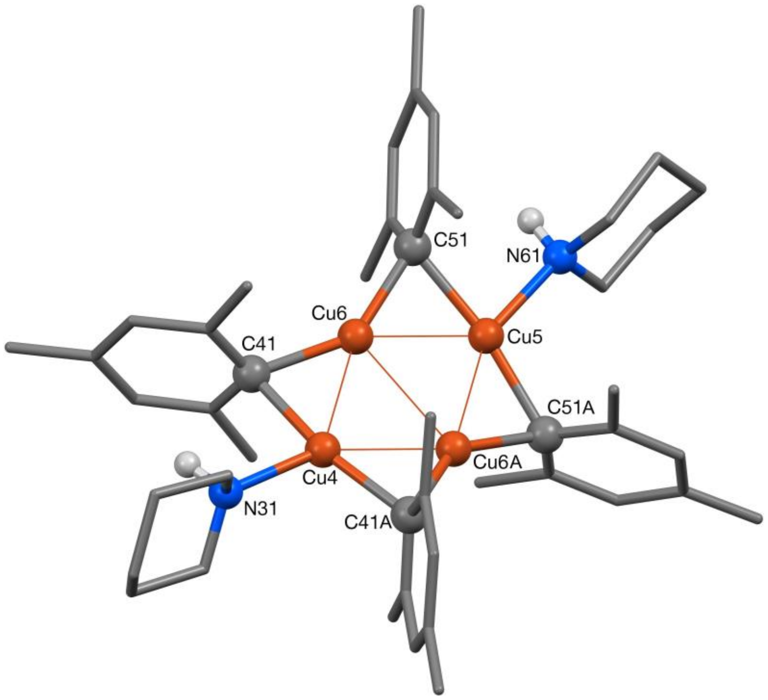

Figure S44 The structure of one (**6b-ii**) of the two crystallographically independent C_2-symmetric complexes present in the crystal of **6b**

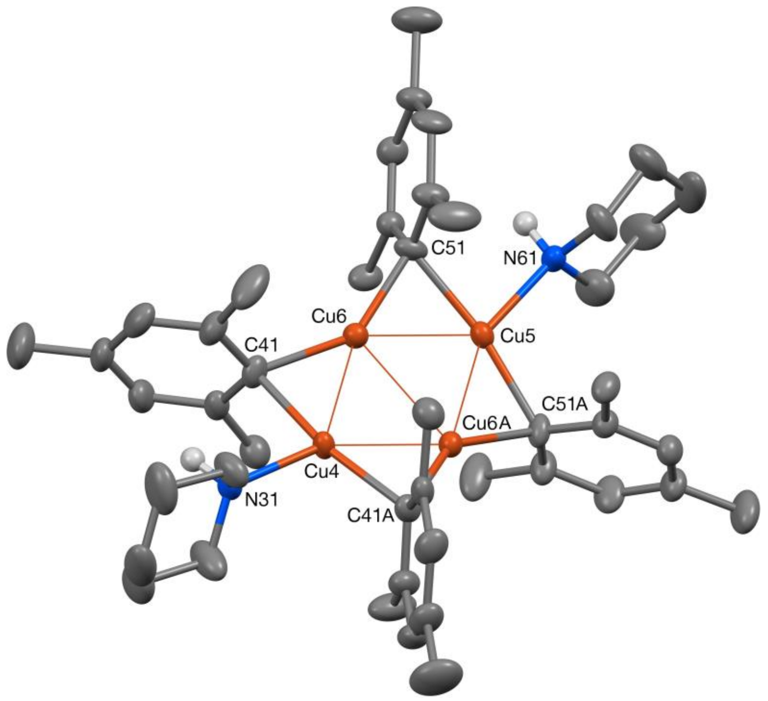

Figure S45 The structure of one (**6b-ii**) of the two crystallographically independent C_2-symmetric complexes present in the crystal of **6b** (50% probability ellipsoids).

10 References

[1] T. Tsuda, T. Yazawa, K. Watanabe, T. Fujii, T. Saegusa, *J. Org. Chem.* **1981**, *46*, 192–194.

[2] SHELXTL, Bruker AXS, Madison, WI.

[3] G. M. Sheldrick, *Acta Cryst.* **2008**, *A64*, 112–122.

[4] SHELX-2013, http://shelx.uni-ac.gwdg.de/SHELX/index.php.
